# Neuron-Like Networks Between Ribosomal Proteins Within the Ribosome

**DOI:** 10.1038/srep26485

**Published:** 2016-05-26

**Authors:** Olivier Poirot, Youri Timsit

**Affiliations:** 1 Information Génomique et Structurale CNRS, Aix-Marseille Université, UMR7256 FR-13288 Marseille, France

## Abstract

From brain to the World Wide Web, information-processing networks share common scale invariant properties. Here, we reveal the existence of neural-like networks at a molecular scale within the ribosome. We show that with their extensions, ribosomal proteins form complex assortative interaction networks through which they communicate through tiny interfaces. The analysis of the crystal structures of 50S eubacterial particles reveals that most of these interfaces involve key phylogenetically conserved residues. The systematic observation of interactions between basic and aromatic amino acids at the interfaces and along the extension provides new structural insights that may contribute to decipher the molecular mechanisms of signal transmission within or between the ribosomal proteins. Similar to neurons interacting through “molecular synapses”, ribosomal proteins form a network that suggest an analogy with a simple molecular brain in which the “sensory-proteins” innervate the functional ribosomal sites, while the “inter-proteins” interconnect them into circuits suitable to process the information flow that circulates during protein synthesis. It is likely that these circuits have evolved to coordinate both the complex macromolecular motions and the binding of the multiple factors during translation. This opens new perspectives on nanoscale information transfer and processing.

Ribosomes are large ribonucleoprotein particles that catalyse the mRNA-directed protein synthesis^1^. One of the most surprising features of ribosome structures was the finding that ribosomal proteins possess long filamentous and irregular extensions that penetrate deeply into the RNA core^2^,^3^,^4^. These extensions display features of intrinsically disordered proteins^5^ and have been thought to play a role in inter-protein communication^6^,^7^ or in ribosome assembly^8^,^9^,^10^. However, the molecular mechanisms underlying these putative functions are still poorly understood. The finding of two folding states in the crystal structure of the ribosomal protein bL20^11^ provided structural insights into the mechanism of signal transmission along an α-helical extension and stimulated us to search if similar properties are also observed in other ribosomal proteins. Here, a large-scale analysis of the ribosome particle structures of the three domains of life shows that an essential extension function is to connect distant ribosomal proteins.

## Results and Discussion

**Figure 1:**
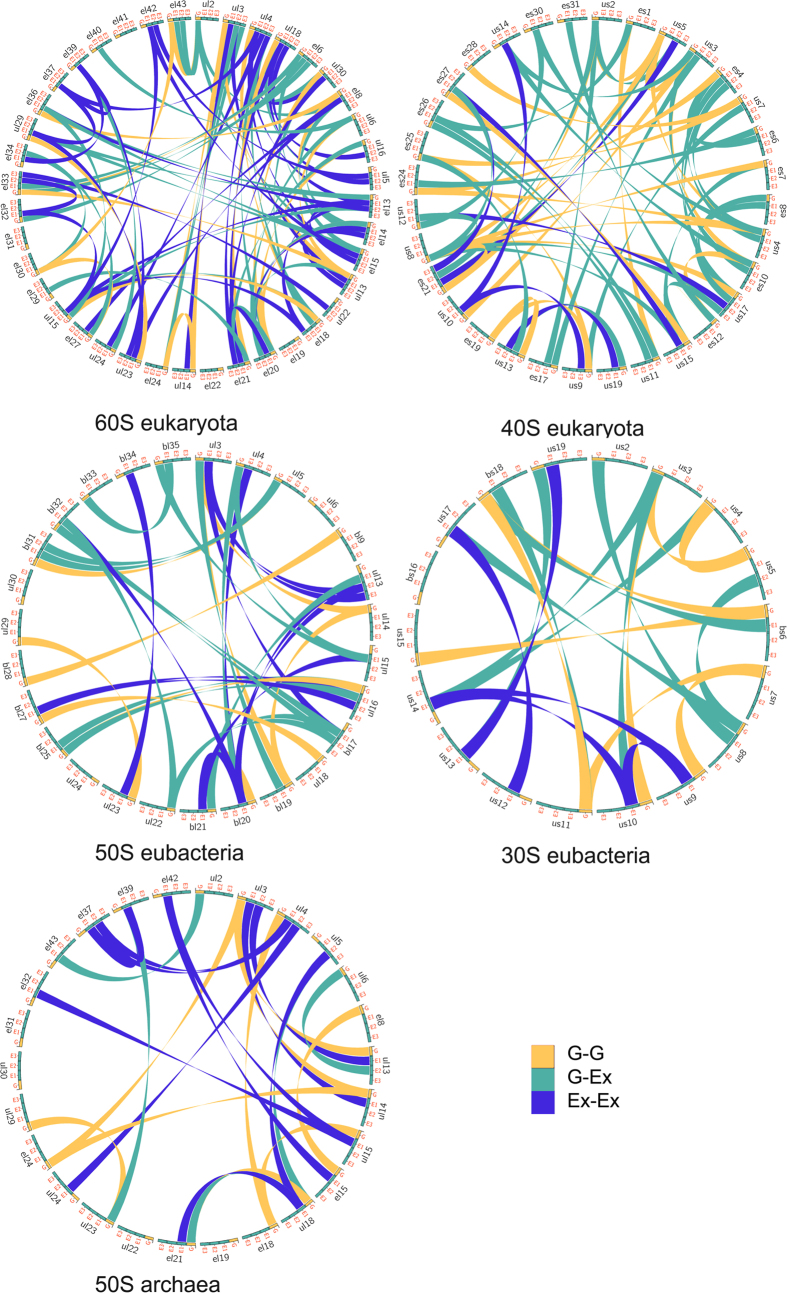
Ribosomal protein interaction networks in the three domains. *Circos* diagrams of the interactions between ribosomal proteins. Each type of interactions are represented by different colours listed in the inset legend according to the following codes: G-G: interactions between globular domains; G-Ex: interactions between a globular domain and an extension; Ex-Ex: interactions between extensions. This figure is a graphical representation of Table 1 and Supplementary Tables S3–S7. PDB identifiers of ribosome structure used for this analysis are 4v88 (eukarya), 4v8I (eubacteria) and 1s72 (archaea).

**Figure 2:**
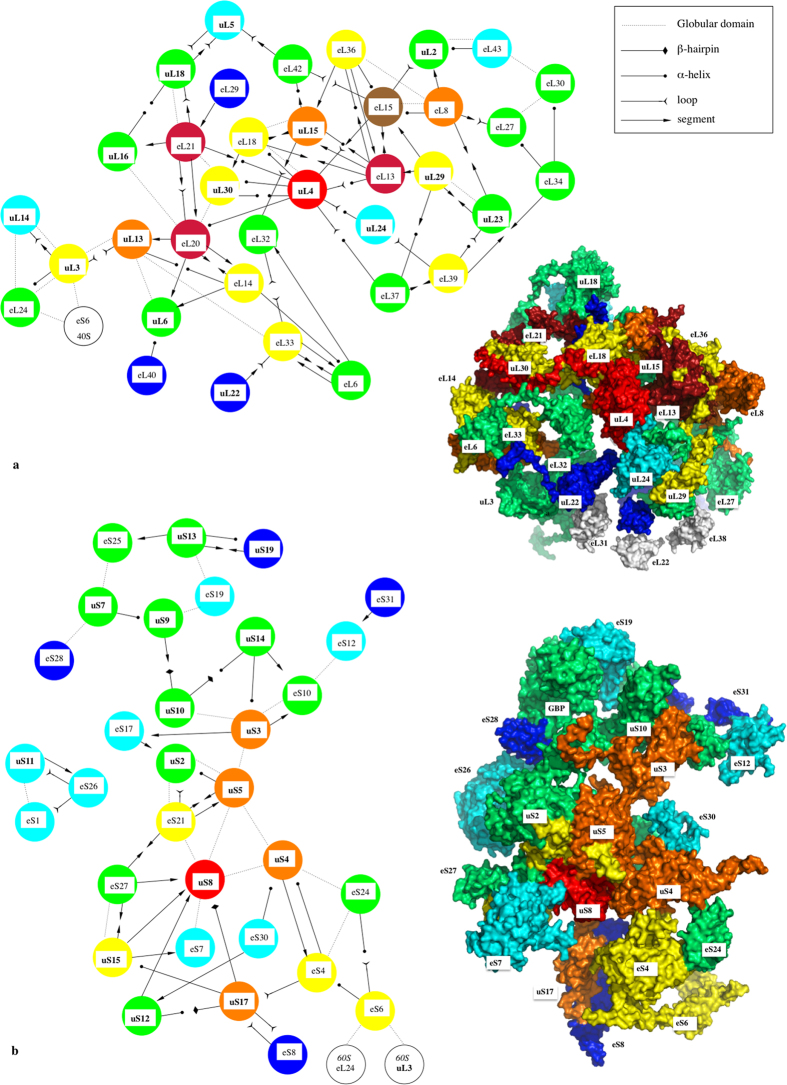
Assortativity of protein networks in eukaryotic ribosomal subunits. (**a**) 60S eukaryotic subunit (PDB id: 4v88); (**b**) 40S eukaryotic subunit (PDB id: 4v88). The proteins are coloured in function of their number of interacting partners. White: 0; blue: 1; cyan: 2; green: 3; yellow: 4; orange: 5; brown: 6; red brick: 7; red: 8 (see Supplementary Table S8). In each panel, a surface representation of the X-ray subunit structure and the corresponding 2D schematic representation of the ribosomal protein network are displayed. The schematic 2D diagrams of the networks also indicate the secondary structures involved in the interactions (Table 1, Supplementary Tables S3–S7) using an arrow code indicated into the legend box (the same colour and arrow codes are used for Fig. 3).

**Figure 3:**
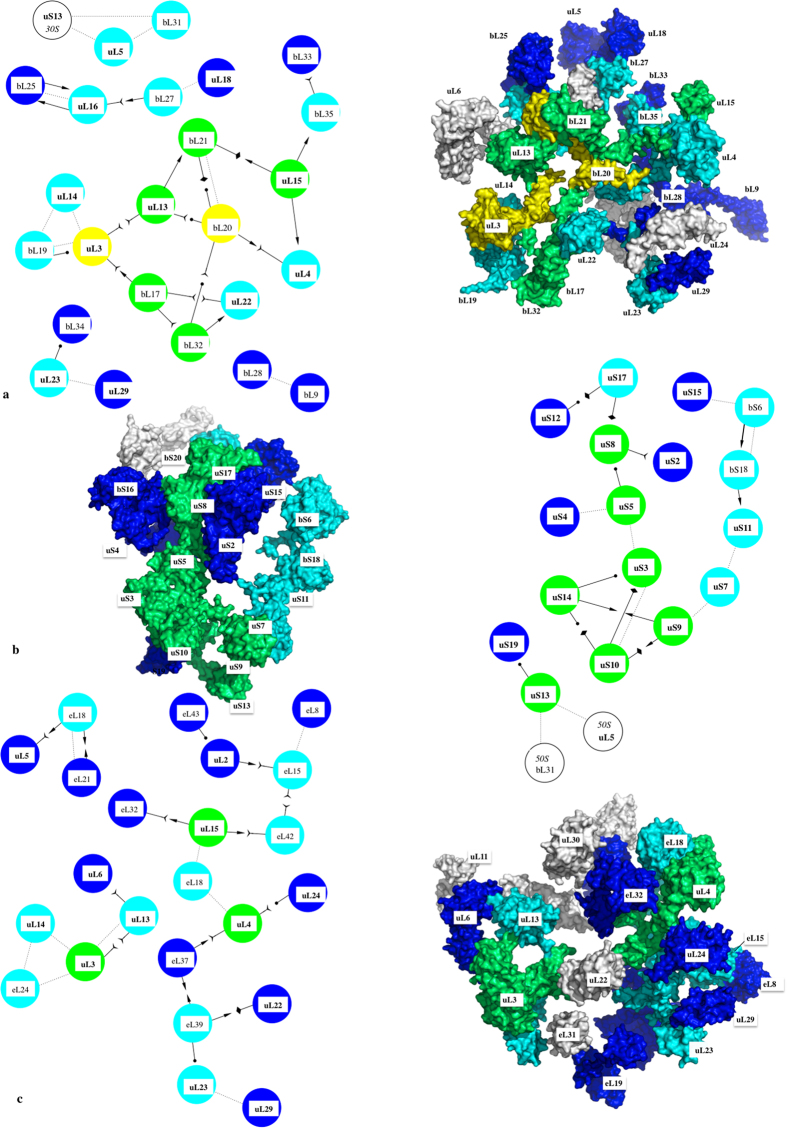
Assortativity of protein networks in eubacterial and archaeal ribosomal subunits. (**a**) 50S eubacterial subunit (PDB id: 4v8I); (**b**) 30S eukaryotic subunit (PDB id: 4v8I); (**c**) 50S archaeal subunit (PDB id: 1v72). See Fig. 2 for the colour and arrow codes.

The extensions that are observed in most of the ribosomal proteins systematically participate in networks with a great diversity of protein-protein interactions (Fig. 1, Table 1; Supplementary Tables S1–S7 and Figs S1–S3). Reciprocally, we show that the proteins that are not involved in protein-protein interactions are devoid of extensions. Also, from archaea to eukaryotes the number of inter-protein contacts greatly increases with the extension sizes and the number of extensions per protein (Table 1). The eukaryotic ribosome displays the highest number and diversity of inter-protein contacts. For a total of 80 protein-protein interactions in the 60S ribosomal subunit of eukaryotes, 62 are mediated through extensions that connect either the other extensions or the globular domains of their partners. Also, all the proteins of the 40S eukaryotic subunit and most of the bacterial and archaeal extensions participate in inter-protein contacts. All kinds of possible contacts between the different categories of extensions are observed in the three domains (Table 1, Figs 2 and 3). Direct contacts between globular domains are far less frequent than those involving extensions, thus supporting the hypothesis that extensions have evolved to connect proteins that are too distant to interact directly by their globular domains.

Secondly, our analysis shows that these networks present an interesting similarity with information processing networks (Figs 2 and 3; Supplementary Tables S8–S10 and Fig. S4). In the three domains, the proteins form scale-free and assortative networks containing highly connected hubs^12^. Eukaryotic 60S proteins form a single network, with most of the proteins connected to 3 or 4 partners. Through their multiple extensions, hubs such as eL15 and uL4 connect 7 and 8 partners that also belong to the most connected proteins (Fig. 2a; Supplementary Table S8). Similarly, uS8 interacts with 8 highly connected partners within the 40S subunit (Fig. 2b). Although less inter-connected, the bacterial and archaeal proteins also form assortative networks. For example, the eubacterial hubs uL3 and bL20 that are essential for 50S assembly interact with the most connected proteins (Fig. 3a; Supplementary Fig. S4). Another property of these networks is that they relate proteins whose globular domains are located on opposite sides of the subunits with only a few inter-node links. In addition, we observe network-motifs well known to participate in information processing, such as the feed-forward loops in the eukaryotic subunits (Supplementary Tables S9 and S10)^13^. Interestingly, as indicated by the number of inter-protein contacts/rRNA size ratio that is significantly higher in eukarya (Table 1), the ribosomal network connectivity is not simply proportional to the rRNA size but seems rather to reflect the growing ribosome complexity during evolution.

**Figure 4:**
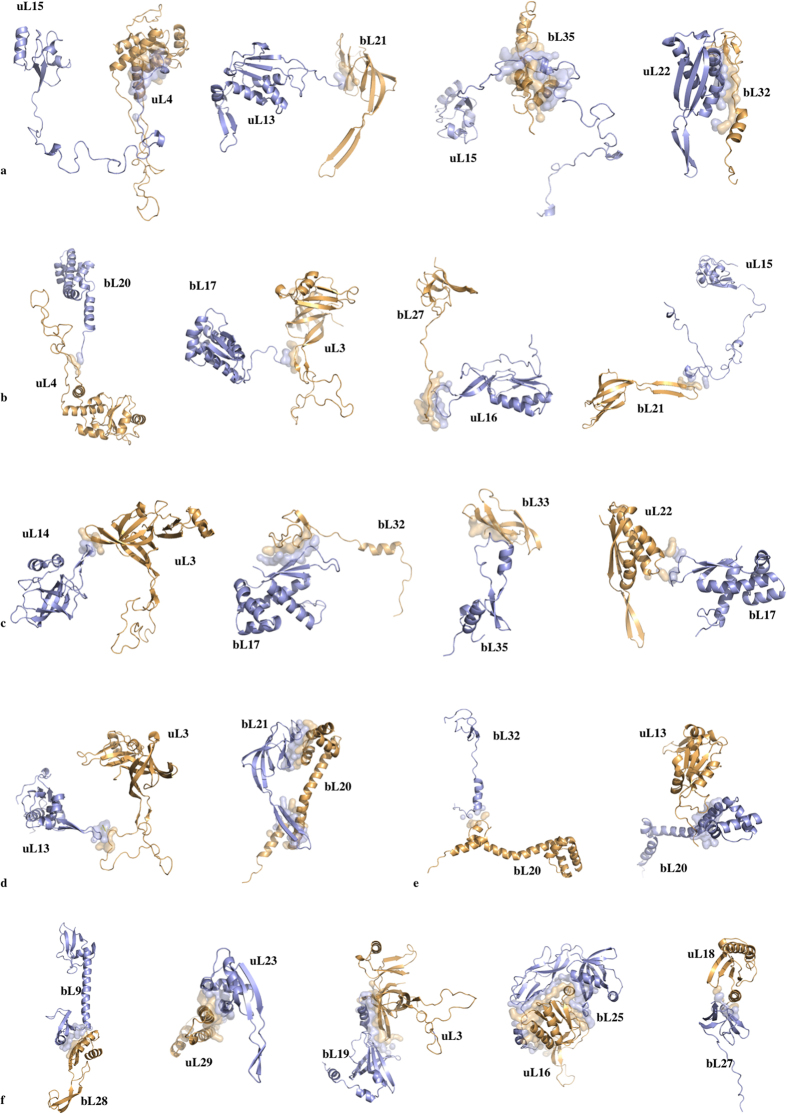
Cartoon representation of interacting protein pairs of the eubacterial 50S subunit (PDB id: 4v8i). The interfaces are represented by transparent surfaces. (**a**) SG (protein segment-globular domain). Top: chain extremity – globular domain; Bottom: lateral interaction between protein segment and globular domain. (**b**) SL (protein segment-loop), bottom right: interaction of a segment with a β-hairpin. (**c**) LG (loop – globular domain). (**d**) left: LL (loop-loop); right: BH (β-hairpin-α-helix). (**e**) LH (loop-α-helix). (**f**) GG (globular domain-globular domain).

**Figure 5:**
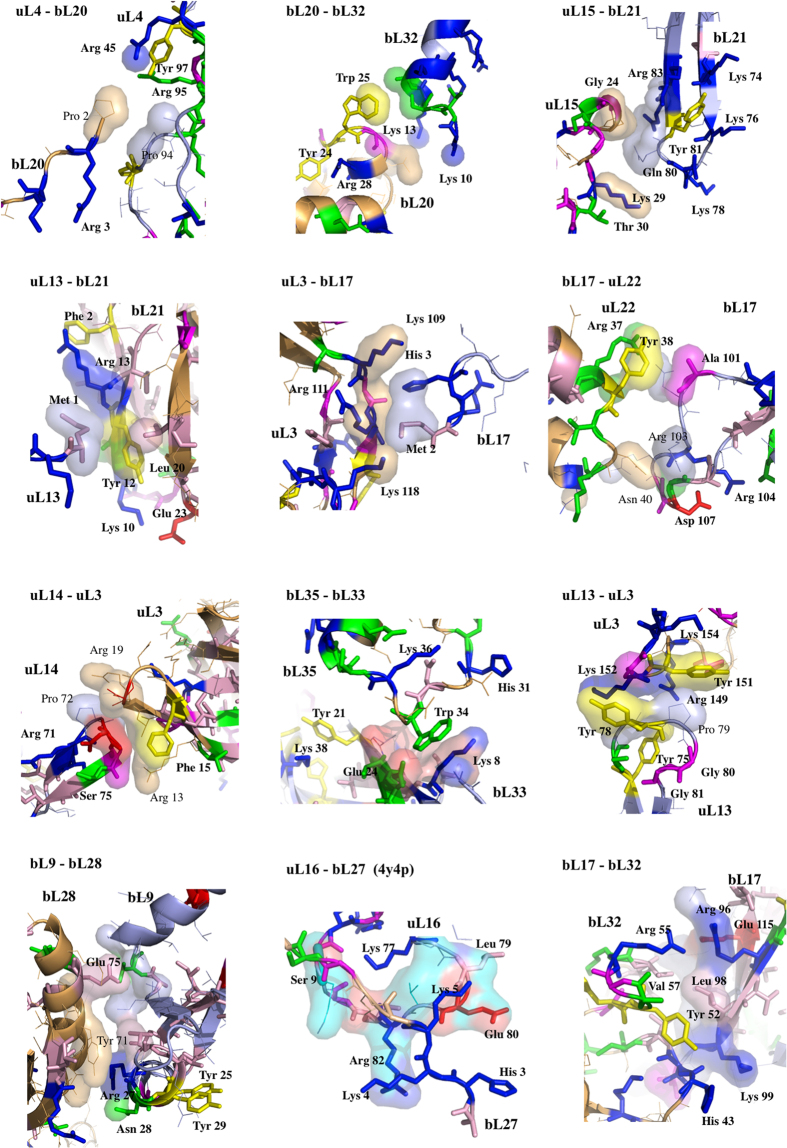
First set of protein-protein interfaces of the 50S ribosomal protein network (pdb entry 4v8i; fully ordered uL16-bL27 is from pdb entry 4y4p). Conserved amino acids are represented with thick coloured sticks; blue: basic, red: acidic; yellow: aromatic; pink: hydrophobic; green: polar.

**Figure 6:**
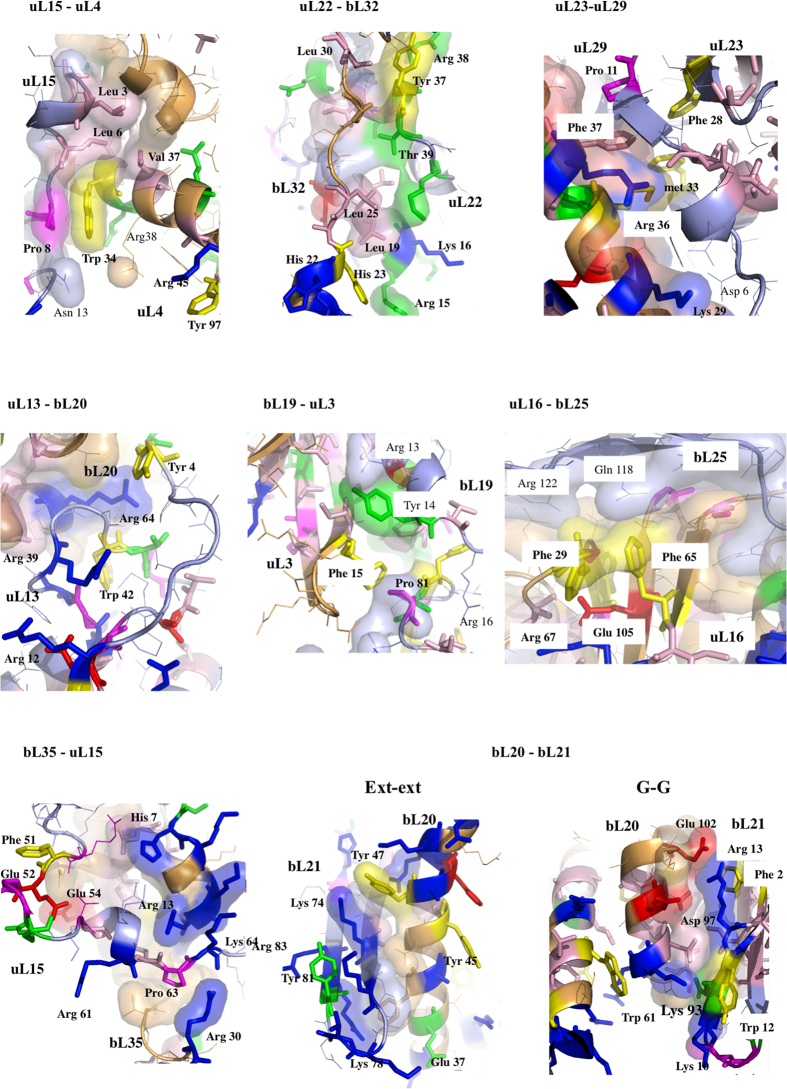
Second set of protein-protein interfaces of the 50S ribosomal protein network (pdb entry 4v8i). The colour scheme is the same than in Fig. 5.

Thirdly, to decipher the underlying molecular mechanisms of information transmission within these networks, all the inter-protein contacts of the eubacterial large subunit have been systematically analysed. This network indeed provides the opportunity to compare and analyse the highest number of equivalent structures from three eubacterial species (*E. coli, D. radiodurans and T. thermophilus*) crystallized in different functional translation steps, initiation, elongation and termination with various factors and antibiotics^1^ (Supplementary Table S11). Most of the interface contact areas are unusually small, with values well below those classically expected for forming stable protein interactions^14^ (Table 2, Fig. 4). These interfaces are so minute, that they seem to have been systematically overlooked in previous studies. However, our analysis shows that they are phylogenetically and structurally well conserved, suggesting that they are likely to play an essential function. Indeed, sequence alignments provided by Y. Wolf^15^ indicate that, except in a few cases (uL16-bL25 and bL9-bL28), most of the interfaces contain highly conserved key amino acids, mainly aromatic and basic that establish specific intermolecular interactions in all the eubacterial species (Figs 5 and 6; Supplementary Table S12 and Fig. S5). All the recorded miniature inter-protein contacts have been systematically observed in the 50S structures of the three analysed species. Their comparison within all the available 50S and 70S structures whose resolution is higher than 3.5 Å shows that each interface exhibits a unique structure whatever the species or the translation steps in which the ribosomes have been crystallized (Supplementary Table S11). A notable exception is the uL16-bL27 pair that becomes fully ordered when the P-tRNA site is occupied by the P-tRNA (See Methods).

**Figure 7:**
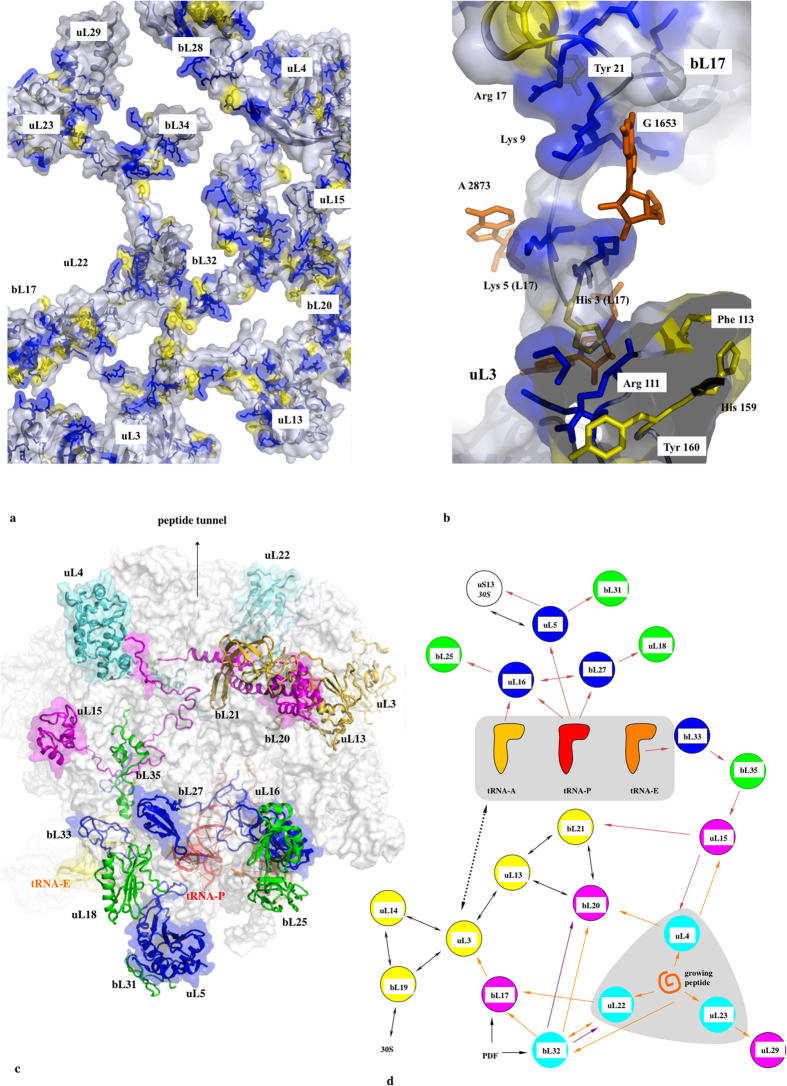
Information flow within the 50S eubacterial subunit. (**a**) Surface representation of the interconnected proteins of the 50S ribosomal subunit. The aromatic residues are coloured in yellow. The basic residues that are located at 7 Å or the aromatic side chains are represented in blue sticks. (**b**) Detail of the uL3-bL17 interconnection. The aromatic and basic residues are represented in yellow and blue sticks, respectively. The rRNA bases that participate to the cation-π interactions are represented with orange stick. (**c**) View of the network in which proteins are coloured according to the functional sites: blue cartoons: tRNA site “sensory-proteins”; green cartoons: the first layer of proteins interacting with tRNA sensory-proteins; cyan cartoons: peptide tunnel “sensory-proteins”; magenta cartoons: the first layer of proteins that interact with peptide tunnel “sensory-proteins”; yellow cartoons: inter-proteins. (**d**) Schematic representation of the network with the colour code used in (**c**). The coloured arrows represent the possible transmission pathways between the sensory-proteins and the processing inter-proteins.

Remarkably, except uL16-bL27, all 50S protein-protein interfaces, from the smallest (3 aas, 25 Å^2^) to the largest (48 aas, 1709 Å^2^), contain conserved residues that are systematically involved in one and most frequently several intra- or inter-molecular basic-aromatic amino acid interactions^16^ (Table 2, Figs 5 and 6). In addition, in more than half of them, these cation-π interactions have been found in close vicinity to salt-bridges. Thus, the interfaces are split in two main groups: (i) cation-π, and (ii) the cation-π/salt-bridge groups. In a few cases such as uL15-uL4, uL23-uL29 and uL3-bL19, proline-aromatic amino acid interactions are also observed^17^ (Table 2). The largest interfaces (from 400 to 1700 Å^2^) are further stabilised by additional intermolecular contacts such as hydrophobic interactions and hydrogen bonds that are usually observed in classical dimers. The major finding of this analysis is that the smallest interfaces display a “necessary minimum” that is also shared by larger ones: the conserved aromatic-basic amino acid interactions. Too tiny to be rationalized in terms of dimer stabilisation, these highly conserved interfaces have probably been selected during evolution to play a specific role in inter-protein communication. They reveal the strictly necessary interacting residues to ensure information transfer from a protein to another. Indeed, it has been experimentally shown that cation-π interactions mediate inter-domain communication in proteins^18^, and participate in information transfer in the central nervous system by mediating the interactions between the neurotransmitters and their receptors^16^. Particularly interesting is the observation that these basic-aromatic amino acid interactions also spread without interruption along the extensions and form a regularly distributed array of intra-molecular interactions along the whole protein network (Fig. 7a). RNA bases have been also found to substitute the aromatic residues when they are lacking, to ensure continuity in the interaction network, as found for example within the bL17-uL3 connecting segment (Fig. 7b).

The ribosomal wires are reminiscent of DNA^19^ or bacterial nanowires^20^ known to perform metal-like charge transfer through the π-orbitals of the DNA bases or the periodic arrays of aromatic residues. It would be interesting to test if ribosomal wires are able to transmit an electric signal, by linking distant proteins of the network within an intact ribosome, to the luminescent probes developed to monitor charge transport along DNA^19^. Alternatively, the ribosomal wires may use a still unknown mechanism for signal transduction that involves an array of contiguous cation-π interactions. One could imagine that electrostatic perturbations induced by the binding of tRNAs or translation factors could be propagated as a wave along the wire.

Viewing ribosomal protein extensions as connecting wires that play a role in information transfer is consistent with experimental data showing that RNA^21^,^22^ and some proteins^23^,^24^,^25^ participate in allosteric coordination and information exchange between distant parts of the ribosome. In addition, our study provides new structural insights to decipher the molecular mechanisms of allosteric communication within disordered proteins^26^,^27^. Since the extensions and interfaces of the small and large ribosomal subunits of the three cellular domains have similar sequence and structural features, it is likely that they all share the property of forming cross-talk networks. Eubacterial ribosomal communication pathways such as the uL13-uL3 interface are indeed universally conserved across the three domains of life, while others are replaced by convergence in archaea and eukaryota (Supplementary Figs S6–S9). Particular triplet motifs are also recurrently observed in the three domain’s ribosomes, implying they might play a specific function.

It could be asked why highly interconnected ribosomal protein networks have evolved with a growing complexity during evolution, since RNA can also ensure allosteric communication. During translation, ribosomes orchestrate the binding of mRNA, tRNAs and multiple translation factors^28^. Each of the three main translation stages (initiation, elongation and termination) requires the sequential binding and release of specific translation factors. During the elongation cycle the ribosomes coordinate complex movements such as the coupled translocation of tRNAs and mRNA that are associated with large-scale structural rearrangements^29^. The peptide bond formation and the extrusion of the nascent peptide through the exit tunnel must be also coordinated with the tRNA and mRNA translocation. In addition, in order to coordinate cellular responses to environmental changes, the ribosome activity is regulated by factors that adapt translation to cell activity. The recent structure of BipA, a GTPase involved in bacterial stress response, bound to the ribosome in its active state has provided new insights about these processes^30^.

It is likely that this complex molecular machine should require a system of information transfer and processing to coordinate its multiple tasks and sequential movements. From a functional point of view, our study shows that the 50S protein network is indeed organized along the path of tRNA translocation and around the peptide exit tunnel (Fig. 7c,d). Similar to an organisation into sensory- and inter-neurons connections in simple brains^31^, the sensory-ribosomal proteins uL16, bL27, uL5, bL33 literally “innervate” the three key A, P and E-tRNA sites while uL4, uL22 and uL23 sense the interior of the peptide exit tunnel^32^ through their extensions. It has been shown that the uL16-bL27 interface may behave as an electrostatic sensor that becomes fully ordered when the P-tRNA is bound. On the other hand, inter-neuron-like proteins interconnect proteins that sense distant functional sites. Ribosomal proteins therefore display at a nano-scale the characteristics of “molecular neurons” interacting together through “molecular synapses”. This neural-like organisation strongly suggests that the network has the property to not only transfer but also process the information coming from distant functional ribosomal sites. In a preliminary model, we propose the 50S protein network processes the information flow that circulates between the tRNA binding sites and peptide tunnel to coordinate the complex tasks and motions during protein synthesis (Fig. 7d). One could speculate that information processing ability is restricted to protein circuits, thus explaining why they have evolved to complement allosteric “transfer only” rRNA networks. The ribosomal protein networks perhaps constitute some of the most striking examples of “proteins as computing element in the cell” as formulated by D. Bray, twenty years ago^33^.

## Methods

All the structures of eukaryotic, eubacterial and archaeal ribosomes deposited in the protein data bank (PDB)^34^ have been systematically analysed in order to characterize their extensions and their inter-proteins contacts. The nomenclature of ribosomal proteins used in the tables and figures follows the a new system for naming ribosomal proteins that has been recently adopted^35^ (see also the site of N. Ban laboratory: http://www.bangroup.ethz.ch/research/nomenclature-of-ribosomal-proteins.html). The tables for the conversion between the old and the new system are provided in Supplementary Tables S13 and S14. The extensions have been defined as elongated protein chains protruding from the globular domain. Segments (seg) are extensions from the N-terminus or the C-terminus ends devoid of secondary structure, mixed extensions (mix) combine helical (H) and unstructured chains (S). Structured extensions contain α-helices (H), loops (L) or β-hairpins (B). Interactions between extensions or between extensions and globular domain have been systematically detected with the program *pymol*^36^ when inter-molecular distances are less or equal to 4 Å.

### Network characterisation

Scripts have been written for systematically mapping the protein-protein interactions within the ribosomal subunit for display by the program *circos*^37^. The ribosomal protein networks of the large and small subunits of the three domains have been characterized according to the parameters defined in Barabasi *et al.*^12^. Network motifs detection in non-directed networks has been performed with the program *mfinder* provided by U. Alon^13^.

### Sequence alignments, interface characterization and structural analysis

The sequence alignments of all the eubacterial ribosomal proteins have been kindly provided by U. Wolf and N. Yutin^15^. They can be downloaded from the site: ftp://ftp.ncbi.nih.gov/pub/wolf/_suppl/ribo. The aligned sequences have been visualized and analysed with the program *Jalview*^38^. Consensus sequences and conserved residues of the ribosomal proteins at the interface area have been represented in Supplementary Fig. S5, with the “zappo” colouring scheme and in Supplementary Table S12. Conserved residues have been integrated in *pymol* scripts for their three-dimensional representation and zappo colouring (Figs 5 and 6).

### Structural analysis of the interfaces

The structures of each interacting protein pairs have been analysed in all the available PDB crystallographic structures of three species (*T. thermophilus, D. radiodurans* and *E. coli)* 50S and 70S subunits whose resolution is greater than 3.5 Å (Supplementary Table S11). *Pymol* scripts have been developed to systematically superimpose together all the equivalent protein models in order to compare them at the interface regions. Although homologous 50S proteins superimpose extremely well, twelve interfaces have been found to differ from one structure to the next (Supplementary Table S15 and Fig. S10). Knowing that in the resolution range (2.9–3.5 Å) misinterpretation of electron density maps in unclear regions are possible, we have carefully checked the reliability of these different models by the systematic analysis of their electron density maps. The program *Phenix*^39^ has been automated to compute 2fo-fc and difference maps of all the models listed in Supplementary Tables S11 and S15, from their structure factors deposited into the PDB. *Pymol* scripts have been developed to systematically compare the maps at the sites where structural difference have been observed. Based on the high quality of electron density maps of recent high resolution structures (4v8i, 4y4o, 4ybb, 4z8c, 4w2h, 4w2f, 4y4p) and the careful comparison of the density maps, our analysis has revealed that in most cases, the conformation “x” (coloured in red in Supplementary Table S15 and reported Supplementary Fig. S10) corresponds to misinterpretations of poorly resolved maps in these regions (Supplementary Fig. S11). For example, our analysis reveals that the residues built in the alternative conformation “x” have been in fact, fitted into density peaks corresponding to neighbouring residues (see legend of Supplementary Fig. S11). We deduced that the all the of the 50S subunit interfaces display a unique conformation whatever the species, the crystallization conditions, the translation factor and antibiotic added or the translation step in which the ribosome has been crystallized. The protein interfaces are both phylogenetically and structurally extremely well conserved. Consequently, we have focused our structural analysis on reliable high-resolution structures (*T. thermophilus*: 4v8i, 4w2f, 4w2h, 4z8c, 4y4o, 4y4p; *E. coli*: 4ybb and *D. radiodurans*: 5dm6).

The protein pair interfaces extracted from these models have been characterized with *areaimol* from the CCP4 program suite^40^. The analysis of intermolecular interactions at the interfaces has been performed by visual inspection of the interface structures and by using the web server *PIC*^41^. Ribosomal subunits of archaea, eubacteria and eukaryota have been also systematically superimposed in order to compare their networks and connections. All the scripts that have been developed in this study can be sent by mail on demand.

## Additional Information

**How to cite this article**: Poirot, O. and Timsit, Y. Neuron-Like Networks Between Ribosomal Proteins Within the Ribosome. *Sci. Rep.* **6**, 26485; doi: 10.1038/srep26485 (2016).

## Supplementary Material

Supplementary Information

## Figures and Tables

**Table 1:** Statistics of extension numbers, types and interactions observed in the large and small ribosomal subunits of the three domains (ribosomal subunit PDB identifiers for eukarya: 4v88, eubacteria: 4v8i, archaea: 1s72).

	60S euk.	40S euk.	50S eubact.	30S eubact.	50S archaea
**Total number of proteins**	**41**	**32**	**29**	**19**	**29**
**Tot nb. of prot. with extension**	**34 (83%)**	**27 (84%)**	**20 (69%)**	**13 (68%)**	**18 (62%)**
1 ext.	14	20	15	11	13
2 ext.	17	4	4	2	5
3 ext.	3	3	1	0	0
without extension	7	5	9	6	11
**Total number of extensions**	**57**	**37**	**26**	**15**	**23**
Nb and average length (aas) of extensions					
Segment	12 (22.3)	13 (21.6)	8 (23.3)	6 (15)	5 (28.6)
Mix	22 (50.9)	13 (45.5)	5 (39.2)	3 (45)	3 (47.3)
Loop	16 (27)	7 (23)	7 (27)	1 (19)	12 (27.8)
Helix	6 (33.5)		2 (25)	1 (8)	2 (16.5)
β-HP	1 (21)	4 (21)	4 (19.8)	4 (16)	1 (16)
**nb. of ext. involved in contacts**	**54**	**31**	**22**	**12**	**19**
nb. of ext. not involved in contacts	3	6	4	3	4
nb. of interacting proteins	37	32	25	19	29
**Type of contacts**					
***SG***	16	15	6	2	
***HG***	12	8	1	3	2
***LG***	3	5	3	1	2
***BG***		1		2	
***SL***	8		3		5
***HL***	4	1	2		1
***SH***	5			1	
***HH***	3				
***BH***		2	1	2	
***BS***		1	1	1	
***BL***					
***LL***	3		1		
***SS***	9	4		1	2
**Nb of interactions involving extensions (ext-G, ext-ext)**	**63**	**37**	**18**	**13**	**12**
**Nb of interactions between globular domains**	18	20	12	8	9
**Tot nb of interactions**	**81**	**57**	**30**	**21**	**21**
**Tot nb of interactions/100nt rRNA**	**2.2**	**3.2**	**1**	**1.3**	**0.7**

**Table 2:** Structural properties of the protein interfaces within the eubacterial 50S subunit (pdb entry 4v8i).

	Protein pair	Contact Type	Nb. Interf. Res.	Nb. Cons. Res.	ΔASA	Base-aromatic	Salt-Bridge	Pro-arom Or Pro-basic
1	**uL4-bL20**	LS	3-1	2-1	25	**Intra uL4arg 45 (uL4)-tyr 97 (uL4)**arg 95 **(uL4)-tyr 97 (uL4)**		**Interarg 45 (uL4)**-pro 2 (bL20)
2	**bL20-bL32**	HH	4-4	2-4	68	**InterTrp 25 (bL20)-Lys 13 (bL32)Trp 25 (bL20)-Arg 15 (bL32)Intra bL20Trp 25 (bL20)-Arg 28 (bL20)**		
3	**uL13-bL21**	SG	1-4	1-4	74	**Intra bL21Arg 13 (bL21)-Phe 2 (bL21)Tyr 12 (bL21)-Lys 10 (bL21)**	**Intra bL21Lys 10 (bL21)-Glu 23 (bL21)**	
4	**uL15-bL21**	SB	3-3	2-2	81	**Intra bL21Arg 83(bL21)-Tyr 81 (bL21)Arg 82(bL21)-Phe 75 (bL21)Intra uL15Lys 29 (uL15)-** His 35 (uL15)		**Inter**Pro 23 (uL15)-**Arg 82** (bL21)
5	**uL3-bL17**	LS	4-2	4-2	106	**InterLys 109 (uL3)-His 3 (bL17)Intra uL3Arg 111 (uL3)-Tyr 160 (uL3)**		
6	**bL17-uL22**	LG	6-4	3-4	132	**Intra uL22Arg 37(uL22)-Tyr 28 (uL22)**	**Intra bL17Arg 104 (bL17)-Asp107(bL17)**InterArg 37 (uL22)- Glu 102 (bL17)	
7	**uL3-uL14**	GG	5-3	4-2	143	**Intra uL3**Arg 13 **(uL3)-Phe 15 (uL3)**	**Intra uL14Asp 73 (uL14)–Arg 71 (uL14)**	InterPro 72 (uL14)-Arg 19 (uL3)
8	**uL3-uL13**	LL	4-4	4-3	150	**InterTyr 78 (uL13) - Lys 152 (uL3)Tyr 75 (uL13) - Arg 149 (uL3)Intra uL3Tyr 151 (uL3) - Arg 149 (uL3)**		**Inter**Pro 79 (uL13**)–Tyr 151 (uL3)**
9	**bL35-bL33**	LG	3-6	3-3	160	**Inter**Trp 34 (bL35)**–Lys 8 (bL33)Intra bL33Intra bL33Tyr 21 (bL33)–Lys 38 (bL33)**	**Intra bL35Glu 40 (bL35)–Lys 36 (bL35)Glu 24 (bL33)–Arg 6 (bL33)**	
10	**bL9-bL28**	G-G	9-5	5-0	210	**Inter**Tyr 71 (bL28)**–Arg 27(bL9)Intra bL28**His 66 (bL28)**–lys 10 (bL28)Intra bL9Phe 29 (bL9)–**Arg 33 (bL9)		**Inter**Pro 68 (bL28**)–Arg 27 (bL9)**
11	**uL16-bL27 4y4p: P-tRNA** (4v8i: tRNA free)	LS	9-10 (2-2)	9-9 (2-2)	272 (65)		**InterGlu 80 (uL16)-His 3 (bL27)Glu 80 (uL16)-Lys 5 (bL27)**	
12	**bL17-bL32**	LG	10-10	5-8	371	**InterTyr 52 (bL32)–Lys 99 (bL17)His 43 (bL32)–Lys 99 (bL17)Intra bL32Tyr 52 (bL32) - lys 40 (bL32)**Tyr 51 **(bL32)-**lys 56 (bL32)	**InterGlu 115 (bL17)-Arg 55 (bL32)**Glu 118 **(bL17)-Arg 55 (bL32)Intra L17Glu 115 (bL17) - Arg 96 (bL17)**	
13	**uL22-bL32**	GS	10-13	9-8	461	**InterHis 23 (bL32)–Arg 15 (uL22)Intra bL32His 23 (bL32)–Arg 20 (bL32)His 43 (bL32)–Tyr 52 (bL32)Intra uL22Tyr 38 (uL22)–**Arg 37 (uL22)	**Inter**Glu 48 (bL32)**–**Arg 37 (uL22)**Intra uL22Asp 22 (uL22)–Arg 25 (uL22**)	**InterTyr 38 (uL22)–**Pro 47 (bL32)
14	**uL15-uL4**	SG	11-15	9-7	456	**Intra L4Trp 34 (uL4)–**Arg 38 (uL4)Arg 38 (uL4) - Tyr 99 (uL4)Arg 95 (uL4**) - Tyr 97 (uL4)Arg 45 (uL4) - Tyr 97 (uL4)**	Intra L4Glu 120 (uL4)**–**Arg 117 (uL4)	**InterTrp 34 (uL4)–Pro 8 (uL15)**
15	**uL23-uL29**	GG	16-14	8-6	581	**InterPhe 47 (uL23)–**Arg 30 (uL29)Tyr 5 (uL23)**–**Arg 30 (uL29)**Intra L29**Arg 30 (uL29)- arom 33 (uL29)**Intra L23**Lys 78 (uL23)-**Trp 29 (uL23)**	**Inter**Asp 6 (uL23)**–Lys 29 (uL29)**	**InterPro 11 (uL23)–Phe 37 (uL29)Phe 47 (uL23)–Phe 37 (uL29)Phe 47 (uL23)–Arom 33 (uL29)**
16	**uL13-bL20**	LH	13-15	10-10	584	**InterTrp 42 (uL13)–Arg 64 (bL20)Tyr 4 (uL13)–Arg 64 (bL20)Tyr 4 (uL13)–**Arg 101 (bL20)**Intra uL13Trp 42 (uL13)–Lys 37 (uL13)Intra bL20Trp 61 (bL20)–Arg 64 (bL20)**	**Intra L20Asp 97 (bL20)–**Arg 101 (bL20)	**Intra L13Trp 42 (uL13)–Pro 44 (uL13)**
17	**uL3-bL19**	GG	22-20	16-10	802	**Intra uL3Phe 15 (uL3)–**Arg 13 (uL3)**Intra L19**Tyr 14 (bL19)**–**Arg 13 b(L19)	**Inter**Asp 18 (uL3)**–**Lys 33 (bL19)	**InterPhe 15 (uL3)–Pro 81 (bL19)**
18	**uL15-bL35**	SG	13-24	8-15	838	**InterHis 7 (bL35)–**Arg 50 (uL15)**Intra L15Phe 51 (uL15)–**Lys 46 (uL15)Intra bL35Phe 48 (bL35)**–**Lys 26 (bL35)	**Inter**Arg 57 (bL35)**–Glu 52 (uL15)Intra uL15**Arg 55 (uL15)**–Glu 52 (uL15)Intra bL35**Arg 57 (bL35)**–Glu 54 (bL35)**	**InterPro 63 (uL15)–Arg 30 (bL35)**
19	**bL20-bL21**	GGHβ	26-26	16-18	984	**InterTyr 12 (bL21)-Lys 93 (bL20)Intra bL20Lys 93 (bL20)-Trp 61 (bL20)Arg 92 (bL20)-Tyr 76 (bL20)Intra bL21Arg 13 (bL21)-Phe 2 (bL21)Tyr 12 (bL21)-Lys 10 (bL21)**	**InterArg 13 (bL21)-Glu 102 (bL20)**Lys 6 (bL21)-**Glu 89 (bL20)**Intra bL21**Lys 10 (bL21)-Glu 23 (bL21)**	
20	**bL25-uL16**	GG	46-49	2-29	1709	**InterTyr 9 (uL16)-Lys 198 (bL25)Intra uL16Tyr 93 (uL16)-Arg 10 (uL16)Tyr 9 (uL16)-Lys 8 (uL16)Phe 29 (uL16)-Arg 67 (uL16)**Arg 133 (uL16)**-Tyr 32 (uL16)Intra L25**Arg 72 (bL25)**-Tyr 29 (bL25)**Arg 72 (bL25)**-**Phe 89 (bL25)	InterAsp 138 (uL16)- Arg 81 (bL25)**Arg 51** (uL16) - Glu 186 (bL25)**Arg 51** (uL16) - Glu 48 (bL25)**Intra bL25His 85 (bL25)-Asp 87 (bL25)**	InterTyr 137 (uL16)- Pro 83 (bL25)

See also Figs 5 and 6 and Supplementary Fig. S5 and Table S12 that display sequence conservation. The residues written in bold are conserved. The underlined residues belong to the protein interfaces.

## References

[b1] Ramakrishnan V. Ribosome structure and the mechanism of translation. Cell 108, 557–572 (2002). 11909526 10.1016/s0092-8674(02)00619-0

[b2] Ban N., Nissen P., Hansen J., Moore P. B. & Steitz T. A. The complete atomic structure of the large ribosomal subunit at 2.4 Å resolution. Science 289, 905–920 (2000). 10937989 10.1126/science.289.5481.905

[b3] Wimberly *et al.* Structure of the 30S ribosomal subunit. Nature 407, 327–339 (2000). 11014182 10.1038/35030006

[b4] Harms *et al.* High-resolution structure of the large ribosomal subunit from a mesophilic eubacterium. Cell 107, 679–688 (2001). 11733066 10.1016/s0092-8674(01)00546-3

[b5] Peng Z. *et al.* A creature with a hundred waggly tails: intrinsically disordered proteins in the ribosome *Cell. Mol. Life Sci*. 71, 1477–1504 (2014). 23942625 10.1007/s00018-013-1446-6 PMC7079807

[b6] Melnikov *et al.* One core, two shells: bacterial and eukaryotic ribosomes. Nature Struct. Mol. Biol . 19, 560–567 (2012). 22664983 10.1038/nsmb.2313

[b7] Klinge S., Voigts-Hoffmann F., Leibundgut & Ban N. Atomic structures of the eukaryotic ribosome. Trends Biochem. Sci. 37, 189–198 (2012). 22436288 10.1016/j.tibs.2012.02.007

[b8] Brodersen D., Clemons W. M., Carter A. P., Wimberly B. T. & Ramakrishnan V. Crystal structure of the 30 S ribosomal subunit from Thermus thermophilus: Structure of the proteins and their interactions with 16 S RNA. J. Mol. Biol. 316, 725–768 (2002). 11866529 10.1006/jmbi.2001.5359

[b9] Klein D. J., Moore P. B. & Steitz T. A. The role of ribosomal proteins in the structure assembly, and the evolution of the large ribosomal subunit. J. Mol. Biol. 340, 141–177 (2004). 15184028 10.1016/j.jmb.2004.03.076

[b10] Timsit Y., Acosta Z., Allemand F., Chiaruttini C. & Springer M. *Int. J. Mol. Sci*. 10, 817–834 (2009). 19399222 10.3390/ijms10030817 PMC2672003

[b11] Timsit Y., Allemand F., Chiaruttini C. & Springer M. Coexistence of two protein folding states in the crystal structure of ribosomal protein L20. EMBO reports 7, 1013–1018 (2006). 16977336 10.1038/sj.embor.7400803 PMC1618378

[b12] Barabasi A.-L. & Oltvai Z. N. Network biology: understanding the cell’s functional organisation. Nature Rev. Gen. 5, 101–113 (2004). 10.1038/nrg1272 14735121

[b13] Alon U. Network motifs: theory and experimental approaches. Nature Rev. Gen . 8, 450–461 (2007). 10.1038/nrg2102 17510665

[b14] Janin J., Bahadur R. P. & Chakrabarti P. Protein-protein interaction and quaternary structure. Quart. Rev. Biophys. 41, 133–180 (2008). 10.1017/S0033583508004708 18812015

[b15] Yutin N., Puigbo P., Koonin E. & Wolf Y. Phylogenomic of prokaryotic ribosomal proteins Plos One 7, e36972 (2012). 22615861 10.1371/journal.pone.0036972 PMC3353972

[b16] Dougherty D. The cation-π interaction. Acc. Ch. Res . 46, 885–893 (2013). 10.1021/ar300265y PMC3957424 23214924

[b17] Zondlo N. J. Aromatic-Proline interactions: elecronically tunable CH/π interactions. Acc. Ch. Res . 46, 1039–1049 (2013). 10.1021/ar300087y PMC3780429 23148796

[b18] Lin X., Wang Y., Ahmadibeni Y., Parang K. & Sun G. Structural basis for domain-domain communication in a protein tyrosine kinase, the C-terminal Src kinase. J. Mol. Biol 357, 1263–1273 (2006). 16483606 10.1016/j.jmb.2006.01.046

[b19] Grodick M. A., Muren N. B. & Barton J. K. DNA charge transport within the cell. Biochemistry 54, 962–973 (2015). 25606780 10.1021/bi501520w PMC4587570

[b20] Malvankar N. S. *et al.* Structural basis for metallic-like conductivity in microbial nanowires. mBio 6(2), e00084–15. doi: 10.1128/mBio.00084-15 (2015). 25736881 PMC4453548

[b21] Chan Y.-L., Dresios J. & Wool I. G. A pathway for the transmission of allosteric signals in the ribosome through a network of RNA tertiary interactions. J. Mol. Biol. 355, 1014–1025 (2006). 16359709 10.1016/j.jmb.2005.11.037

[b22] Makarov G. I., Golovin A. V., Sumbatayan N. V. & Bogdanov A. A. Molecular dynamics investigation of a mechanism of allosteric signal transmission in ribosomes. *Biochemistry* (Moscow) 80, 1047–1056 (2015). 26547073 10.1134/S0006297915080106

[b23] Calidas D., Lyon H. & Culver G. M. The N-terminal extension of S12 influences small ribosomal subunit assembly in Escherichia coli. RNA 20, 1–10 (2014). 24442609 10.1261/rna.042432.113 PMC3923127

[b24] Meskauskas A. & Dinman J. D. A molecular clamp ensures allosteric coordination of peptidyltransfer and ligand binding to the ribosomal A-site. Nucleic Acids Res. 38, 7800–7813 (2010). 20660012 10.1093/nar/gkq641 PMC2995063

[b25] Rhodin M. H. J. & Dinman J. D. An extensive Network of information flow through the B1b/c intersubunit bridge of the yeast ribosome. Plos One 6, e20048 (2011). 21625514 10.1371/journal.pone.0020048 PMC3098278

[b26] Ferreon A. C. M., Ferreon J. C., Wright P. & Deniz A. A. Modulation of allostery by protein intrinsic disorder. Nature 498, 390–394 (2013). 23783631 10.1038/nature12294 PMC3718496

[b27] Nussinov R., Tsai C.-J. & Ma B. The underappreciated role of allostery in the cellular network. Annu. Rev. Biophys . 42, 169–89 (2013). 23451894 10.1146/annurev-biophys-083012-130257 PMC6407633

[b28] Schmeing T. M. & Ramakrishnan V. What recent ribosome structures have revealed about the mechanism of translation. Nature 461, 1234–1242 (2009). 19838167 10.1038/nature08403

[b29] Holtkamp W., Wintermeyer W. & Rodnina M. V. Synchronous tRNA movements during translocation on the ribosome are orchestrated by elongation factor G and GTP hydrolysis. Bioessays 36, 908–918 (2014). 25118068 10.1002/bies.201400076

[b30] Kumar V. *et al.* Structure of BipA in GTP form bound to the ratcheted ribosome. *Proc. Natl. Acad. Sci.* USA 112, 10944–10949 (2015). 26283392 10.1073/pnas.1513216112 PMC4568239

[b31] Varshney L., Chen B. L., Paniagua E., Hall D. H. & Chklovskii D. B. Structural properties of the *Caenorhabditis elegans* neuronal network. Plos Comp. Biol . 7, e1001066 (2011). 10.1371/journal.pcbi.1001066 PMC3033362 21304930

[b32] Wilson D. N. & Nierhaus K. H. Ribosomal proteins in the spotlight. Crit. Rev. Biochem. Mol. Biol. 40, 243–267 (2008). 16257826 10.1080/10409230500256523

[b33] Bray D. Protein molecules as computational elements in living cells. Nature 376, 307–310 (1995). 7630396 10.1038/376307a0

[b34] Berman J. *et al.* The Protein Data Bank. Nucleic Acids Res. 28, 235–242 (2000). 10592235 10.1093/nar/28.1.235 PMC102472

[b35] Ban N. *et al.* A new system for naming ribosomal proteins. Curr. Opin. Str. Biol . 24, 165–169 (2014). 10.1016/j.sbi.2014.01.002 PMC4358319 24524803

[b36] The PyMOL Molecular Graphics System, Version 1.7.4 Schrödinger, LLC.

[b37] Krzywinski M. *et al.* Circos: an Information Aesthetic for Comparative Genomics. Genome Res. 19, 1639–1645 (2009). 19541911 10.1101/gr.092759.109 PMC2752132

[b38] Troshin P. V., Procter J. B. & Barton G. J. Java bioinformatics analysis web services for multiple sequence alignment. Bioinformatics 27, 2001–2002 (2011). 21593132 10.1093/bioinformatics/btr304 PMC3129525

[b39] Adams P. D. *et al.* PHENIX: a comprehensive Python-based system for macromolecular structure solution. Acta Cryst . D66, 213–221 (2010). 10.1107/S0907444909052925 PMC2815670 20124702

[b40] Winn M. D. *et al.* Overview of the CCP4 suite and current developments. Acta. Cryst . D67, 235–242 (2011). 10.1107/S0907444910045749 PMC3069738 21460441

[b41] Tina K. G., Bhadra R. & Srinivasan N. PIC: Protein interaction calculator. Nucl. Acids Res . 35, W473–W476 (2007). 17584791 10.1093/nar/gkm423 PMC1933215

