# Supplementary material for: Neuron-Like Networks Between Ribosomal Proteins Within the Ribosome
Source: Sci Rep. 2016 May 26;6:26485. doi: 10.1038/srep26485 (PMC4881015; doi:10.1038/srep26485)
Supplement: Supplementary Information [file srep26485-s1.pdf]

Neuron-Like Networks Between Ribosomal Proteins Within the Ribosome

Olivier Poirot & Youri Timsit

Supplementary Information

Table S1

Properties of the extensions of the eukaryotic ribosomes (from the pdb structure 4v88)

Segment	Length (aas)	Mix.	Lenth (aas)	α−helix tot	α−helix prop	Loop	Length (aas)	Helix	Length (aas)	β-HP	Length (aas)

60S eukaryotes

Segment	Length (aas)	Mix.	Lenth (aas)	α−helix tot	α−helix prop	Loop	Length (aas)	Helix	Length (aas)	β-HP	Length (aas)
**uL3**	20	**uL3**	14	7	0,50	**uL3**	54	**eL14**	49	**uL22**	21
**eL6_1**	28	**uL4**	100	34	0,34	**uL4_1**	71	**uL13**	54		
**eL6_2**	16	**uL18_1**	29	11	0,38	**uL4_2**	19	**eL34**	33		
**eL14**	10	**uL18_2**	43	23	0,53	**uL16**	22	**eL40**	15		
**eL15**	32	**uL30**	60	43	0,72	**uL5_1**	18	**eL43_1**	32		
**eL18**	18	**eL8_1**	50	14	0,28	**uL5_2**	25	**eL43_2**	18		
**eL20**	28	**eL8_2**	25	13	0,52	**eL15**	31	**av**	33,5		
**eL21**	25	**uL16**	22	5	0,23	**uL13**	18	**max**	54		
**uL23**	37	**eL13_1**	47	18	0,38	**eL20**	14				
**eL33**	6	**eL13_2**	71	26	0,37	**uL14**	12				
**eL34**	37	**uL22**	32	12	0,38	**uL23**	6				
**eL42**	11	**eL18**	10	5	0,50	**eL27**	16				
**av.**	22,3	**eL19**	136	98	0,72	**eL32**	69				
**max**	37	**eL21**	60	23	0,38	**eL33_1**	12				
		**eL24**	39	6	0,15	**eL33_2**	10				
		**uL24**	22	8	0,36	**eL42**	42				
		**uL15**	70	7	0,10	**av**	27,4				
		**eL29**	57	29	0,51	**max**	71				
		**uL29**	50	16	0,32						
		**eL36**	47	14	0,30						
		**eL37**	86	26	0,30						
		**eL39**	49	12	0,24						
		**av.**	50,9	20,5	0,4						
		**max**	136								

40S eukaryotes

Segment	Length (aas)	Mix.	Lenth (aas)	α−helix tot	α−helix prop	Loop	Length (aas)	Helix	Length (aas)	β-HP	Length (aas)
**uS3**	30	**uS5**	25	9	0,36	**eS4**	14			**uS5**	25
**eS4**	35	**eS4**	19	9	0,47	**eS7**	19			**uS17**	27
**uS4**	18	**uS7**	61	9	0,15	**eS8**	27			**eS19**	10
**eS10**	16	**eS6**	108	59	0,55	**uS17**	43			**uS10**	21
**uS15**	26	**uS4**	20	17	0,85	**eS12**	20			**av**	20,8
**uS11**	20	**uS17**	14	4	0,29	**eS21**	13			**max**	27
**uS19_1**	13	**eS17**	55	20	0,36	**eS26**	25				
**uS19_2**	11	**uS13**	70	30	0,43	**av**	23				
**uS9**	30	**uS12**	38	29	0,76	**max**	43				
**uS13**	29	**eS24**	40	27	0,68						
**eS21_1**	17	**eS27**	30	7	0,23						
**eS21_2**	12	**uS14**	52	16	0,31						
**eS31**	24	**eS30**	59	20	0,34						
**av.**	21,6	**av.**	45,5	19,7	0,44						
**max**	35	**max**	108								

Table S2

Properties of the extensions of the eubacterial and archaeal ribosomes (from the pdb structure 4v8i and 1s72, respectively)

Segment	Length (aas)	Mix	Length (aas)	α−helix tot.	α−helix prop.	Loop	Length (aas)	Helix	Length (aas)	β-HP	size
**50S eubact.**											
**uL13**	11	**uL2**	52	8	0,15	**uL3**	53	**bL9**	37	**bL21**	17
**uL15**	78	**bL19**	19	9	0,47	**uL4**	59	**bL19**	13	**uL22**	16
**uL16**	30	**bL20**	71	54	0,76	**uL13_1**	16	**av.**	**25**	**uL23**	17
**bL17**	13	**bL25**	25	8	0,32	**uL13_2**	15		37	**bL28**	29
**bL27**	26	**bL32**	29	11	0,38	**uL16**	13			**av.**	**19,8**
**bL31_1**	9	**av.**	**39,2**		**0,42**	**bL17**	12			**max**	29
**bL31_2**	13	**max**	71			**bL35**	21				
**bL34**	6					**av.**	**27**				
**av.**	**23,3**					**max**	59				
**max**	78										
**30S eubact**											
**bS6**	8	**uS12**	27	8	0,30	**uS8**	19	**uS5**	8	**uS5**	13
**uS7**	18	**uS13**	50	29	0,58					**uS7**	10
**uS9**	29	**uS14**	59	21	0,36					**uS10**	24
**uS11**	16	**av.**	**45,3**		**0,41**					**uS17**	17
**bS18**	10	**max**	59							**av.**	**16**
**uS19**	9									**max**	24
**av.**	**15**										
**max**	29										
**50S archae**											
**uL3**	15	**eL19**	39	22	0,56	**uL3**	56	**uL24**	22	**eL22**	16
**uL15**	60	**eL37**	55	4	0,07	**uL4_1**	62	**eL43**	11		
**eL15**	24	**eL39**	48	18	0,38	**uL4_2**	12	av.	**16,5**		
**uL18**	19	**av.**	**47,3**		**0,34**	**uL5_1**	20	**max**	22		
**eL21**	25	**max**	55			**uL5_2**	11				
**av.**	**28,6**					**uL16**	22				
**max**	60					**uL13_1**	12				
						**uL13_2**	9				
						**uL14**	12				
						**eL15**	29				
						**eL32**	45				
						**eL42**	43				
						**av.**	**27,.8**				
						**max**	62				

Table S3

List of the intermolecular interactions between the ribosomal proteins within the 60S subunit of the eukaryotic ribosome (from the pdb structure 4v88). Colour code: light pink: 3 extensions; light blue: 2 extensions; light green: 1 extension; white: without extension.

**Name**	**Ch.**		**1**	**2**	**3**	**4**	**5**	**6**	**7**	**8**
**uL2**	A	glob. domain	eL8<br>***GS***	eL15<br>***GL***	eL43<br>***GG/GH***					
**uL3**	B	glob. dom.	uL13<br>***GG***	uL14<br>***GG***	eL24<br>***GG***	eS6 (40S)<br>***GG***				
		seg-N 1-21		uL14<br>***SL***						
		loop 220-274	uL13<br>***LL***							
		mix-C 373-387			eL24<br>***HG***					
**uL4**	C	glob. dom.						eL18<br>***GG***		
		loop 44-115	eL13<br>***LH***	eL15<br>***LS***	eL37<br>***LH***					
		loop 183-202				uL24<br>***LH***				
		mix-C 262-362					uL30<br>***HH/HG***	eL18<br>***HG***	eL20<br>***HG***	eL21<br>***HS***
**uL5**	J	glob. dom.								
		loop 48-66		eL42<br>***LS***						
		loop 131-156	uL18<br>***LS/LG***							
**uL6**	H	glob. dom.	eL14<br>***GS***	uL13<br>***GG***	eL20<br>***GS***	eL40<br>***GH***				
**eL6**	E	glob. dom.		eL14<br>***GH***	eL33<br>***GG/GS***					
		seg-N 2-30	eL32<br>***SG***							
		seg-C 160-176			eL33<br>***SS/SG***					
**eL8**	G	glob. dom.		eL15<br>***GG***			eL36<br>***GG***			
		mix-N 24-73	uL2<br>***SG***	eL15<br>***HG***	uL23<br>***SS***	eL27<br>***SL***				
		mix-C 231-256	-							
**uL13**	O	glob. dom.	uL3<br>***GG***	uL6<br>***GG***		eL20<br>***GS***	eL33<br>***GG***			
		loop 58-76	uL3<br>***LL***							
		helix 145-199			eL14<br>***HH***					
**eL13**	L	glob. dom.					uL29<br>***GS***	eL36<br>***GS***		
		mix-N 2-49	uL4<br>***HL***	eL15<br>***SS***	eL18<br>***SG***	uL15<br>***SH***				
		mix-C 123-194				uL15<br>***SG***		eL36<br>***HS***		
**uL14**	V	glob. dom.	uL3<br>***GG***	eL24<br>***GG***						
		loop 39-51	uL3<br>***LS***							
**eL14**	M	glob. dom.				eL20<br>***GG /GS***				
		seg-N 3-13		uL6<br>***SG***		eL20<br>***SS***				
		helix 89-138	eL6<br>***HG***		uL13<br>***HH***					
**uL15**	a	glob. dom.	eL13<br>***GS***	eL18<br>***GG***		eL36<br>***GS***				
		mix-N 2-72	eL13<br>***HS***	eL18<br>***SG***	eL32<br>***SL***		eL42<br>***SH***			
**eL15**	N	glob. dom.			eL8<br>***GG***		uL29<br>***GS***	eL36<br>***GH***		
		loop 67-98	uL2<br>***LG***						eL42<br>***LL***	
		seg-C 172-204		uL4<br>***SL***		eL13<br>***SS***				

**uL16**	I	glob. dom.		eL20<br>***GG***	eL21<br>***GS***						
		loop 99-121	-								
		mix-C 199-221	uL18<br>***HH***								
**uL18**	D	glob. dom.	uL5<br>***GL***	eL21<br>***GG***							
		mix-N 1-30	uL5<br>***SL***	eL21<br>***SS***							
		mix-C 254-297			uL16<br>***HH***						
**eL18**	Q	glob. dom. 1	uL4<br>***GH***			uL15<br>***GG***					
		seg-N 2-20		uL30<br>***SG***							
		mix 144-154									
		glob. dom. 2			eL13<br>***GS***	uL15<br>***GS***					
**eL19**	R	glob. dom.	-								
		mix 53-189	eS7<br>(40S)								
**eL20**	S	glob. dom.	uL4<br>***GH***	uL30<br>***GG***		eL14<br>***GG***		eL21<br>***GS***			
		loop 11-25						eL21<br>***LS***			
		seg-C 144-172			uL6<br>***SG***	eL14<br>***SG/SS***	uL13<br>***SG***				
**eL21**	T	glob. dom.		uL18<br>***GG***				eL29<br>***GS***			
		seg-N 2-27		uL18<br>***SS***							
		mix-C 100-160	uL4<br>***SH***		uL30<br>***SS***	uL16<br>***SG***	eL20<br>***SG***				
**uL22**	P	glob. dom.	-								
		β-hairpin 122-143	-								
		mix-C 152-184	eL33<br>***SL***								
**eL22**	U	glob. dom.	-								
**uL23**	X	glob. dom.		uL29<br>***GG***							
		seg-N 22-59	eL8<br>***SS***	uL29<br>***SS***							
		loop 114-120			eL39<br>***LH***						
**uL24**	Y	glob. dom.		eL39<br>***GL***							
		mix-N 2-24	uL4<br>***HL***								
**eL24**	W	glob. dom.	uL3<br>***GS***	uL14<br>***GG***							
		mix-C 59-98			eS6 (40S)<br>***HG***						
**eL27**	Z	glob. dom.		eL30<br>***GG***	eL34<br>***GH***						
		loop 51-67	eL8<br>***LS***								
**uL29**	h	glob. dom.			uL23<br>***GG***						
		mix-C 70-120	eL13<br>***SG***	eL15<br>***SG***	uL23<br>***SS***	eL37<br>***SH***					
**eL29**	b	mix-N 2-59	eL21<br>***SG***								
**uL30**	F	glob. dom.	uL4<br>***GH***	eL18<br>***GS***	eL20<br>***GG***						
		mix-N 23-83<br>(helix maj)	uL4<br>***HH***			eL21<br>***SS***					
**eL30**	c	glob. dom.	eL27<br>***GG***	eL34<br>***GH***	eL43<br>***GG***						
**eL31**	d	glob. dom.	-								
**eL32**	e	glob. dom.	eL6<br>***GS***								
		loop 3-72		uL15<br>***LS***	eL33<br>***LL***						

**eL33**	f	glob. dom.	eL6<br>***GG***	uL13<br>***GG***						
		seg-N 2-8	eL6<br>***SG***							
		loop 16-28				eL32<br>***LL***				
		loop 54-64			uL22<br>***LS***					
**eL34**	g	glob. dom.	-							
		seg-N 2-39			eL39<br>***SS***					
		helix 80-113	eL27<br>***HG***	eL30<br>***HG***						
**eL36**	i	glob. dom.	eL8<br>***GG***							
		mix-N 2-49		eL13<br>***SG/SH***	eL15<br>***HG***	uL15<br>***SG***				
**eL37**	j	mix-N 2-88	uL4<br>***HL***	uL29<br>***HS***	eL39<br>***SS***					
**eL38**	k	glob. dom.	-							
**eL39**	l	mix-N 2-51	uL23<br>***HL***	uL24<br>***LG***	eL34<br>***SS***	eL37<br>***SS***				
**eL40**	m	glob. dom.	-							
		helix 77-92	uL6<br>***HG***							
**eL41**	N	glob. dom.	-							
**eL42**	o	glob. dom.	-							
		loop 27-69		eL15<br>***LL***	uL15<br>***HS***					
		seg-C 95-106	uL5<br>***SL***							
**eL43**	p	glob. dom.	uL2<br>***GG***	eL30<br>***GG***						
		helix 2-34	uL2<br>***HG***							
		helix 74-92	uL2<br>***HG***							

Table S4

List of the intermolecular interactions between the ribosomal proteins within the 40S subunit of the eukaryotic ribosome (from the pdb structure 4v88). Colour code as in Table S3.

Name	Ch.		1	2	3	4	5	6	7	8
**eS1**	B	glob. dom.	uS11<br>***GG***	eS26<br>***GL***						
**uS2**	A	glob. dom.	uS5<br>***GG/GH***	eS17<br>***GS/GH***	eS21<br>***GG/GL***					
**uS3**	D	glob. dom.	uS5<br>***GG***	eS10<br>***GG/GS***		uS10<br>***GG***	uS14<br>***GH***		Stm1<br>***GH***	
		seg-C 195-225			eS17<br>***SG***			GBP<br>***SG***		
**uS4**	J	glob. dom.	uS5<br>***GG***		uS8<br>***GG***	eS24<br>***GG***	eS30<br>***GH***			
		seg-N 2-20		eS4<br>***SG***						
		mix-C 166-186	-							
**eS4**	E	glob. dom.	eS6<br>***GH***	uS4<br>***GS***		eS24<br>***GG***				
		seg-N 2-37	-							
		loop 197-211			uS17<br>***LG***					
		mix-C 242-261		uS4<br>***HG***						
**uS5**	C	glob. dom.	uS2<br>***GG***	uS3<br>***GG***	uS4<br>***GG***	eS21<br>***GG/GS***	uS8<br>***GG***	Stm1<br>***GS***		
		β-hairpin 80-105	-							
		mix-C 225-250	uS2<br>***HG***			eS21<br>***SS***				
**eS6**	G	glob. dom.		eS24<br>***GH***	eL24<br>(60S)	uL3<br>(60S)				
		mix-C 118-226	eS4<br>***HG***							
**uS7**	F	glob. dom.		eS25<br>***GG***	eS28<br>***GG***					
		mix-N 20-81	uS9<br>***HG/SG***							
**eS7**	H	glob. dom.	uS15<br>***GS***	uS8<br>***GG***						
		loop 94-113	-							
**uS8**	W	glob. dom.	uS5<br>***GG***	eS7<br>***GG***	uS4<br>***GG***	uS17<br>***GB***	uS15<br>***GS***	eS21<br>***GG***	uS12<br>***GS***	eS27<br>***GS***
**eS8**	I	glob. dom.	uS17<br>***GL***							
		loop 3-30	uS17<br>***LG***							
**uS9**	Q	glob. dom.	uS7<br>***GH/GS***	eS19<br>***GG***		GBP<br>***GG***				
		seg-C 113-143			uS10<br>***SB***					
**uS10**	U	glob. dom.	uS3<br>***GG***							
		β-hairpin 63-84		uS9<br>***BS***	uS14<br>***BH***					
**eS10**	K	glob. dom.	uS3<br>***GG***	eS12<br>***GG***	S29<br>***GS***	STM1<br>***GH***				
		seg-C 80-96	uS3<br>***SG***							
**uS11**	O	glob. dom.	eS1<br>***GG***	eS26<br>***GL***						
		seg-C 117-137		eS26<br>***SG***						
**uS12**	X	glob. dom.			eS30<br>***GS***	STM1<br>***GS***				
		mix 2-40	uS17<br>***HB***	uS8<br>***SG***						
**eS12**	M	glob. dom.	eS10<br>***GG***	eS31<br>***GS***						
		loop 101-121	-							

**uS13**	S	glob. dom.		eS19<br>***GG***						
		seg-N 2-31			eS25<br>***SG***	STM1<br>***SS***				
		mix-C 76-146	uS19<br>***HG/SS***							
**uS14**	d	mix 4-56	uS3<br>***HG***	eS10<br>***SG***	uS10<br>***HB***					
**uS15**	N	glob. dom.		uS17<br>***GH***		eS27<br>***GG***				
		seg-N 2-28	eS7<br>***SG***		uS8<br>***SG***	eS27<br>***SS***				
**uS17**	L	glob. dom.	eS4<br>***GL***	eS8<br>***GL***						
		loop 2-45		eS8<br>***LG***						
		β-hairpin 83-110				uS8<br>***BG***	uS12<br>***BH***			
		mix-C 142-156			uS15<br>***HG***					
**eS17**	R	glob. dom.		uS3<br>***GS***	GBP<br>***GG***					
		mix-C 71-126	uS2<br>***SG/HG***							
**uS19**	P	glob. dom.	uS13<br>***GH***							
		seg-N 8-21	uS13<br>***SS***							
		seg-C 120-131		STM1<br>***SS***						
**eS19**	T	glob. dom.	uS9<br>***GG***	uS13<br>***GG***						
		β-hairpin 83-93	-							
**eS21**	V	glob. dom.	uS2<br>***GG***	uS5<br>***GG***	uS8<br>***GG***					
		seg-N 1-18		uS5<br>***SG/SS***						
		loop 37-50	uS2<br>***LG***							
		seg-C 75-87				eS27<br>***SS***				
**eS24**	Y	glob. dom.	eS4<br>***GG***		uS4<br>***GG***					
		mix-C 95-135		eS6<br>***HG***						
**eS25**	Z	glob. dom.	uS7<br>***GG***	uS9	uS13<br>***GS***					
**eS26**	a	glob. dom.		uS11<br>***GS***						
		loop 42-67	eS1<br>***LG***	uS11<br>***LG***						
**eS27**	b	glob. dom.	uS15<br>***GG***							
		mix-N 2-32	uS15<br>***SS***	eS21<br>***SS***	uS8<br>***SG***					
**eS28**	c	glob. dom.	uS7<br>***GG***							
**eS30**	e	mix 2-61	uS4<br>***HG***	uS12<br>***SG***	STM1<br>***SH***					
**eS31**	f	glob. dom.	-							
		seg-C 83-107	eS12<br>***SG***							

Table S5

List of the intermolecular interactions between the ribosomal proteins within the 50S subunit of the archaeal ribosome (from the pdb structure 1s72). Colour code as in Table S3.

Name	Ch.		1	2	3
**uL2**	A	glob. dom.	eL43 ***GH***		
**uL3**	B	glob. dom.	uL13 ***GG***	uL14 ***GG***	eL24 ***GG***
		seg-N 1-16		uL14 ***SL***	
		loop 205-261	uL13 ***LL***		
**uL4**	C	glob. dom.	eL18 ***GG***		
		loop 42-104			eL37 ***LS***
		loop 174-186		uL24 ***LH***	
**uL5**	D	glob. dom.			
		loop 51-71	-		
		loop 135-146	uL18 ***LS/LG***		
**uL6**	E	glob. dom.	uL13 ***GL***		
**eL8**	F	glob. dom.	eL15 ***GG***		
**uL10**	G	glob. dom.	-		
**uL11**	I	glob. dom.	-		
**uL13**	J	glob. dom.	uL3 ***GG***		
		loop 63-75	uL3 ***LL***		
		loop 120-129		uL6 ***LG***	
**uL14**	K	glob. dom.	uL3 ***GG***	eL24 ***GG***	
		loop 34-46	uL3 ***LS***		
**uL15**	L	glob. dom.	eL18 ***GG***		
		seg-N 1-61		eL32 ***SL***	eL42 ***SL***
**eL15**	M	glob. dom.	eL8 ***GG***		
		loop 68-97		eL42 ***LL***	
		seg-C 170-194	-		
**uL16**	H	glob. dom.	-		
		loop 97-119	-		
**uL18**	N	glob. dom.		eL21 ***GG***	
		seg-N 1-20	uL5 ***SL***	eL21 ***SS***	
**eL18**	O	glob. dom.	uL4 ***GG***	uL15 ***GG***	
**eL19**	P	glob. dom.	-		
		mix-C 51-90	> 30-S ?		
**eL21**	Q	glob. dom.	uL18 ***GG***		
		seg-N 1-26	uL18 ***SS***		
**uL22**	R	glob. dom.	-		
		β-hairpin 122-138	-		
**uL23**	S	glob. dom.	uL29 ***GG***	eL39 ***GH***	
**uL24**	T	glob. dom.	-		
		helix 1-23	uL4 ***HL***		

**eL24**	U	glob. dom.	uL3<br>***GG***	uL14<br>***GG***	
**uL29**	V	glob. dom.	uL23<br>***GG***		
**uL30**	W	glob. dom.	-		
**eL31**	X	glob. dom.	-		
**eL32**	Y	glob. dom.	-		
		loop 124-169	uL15<br>***LS***		
**eL37**	1	mix 1-56	uL4<br>***SL***	eL39<br>***SS***	
**eL39**	2	Mix 1_49	uL23<br>***HG***	eL37<br>***SS***	
**eL42**	3	glob. dom.	-		
		loop 24-67	uL15<br>***LS***	eL15<br>***LL***	
**eL43**	Z	glob. dom.	-		
		helix 71-82	uL2<br>***HG***		

Table S6

List of the intermolecular interactions between the ribosomal proteins within the 50S subunit of the eubacterial ribosome (from the pdb structure 4v8i). Colour code as in Table S3.

**Name**	**Ch.**		**1**	**2**	**3**	**4**
**uL2**	D	glob. dom.	uL2 ***GS***	bS6 (30S) ***GG***		
		mix-C 220-272	uL2 ***SG***			
**uL3**	E	glob. dom.		uL14 ***GG***		bL19 ***GH***
		loop 109-162	uL13 ***LL***		bL17 ***LS***	
**uL4**	F	glob. dom.	uL15 ***GS***			
		loop 44-103		bL20 ***LS***		
**uL5**	G	glob. dom.	bL31 ***GG/GS***	uS13 (30S) ***GG***		
**uL6**	H	glob. dom.	-			
**bL9**	I	glob. dom.	bL28 ***GG***			
		helix 41-78				
**uL13**	N	glob. dom.	-			
		seg-N 1-12			bL21 ***SG***	
		loop 35-51		bL20 ***LH***		
		loop 71-86	uL3 ***LL***			
**uL14**	O	glob. dom.	uL3 ***GG***	bL19 ***GG***		
**uL15**	P	glob. dom.	-			
		seg-N 1-79	uL4 ***SG***	bL21 ***SB***	bL35 ***SG***	
**uL16**	Q	glob. dom.	bL25 ***GG***			
		seg-N 1-31	bL25 ***SG***			
		loop 76-89		bL27 ***LS***		
**bL17**	R	glob. dom.	-			
		seg-N 1-14	uL3 ***SL***			
		loop 99-111		uL22 ***LG***	bL32 ***LG***	
**uL18**	S	glob. dom.	bL27 ? ***GG***			
**bL19**	T	glob. dom.	uL3 ***GG***	uL14 ***GG***		
		helix 1-14	uL3 ***HG***			
		mix-C 116-135	-			
**bL20**	U	glob. dom.			bL21 ***GG***	
		mix-N 1-72	uL4 ***SL***	uL13 ***HL***	bL21 ***HB***	bL32 ***SH***
**bL21**	V	glob. dom.	uL13 ***GS***		bL20 ***GG***	
		β-hairpin 70-87		uL15 ***BS***	bL20 ***BH***	
**uL22**	W	glob. dom.	bL17 ***GL***	bL32 ***GS***		
		β-hairpin 82-98	-			
**uL23**	X	glob. dom.	uL29 ***GG***			
		β-hairpin 59-76		bL34 ? ***BS***		
**uL24**	Y	glob. dom.	-			

**bL25**	Z	glob. dom.	uL16<br>***GG***			
		mix-C 177-202	uL16<br>***SG***			
**bL27**	0	glob. dom.		uL18<br>***GG***		
		seg-N 2-28	uL16<br>***SL***			
**bL28**	1	glob. dom.	bL9<br>***GG***			
		β-hairpin 13-42	-			
**uL29**	2	glob. dom.	uL23<br>***GG***			
**uL30**	3	glob. dom.	-			
**bL31**	4	glob. dom.	uL5<br>***GG***	uS13 (30S)<br>***GG***		
		seg-N 1-10	uL5<br>***SG***			
		seg-C 33-46	uL5<br>***SG***			
**bL32**	5	glob. dom.	bL17<br>***GL***			
		mix-N 2-31		bL20<br>***HS***	uL22<br>***SG***	
**bL33**	6	glob. dom.	bL35<br>***GL***			
**bL34**	7	glob. dom.	-			
		seg-N 1-7	uL23<br>***SB***			
**bL35**	8	glob. dom.	uL15<br>***GS***			
		loop 25-46		bL33<br>***LG***		
**bL36**	9	glob. dom.	-			

Table S7

List of the intermolecular interactions between the ribosomal proteins within the 30S subunit of the eubacterial ribosome (from the pdb structure 4v8i). Colour code as in Table S3.

Name	Ch.		1	2	3	4
**uS2**	B	glob. dom.	uS8 ***GL***			
**uS3**	C	glob. dom.	uS5 ***GG***	uS10 ***GG/GB***	uS14 ***GH***	
**uS4**	D	glob. dom.	uS5 ***GG***			
**uS5**	E	glob. dom.	uS3 ***GG***	uS4 ***GG***		
		β-hairpin 15-28	-			
		helix 144-152			uS8 ***HG***	
**bS6**	F	glob. dom.	uS15 ***GG***	bS18 ***GG***	uL2 (50S) ***GG***	
		seg 92-100		bS18 ***SG***		
**uS7**	G	glob. dom.	uS9 ***GG***	uS11 ***GG***		
		seg-N 2-20	-			
		β-hairpin 77-87			Inh ***BH***	
**uS8**	H	glob. dom.		uS5 ***GH***	uS17 ***GB***	
		loop 63-82	uS2 ***LG***			
**uS9**	I	glob. dom.	uS7 ***GG***			
		seg-C 97-126		uS10 ***SB***	uS14 ***SS***	
**uS10**	J	glob. dom.	uS3 ***GG***			
		β-hairpin 43-67	uS3 ***BG***	uS9 ***BS***	uS14 ***BH***	
**uS11**	K	glob. dom.	uS7 ***GG***	bS18 ***GS***		
		seg-C 110-126	-			
**uS12**	L	glob. dom.	-			
		mix 5-32	uS17 ***HB***			
**uS13**	M	glob. dom.	-			
		mix-C 65-115	uS19 ***HG/HS***	uL5(50S) ***GG***	bL31(50S) ***GG***	
**uS14**	N	mix-N 2-61	uS3 ***HG***	uS9 ***SS***	uS10 ***HB***	
**uS15**	O	glob. dom.	bS6 ***GG***			
**bS16**	P	glob. dom.	-			
**uS17**	Q	glob. dom.	-			
		β-hairpin 23-40	uS8 ***BG***	uS12 ***BH***		
**bS18**	R	glob. dom.	bS6 ***GG/GS***			
		seg-C 77-87		uS11 ***SG***		
**uS19**	S	glob. dom.	uS13 ***GH***			
		seg-C 75-84	uS13 ***SH***			
**S20**	T	-				

Table S8

Numbers of interacting partners within the networks of ribosomal proteins. Proteins are listed in function of their number of interacting partners. Colour code as in Table S3.

**Number of interacting partners**	**Number and names of the proteins found in each category**												
**60S Euk.**	**1**	**2**	**3**	**4**	**5**	**6**	**7**	**8**	**9**	**10**	**11**	**12**	**13**
**0**	eL22	eL31	eL38	eL41									
**1**	uL22	eL19	eL29	eL40									
**2**	uL5	uL14	uL24	eL43									
**3**	uL2	uL18	eL6	uL16	eL24	uL23	eL27	eL30	eL32	eL34	eL37	eL42	
**4**	uL3	uL30	uL6	eL14	eL18	eL33	uL29	eL36	eL39				
**5**	eL8	uL13	uL15										
**6**	eL13	eL20	eL21										
**7**	eL15												
**8**	uL4												
**40S**													
**0**													
**1**	eS8	uS19	eS28	eS31									
**2**	eS1	eS7	eS12	uS11	eS17	eS19	eS26	eS30					
**3**	uS2	uS7	eS10	uS9	uS13	uS10	uS12	eS24	eS25	eS27	uS14		
**4**	eS4	eS6	uS15	eS21									
**5**	uS5	uS3	uS4	uS17									
**6**													
**7**													
**8**	uS8												
**50S eubact**													
**0**	uL6	uL24	uL30	bL36									
**1**	uL2	bL9	uL18	bL25	bL28	uL29	bL33	bL34					
**2**	uL4	uL5	uL14	uL16	bL19	uL22	uL23	bL27	bL31				
**3**	uL13	uL15	bL17	bL21	bL32								
**4**	uL3	bL20											
**30S**													
**0**	bS16	bS20											
**1**	uS2	uS4	uS12	uS15	uS19								
**2**	bS6	uS7	uS11	uS17	bS18								
**3**	uS3	uS5	uS8	uS9	uS10	uS13	uS14						
**50S archaea**													
**0**	uL10	uL16	uL11	uL22	uL30	eL31							
**1**	uL2	uL5	uL6	eL8	eL19	eL21	uL24	uL29	eL32	eL43			
**2**	uL13	uL14	eL15	uL18	eL18	eL24	eL37	eL39	eL42				
**3**	uL3	uL4	uL15										

Table S9
Properties of the ribosomal protein networks according to Barabasi et al. (2004) (reference 4 in Methods)

network	mean degree	avg clustering coef.	assortativity	gamma	beta	avg. shortest path	log(n)
60S euk.	3,66	0,31	0,18	2,52	0,25	3,3	3,56
40S euk.	2,81	0,31	0,15	2,64	1,05	3,54	3,26
50S eub.	2,62	0,36	-0,02	1,23	1,86	2,73	2,56
30S eub.	2,13	0,13	0,24	n/a	n/a	4,26	2,71
50S arc.	1,71	0	-0,16	n/a	n/a	2,66	2,64

Table S10
Motifs found in the protein ribosomal networks according to Alon et al. (2007) (reference 5 in Methods)

60S eukarya
size 3:
238 22 7.2+-2.2 6.67 0.000 7 125.71
size 4:
4958 123 53.0+-15.4 4.55 0.000 5 226.94
size 5:

40S eukarya
size 3:
238 14 3.6+-1.7 6.26 0.000 6 135.92

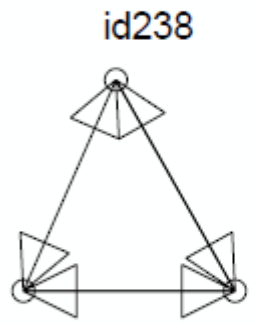

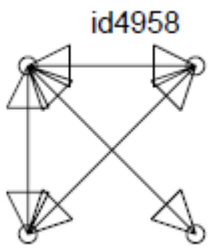

Table S11

List of the pdb structures of eubacterial 70S ribosomes and 50S subunits analysed.
A brief summary about each structure is indicated: year of publication, resolution, space group, translation step, occupancy of tRNA sites and mRNA, translation factor and antibiotics.

					tRNA					
**PDB_ID**	**Year**	**Resol.**	**Sp. Gr.**	**Functional state**	**A**	**P**	**E**	**mRNA**	**factor**	**Antibiotic**
T. Thermophilus										
**4V6F**	2010	3.1	$P2_12_12_1$	Elongation	phe	phe	phe	30		
**4V6G**	2010	3.5	$P2_12_12_1$	Initiation		met	met	25		
**4V87**	2012	3.1	$P2_12_12_1$	Elongation<br>decoding	leu	met	met	10		
**4V8B**	2012	3.0	$P2_12_12_1$	Elongation<br>decoding	leu<br>NC	met	met	10		
**4V8C**	2012	3.3	$P2_12_12_1$	Elongation<br>decoding	leu<br>NC	met	met	10		paromomycin
**4V8D**	2012	3.3	$P2_12_12_1$	Elongation<br>decoding	tyr	met	tyr	16		
**4V8E**	2012	3.3	$P2_12_12_1$	Elongation<br>decoding	tyr<br>NC	met	tyr	16		
**4V8F**	2012	3.3	$P2_12_12_1$	Elongation<br>decoding	tyr<br>NC	met	tyr	16		paromomycin
**4V9A**	2013	3.3	$P2_12_12_1$	Initiation		met		4		tetracyclin
**4V9B**	2013	3.1	$P2_12_12_1$	Initiation		met		6		tigecyclin
**4WQ1**	2015	3.1	$P2_12_12_1$	Elongation<br>decoding	tyr<br>NC	met	tyr	30		
**4WQR**	2015	3.1	$P2_12_12_1$	Elongation<br>decoding	phe<br>NC	met	phe	30		
**4WR6**	2015	3.05	$P2_12_12_1$	Elongation<br>decoding	tyr<br>NC	met	tyr	27		
**4WRA**	2015	3.05	$P2_12_12_1$	Elongation<br>decoding	tyr<br>NC	met	tyr	30		paromomycin
**4WRO**	2015	3.05	$P2_12_12_1$	Elongation<br>decoding	phe<br>NC	met	phe	30		
**4WSD**	2015	2.95	$P2_12_12_1$	Elongation<br>decoding	phe<br>NC	met	phe	30		paromomycin
**4WSM**	2015	3.3	$P2_12_12_1$	Elongation<br>decoding	leu<br>NC	leu	leu	60		
**4WT1**	2015	3.05	$P2_12_12_1$	Elongation<br>decoding	phe<br>NC	met	phe	27		
**4WU1**	2015	3.2	$P2_12_12_1$	Elongation<br>decoding		tyr<br>NC	tyr	27		
**4WZD**	2015	3.1	$P2_12_12_1$	Elongation<br>decoding		tyr	tyr	30		
**4WZO**	2015	3.3	$P2_12_12_1$	Elongation<br>decoding	met	met	phe	27		
**4V51**	2006	2.8	$P2_12_12_1$	Elongation<br>Pre1transloc	asl<br>phe	met	phe	24		paromomycin
**4V5A**	2007	3.5	$P2_12_12_1$	termination		asl<br>phe	met	31	RRF	
**4V5E**	2008	3.45	$P2_12_12_1$	Termination		phe	phe	8	RF2	
**4V5C**	2009	3. 3	$P2_12_12_1$	Elongation<br>Post1pept transfer	phe	met	phe	10		paromomycin
**4V5D**	2009	3.5	$P2_12_12_1$	Elongation<br>Pre1pept transf	phe	phe	phe	11		paromomycin
**4V7J**	2009	3.3	$P2_12_12_1$	Pre1mRNA<br>cleavage		met	met	25	relE	
**4V5J**	2010	3.1	$P2_12_12_1$	Termination		phe	phe	8	RF2	
**4V5L**	2010	3.1	$P12_11$	Elongation<br>decoding	trp	phe	phe	14	EF1Tu<br>GCP	paromomycin
**4V5P**	2011	3.1	$P12_11$	Elongation<br>decoding	trp<br>NC	phe	phe	27	EF1Tu<br>GDP	kirromycin
**4V5Q**	2011	3.1	$P12_11$	Elongation<br>decoding	trp<br>NC	phe	phe	27	EF1Tu<br>GDP	kirromycin
**4V5S**	2011	3.1	$P12_11$	Elongation<br>decoding	Trp<br>NC	phe	phe	27	EF1Tu<br>GDP	kirromycin
**4V5R**	2011	3.1	$P12_11$	Elongation<br>decoding	trp	phe	phe	27	EF1Tu<br>GDP	kirromycin
**4V8Q**	2012	3.1	$P12_11$	elongation	tm	met	met	19	EF1Tu<br>GDP	Kirromycin
**4V8N**	2013	3.1	$P2_12_12_1$	Elongation	ile	ile	ile	24		
**4V9H**	2013	2.86	$P12_11$	elongation<br>P/E intermediate		P/E<br>hyb		25	EF1G<br>GCP	
**4V9I**	2013	3.3	$P2_12_12_1$	elongation<br>decoding	ser	ser	ser	7		paromomycin
**4V63**	2008	3.2	$P2_12_12_1$	Termination		met	met	27	RF1	
**4V67**	2008	3.0	$P2_12_12_1$	Termination		met	met	27	RF2	
**4V83**	2011	3.5	$P2_12_12_1$			IRES				
**4V84**	2011	3.4	$P2_12_12_1$			IRES				
**4V9N**	2013	3.4	$P2_12_12_1$	termination		met		10	RF2	
**4V9Q**	2013	3.4	$P2_12_12_1$	Translation inhibition		met	met	5		Blasticidin
**4V90**	2013	2.95	$P12_11$	Pre1translocation		P/E<br>hyb			EF1G<br>GCP	
**4V8X**	2013	3.35	$P2_12_12_1$	Pre1 mRNA cleavage		met	met	25	YOEB	
**4V9K**	2013	3.5	C121	Intermdediate state<br>of translocation		P/E<br>hyb		23	EF1G<br>GDPNP	viomycin

**4V9L**	2013	3.5	$P2_12_12_1$	Pe*/E state		P/E hyb		23	EF1G GDP	Fusidic acid
**1VVJ**	2014	3.44	$P2_12_12_1$	+1 frame shift decoding	ASL SufA6	met		18		paromomycin
**4L47**	2014	3.22	$P2_12_12_1$	+1 frame shift decoding	ASL SufA6	met		18		paromomycin
**4LNT**	2014	2.94	$P2_12_12_1$	+1 frame shift decoding	ASL SufA6	met		18		paromomycin
**4LSK**	2014	3.48	$P2_12_12_1$	+1 frame shift decoding	ASL SufA6	met		17		
**4TUE**	2014	3.5		+1 frame shift decoding	ASL SufJ	met		25		Paromomycin PPU
**4ZER**	2015	3.1	$P2_12_12_1$	Translation inhibition		met		27	Onc1112	
**4V6A**	2009	3.1	$P2_12_12_1$	1st peptide bond		met		5	EF1P	
**4V7L**	2010	3.0	$P2_12_12_1$	Translocation Inhibition	gln	met	gln	30		viomycin
**4V7W**	2010	3.0	$P2_12_12_1$							chloramphenicol
**4V7X**	2010	3.0	$P2_12_12_1$							erythromycin
**4V7Y**	2010	3.0	$P2_12_12_1$							azythromycin
**4V7Z**	2010	3.1	$P2_12_12_1$							telithromycin
**4V8A**	2012	3.2	$P2_12_12_1$							thermorubin
**4V8G**	2012	3.0	$P2_12_12_1$	stationary					RMF	
**4V8H**	2012	3.1	$P2_12_12_1$	stationary					HPF	
**4V8I**	2012	2.7	$P2_12_12_1$	stationary					YfiA	
**4V95**	2012	3.2	$P2_12_12_1$	stationary		met		16	YaeJ	
**1VY4**	2014	2.6	$P2_12_12_1$	Pre1attack	phe	met	phe	24		
**1VY5**	2014	2.55	$P2_12_12_1$	Post1catalysis	phe	met	phe	24		
**1VY6**	2014	2.9	$P2_12_12_1$	Pre1attack	Mimic	met		24		
**1VY7**	2014	2.8	$P2_12_12_1$	Pre1attack	Mimic	met		24		
**4V9R**	2014	3.0	$P2_12_12_1$			met		24		dityromycin
**4V9S**	2014	3.1	$P2_12_12_1$			met		24		GE82832
**4W2E**	2014	2.9	$P2_12_12_1$	Back translocation		phe	phe	18	EF14 GDP	
**4W2F**	2014	2.4	$P2_12_12_1$		phe	met	phe	24		amicoumacin
**4W2G**	2014	2.55	$P2_12_12_1$		phe	met	phe	24		pactamycin
**4W2H**	2014	2.7	$P2_12_12_1$			met	Phe aS	24		pactamycin
**4W2I**	2014	2.7	$P2_12_12_1$		phe	met	phe	24		negamycin
**4WPO**	2015	2.8	$P2_12_12_1$	Pre-translocation	phe	met	phe	24	EF1G GDP	
**4WQF**	2015	2.8	$P2_12_12_1$	Post-translocation		met	phe	18	EF1G GDP	Fusidic acid
**4WQU**	2015	2.8	$P2_12_12_1$	Post-translocation		phe	phe	18	EF1G GDP	ditromycin
**4Y4O**	2015	2.3	$P2_12_12_1$						YfiA	
**4Y4P**	2015	2.5	$P2_12_12_1$		phe	met	phe	24		
**4Z3Q**	2015	2.6	$P2_12_12_1$		phe	met	phe	24		hygromycin
**4Z3R**	2015	3.1	$P2_12_12_1$							hygromycin
**4Z3S**	2015	2.6	$P2_12_12_1$		phe	met	phe	27		4M2
**4Z8C**	2015	2.5	$P2_12_12_1$			met		24		oncocin
D. radiodurans										
**2ZJR**	2008	2.9	I222	50S						
**2ZJQ**	2008	3.3	I222	50S						micrococin
**3CF5**	2008	3.3	I222	50S						thiostrepton
**3PIO**	2011	3.25	I222	50S						Lankamycin lankacidin
**4IO9**	2013	3.2	I222	50S						erythromycin
**4IOA**	2013	3.2	I222	50S						4e
**5DM6**	2015	2.9	I222	50S						
E. coli										
**4V52**	2007	3.2	$P2_12_12_1$							neomycin
**4V54**	2007	3.3	$P2_12_12_1$	termination					RRF	
**4V57**	2007	3.5	$P2_12_12_1$							neomycin spectinomycin
**4V64**	2008	3.5	$P2_12_12_1$							Hygromycin B
**4V6C**	2009	3.19	$P2_12_12_1$	intermediate ratcheting						
**4V7U**	2010	3.1	$P2_12_12_1$							erythromycin
**4V7S**	2010	3.25	$P2_12_12_1$							telithromycin
**4V7V**	2010	3.29	$P2_12_12_1$							clindamycin
**4V7T**	2010	3.19	$P2_12_12_1$							chloramphenicol
**4WWW**	2010	3.1	$P2_12_12_1$							CEM1101
**4V9D**	2011	3.0	$P2_12_12_1$	termination		phe		16	RRF	
**4V85**	2012	3.2	$P2_12_12_1$	termination				27	RF3 GDPNP	viomycin
**4V9C**	2012	3.3	$P2_12_12_1$	termination		phe		24	RRF	neomycin
**4V9O**	2013	2.9	$P12_11$	Subunit rotation					EF1G GCP	viomycin
**4U1U**	2014	2.95	$P2_12_12_1$							quinupristin
**4U1V**	2014	3.0	$P2_12_12_1$							linopristin
**4U20**	2014	2.9	$P2_12_12_1$							flopristin
**4U24**	2014	2.9	$P2_12_12_1$							dalfopristin
**4U25**	2014	2.9	$P2_12_12_1$							virginamycin
**4U26**	2014	2.8	$P2_12_12_1$							dalfopristin quinupristin
**4U27**	2014	2.8	$P2_12_12_1$							Flopristin linopristin
**4WF1**	2014	3.1	$P2_12_12_1$							negamycin
**4WOI**	2015	3.0	$P2_12_12_1$	termination		phe		16	RRF	paromycin
**4YBB**	2015	2.1	$P2_12_12_1$							

Table S12

Conserved residues at the interfaces of the interacting proteins within the 50S eubacterial subunit. Left column: residues found in the interface. Right column: conserved interface residues. Residues whose properties conserved are underlined and strictly conserved residues are written in bold. AR: conserved aromatic residues; BA: conserved basic residues; AC: conserved acid residues; HP: conserved hydrophobic residues; PO: conserved polar residues; SM: conserved small chain residues. See also figure uS5 that displays the consensus sequence from sequence alignments (reference 6 in Methods).

	**Interface residues**						**Conserved residues**			
**bL20-uL4**										
**bL20**	pro						pro/ala			
	2						2			
**uL4**	**arg**	ala	pro				**arg**	pro/ala		
	45	49	92				45	92		
**bL32-bL20**										
**bL32**	**lys**	**ser**	**lys**	ala			**BA**	**ser**	**BA**	SM
	10	12	13	14			10	12	13	14
**bL20**	trp	**gly**	leu	lys			AR/arg	BA/asn		
	25	26	27	30			25	30		
**uL13-bL21**										
**uL13**	**met**						**met**			
	1						1			
**bL21**	**gln**	**tyr**	**arg**	**leu**			**gln**	**AR**	**BA**	**HP**
	11	12	13	20			11	12	13	20
**uL15-bL21**										
**uL15**	pro	**gly**	**lys**				**gly**	**lys**		
	23	24	29				24	29		
**bL21**	gln	**arg**	**arg**				PO	**BA**	**BA**	
	80	82	83				80	82	83	
**uL3-bL17**										
**uL3**	**lys**	**gly**	**arg**	**lys**			**lys**	**gly**	**BA**	**lys**
	109	110	111	118			109	110	111	118
**bL17**	**met**	**his**					met	**his**		
	1	3					1	3		
**bL17-uL22**										
**bL17**	ala	glu	arg	**arg**	arg	**gly**	**gly**	**arg**	**gly**	
	101	102	103	104	105	106	101	104	106	
**uL22**	arg	**tyr**	asn	**arg**			PO	**AR**	PO	**BA**
	37	38	40	42			37	38	40	42
**uL3-uL14**										
**uL3**	arg	**phe**	asp	arg	ala		arg/gln	**AR**	AC/PO	PO
	13	15	18	19	20		13	15	18	19
**uL14**	pro	**asp**	**gly**				**asp**	**gly**		
	72	73	74				73	74		
**uL3-uL13**										
**uL13**	**tyr**	gly	**tyr**	pro			**AR**	**AR**	*pro*/HP	
	75	77	78	79			75	78	79	
**uL3**	**arg**	**tyr**	**lys**	**gly**			**BA**	**AR**	**lys**	**gly**
	149	151	152	153			149	151	152	153

**bL35-bL33**														
**bL35**	leu	trp	gln					HP	PO	PO				
	32	34	35					32	34	35				
**bL33**	ala	lys	ala	**thr**	glu	**ile**		**thre**	PO	**HP**				
	2	8	22	23	24	54		23	24	54				
**bL28-bL9**														
**bL28**	ile	pro	tyr	glu	glu									
	67	68	71	72	75									
**bL9**	**met**	**arg**	**asn**	**leu**	val	leu	**thr**	**arg**	**asn**	**leu**	**thr**			
	1	27	28	31	37	38	40	27	28	31	40			
	ser	asn						asn/HP						
	42	43						43						
**uL16-bL27**														
**uL16**	**lys**	**leu**	**glu**	val	**arg**	**gly**	lys	**lys**	**HP**	**glu**	val/thr	**arg**	**gly**	Lys /ser
	11	79	80	81	82	84	85	11	79	80	81	82	84	85
	**gly**	**lys**						**gly**	**lys**					
	86	87						86	87					
**bL27**	**ala**	**his**	**lys**	**lys**	gly	leu	**gly**	**ala**	**his**	**lys**	**lys**	gly/ala	**gly**	
	2	3	4	5	6	7	8	2	3	4	5	6	8	
	**ser**	thr	**asn**					**ser**	thr/ser	**asn**				
	9	10	11					9	10	11				
**bL17-bL32**														
**bL17**	arg	**arg**	**leu**	**lys**	leu	ala	gln	**arg**	**HP**	**lys**	SM			
	33	96	98	99	100	101	102	96	98	99	101			
	leu	**glu**	gln					**glu**						
	113	115	118					**115**						
**bL32**	pro	**his**	thr	**val**	pro	glu	**tyr**	**his**	**BA**	**HP**	PO	**tyr**		
	41	43	44	45	47	48	52	43	44	45	48	52		
	**arg**	**val**	**leu**					**val**	**HP**					
	55	57	58					57	58					
**uL22-bL32**														
**uL22**	arg	**leu**	**asp**	leu	asn	**ile**	arg	arg/gln	**leu**	**asp**	HP /gln	**HP**	PO	
	15	19	22	23	34	35	37	15	19	22	23	35	37	
	**tyr**	thr	lys					**AR**	thr/ser	lys/PO				
	38	39	41					38	39	41				
**bL32**	**arg**	his	**ala**	**leu**	thr	pro	pro	**arg**	AR/asp	ala/lys	T/K/S	pro/ HP		
	20	23	24	25	26	27	28	20	23	24	26	28		
	**leu**	met	**lys**	pro	pro	glu		**HP**	BA	PO				
	30	39	40	41	47	48		30	40	48				
**uL15-uL4**														
**uL15**	**met**	lys	**leu**	asp	**leu**	arg	**pro**	**met**	PO	**leu**	PO	**leu**	PO	**pro**
	1	2	3	5	6	7	8	1		3	5	6	7	8
	asn	**ala**	asn	arg				SM	PO					
	9	12	13	16				12	16					
**uL4**	ile	asn	pro	his	**leu**	**trp**	**val**	asn/PO	**HP**	**AR**	**val**			
	28	29	30	31	33	34	37	29	33	34	37			
	arg	arg	asp	arg	glu	tyr	val	PO	PO	PO				
	38	95	116	117	120	184	187	95	116	120				
	arg													
	188													

**uL23-uL29**															
**uL23**	met	thr	tyr	asp	**ile**	leu	ala	**pro**	PO	**ile**	**pro**				
	1	3	5	6	8	9	10	11	6	8	11				
	**leu**	**phe**	ala	**phe**	lys	ala	leu	leu	**HP**	**phe**	HP	HP	HP		
	13	28	46	47	48	91	92	95	13	28	46	92	95		
**uL29**	glu	**val**	arg	**lys**	arg	leu	met	**arg**	PO	**HP**	lys/leu	PO	**AR**	**arg**	
	22	25	26	29	30	32	33	36	22	25	29	30	33	36	
	**phe**	ser	ile	leu	leu	arg			**HP**						
	37	40	41	61	64	68			37						
**uL13-bL20**															
**uL13**	met	lys	**thr**	**tyr**	val				*lys/thr*	**thr**	**AR**				
	1	2	3	4	5				2	3	4				
	**lys**	his	**arg**	pro	asp	**trp**	**thr**	**pro**	**lys**	*his/asn*	*pro/ala*	**BA**	**AR**	**thr**	**pro**
	37	38	39	40	41	42	43	44	37	38	40	39	42	43	44
**bL20**	**phe**	**leu**	**trp**	val	**arg**	**ala**	ala	gln	**phe**	**leu**	**trp**	**arg**	**ala**	**ala**	
	57	60	61	63	64	67	68	71	57	60	61	64	67	68	
	**lys**	asn	ala	**asp**	**ala**	**val**	arg		**lys**	**AC**	**ala**	**HP**			
	93	94	96	97	99	100	101		93	97	99	100			
**uL3-bL19**															
**uL3**	val	**lys**	**val**	**gly**	**met**	**thr**	arg	**ile**	**lys**	**HP**	**gly**	**met**	**thr**	arg/gln	**HP**
	7	8	9	10	11	12	13	14	8	9	10	11	12	13	14
	**phe**	asp	asp	arg	**val**	leu	leu	asp	**AR**	PO	PO	**HP**	PO		
	15	17	18	19	25	27	52	174	15	17	18	25	174		
	ile	glu	**leu**	**leu**	asn	gly			**HP**	**HP**	PO	gly/PO			
	176	179	181	183	192	193			181	183	192	193			
**bL19**	met	arg	**leu**	**ile**	leu	**val**	gln	arg	**HP**	**ile**	**HP**	glu/PO	PO		
	1	3	6	7	9	10	11	13	6	7	10	11	13		
	tyr	**lys**	lys	thr	**phe**	asn	thr	pro	**BA**/AR	PO	**HP**	PO	SM		
	14	33	35	40	57	58	60	77	33	40	57	58	60		
	**his**	**ser**	**pro**	leu					**AR**/PO	**ser/thr**	**pro**				
	79	80	81	82					79	80	81				
**uL15-bL35**															
**uL15**	pro	arg	arg	**phe**	**glu**	arg	**thr**	leu	**gly**	**phe**	**glu**	**gln**			
	48	49	50	51	52	55	58	59	50	51	52	55			
	met	**arg**	**leu**	**pro**	**lys**	**arg**	gln		**arg**	**leu**	**pro**	**lys**			
	60	61	62	63	64	65	68		61	62	63	64			
**bL35**	**met**	**lys**	**his**	gly	ala	lys	**lys**	**arg**	**HP**	**lys**	**BA**	SM	SM	**lys**	**arg**
	4	5	7	9	10	11	12	13	4	5	7	9	10	12	13
	val	ala	met	lys	thr	arg	lys	phe	**phe**	PO	SM	arg/ser			
	14	24	25	26	27	30	47	48	14	26	27	30			
	**leu**	**glu**	arg	**ile**	leu	**leu**			**HP**	**glu**	**HP**	**HP**			
	50	54	57	58	60	61			50	54	58	61			

**bL20-bL21**															
**bL20**	**lys**	leu	arg	**leu**	phe	gly	**ala**	**tyr**	**lys**	leu/gln	arg/asn	HP	**ala**	**AR**	
	16	20	36	39	40	43	46	47	16	20	36	39	46	47	
	**arg**	**ala**	gly	**ile**	**glu**	**val**	asp	**lys**	**arg**	**ala**	**HP**	**AC**	**HP**	**lys**	
	50	86	87	88	89	90	91	93	50	86	88	89	90	93	
	asn	**asp**	**leu**	arg	**glu**	gln	val	glu	**AC**	**HP**	**AC**				
	94	97	98	101	102	104	105	108	97	98	102				
	**leu**	arg							**HP**						
	109	112							109						
**bL21**	**phe**	**ile**	lys	**gly**	**lys**	**gln**	**tyr**	**arg**	**AR**	**HP**	PO	**gly**	**lys**	**gln**	**AR**
	2	4	6	9	10	11	12	13	2	4	6	9	10	11	12
	**leu**	**leu**	**leu**	gly	gly	thr	**val**	**gly**	**HP**	**leu**	**HP**	**HP**	**gly**		
	38	39	40	41	42	45	47	48	38	39	40	47	48		
	thr	**pro**	**ile**	leu	**val**	ser	**lys**	phe	**pro**	**HP**	**HP**	**lys**			
	49	50	70	71	72	73	74	75	50	70	72	74			
	**arg**	**lys**							**BA**	**BA**					
	82	84							82	84					
**bL25-uL16**															
**bL25**	leu	phe	asp	phe	arg	ser	ile	his							
	41	44	45	48	49	52	53	54							
	arg	**gln**	val	leu	asp	lys	arg	arg	**gln**	BA/thr					
	72	73	74	76	77	78	79	80	73	78					
	arg	pro	ala	gly	val	leu	gln	phe							
	81	83	113	114	116	117	118	119							
	arg	asp	ile	val	pro	pro	glu	asp							
	122	123	146	175	176	178	179	180							
	val	lys	leu	glu	ala	ala	glu	val							
	181	182	183	186	187	189	190	191							
	ala	glu	pro	glu	val	lys									
	192	193	194	195	196	198									
**uL16**	**met**	met	**pro**	**arg**	**arg**	met	**lys**	**tyr**	**met**	**pro**	**BA**	**arg**	**lys**	**AR**	**AR**
	1	3	4	5	6	7	8	9	1	4	5	6	8	9	9
	**arg**	**gly**	ala	thr	lys	gly	asp	tyr	**arg**	**gly**					
	10	19	20	21	22	23	25	26	10	19					
	**val**	**phe**	**asp**	val	**glu**	**arg**	val	val	**HP**	**phe**	**AC**	**glu**	**arg**	thr/asn	thr/asn
	27	29	31	35	48	51	52	55	27	29	31	48	51	55	55
	**arg**	phe	**arg**	**arg**	gly	**gly**	lys	**ile**	**arg**	AR	**lys**	**arg**	gly/gln	**gly**	
	56	58	59	60	61	62	63	64	56	58	59	60	61	62	
	**phe**	**tyr**	**pro**	**gly**	ala	**gly**	lys	**val**	**AR**	**tyr**	**AR**	**pro**	**gly**	**HP**	
	65	93	99	100	107	108	130	132	65	93	93	99	100	132	
	arg	**arg**	asp	ala	tyr	asp	gln	ala	**BA**						
	133	134	135	136	137	138	139	140	134						
	glu														
	141														

Table S13

New nomenclature for proteins of the small subunit (adapted from reference 35)

New Name	Taxonomic Range	Bacteria	Yeast	Human
**bS1**	B	S1	-	-
**eS1**	A E	-	S1	S3A
**uS2**	B A E	S2	S0	SA
**uS3**	B A E	S3	S3	S3
**uS4**	B A E	S4	S9	S9
**eS4**	A E	-	S4	S4
**uS5**	B A E	S5	S2	S2
**bS6**	B	S6	-	-
**eS6**	A E	-	S6	S6
**uS7**	B A E	S7	S5	S5
**eS7**	E	-	S7	S7
**uS8**	B A E	S8	S22	S15A
**eS8**	A E	-	S8	S8
**uS9**	B A E	S9	S16	S16
**uS10**	B A E	S10	S20	S20
**eS10**	E	-	S10	S10
**uS11**	B A E	S11	S14	S14
**uS12**	B A E	S12	S23	S23
**eS12**	E	-	S12	S12
**uS13**	B A E	S13	S18	S18
**uS14**	B A E	S14	S29	S29
**uS15**	B A E	S15	S13	S13
**bS16**	B	S16	-	-
**uS17**	B A E	S17	S11	S11
**eS17**	A E	-	S17	S17
**bS18**	B	S18	-	-
**uS19**	B A E	S19	S15	S15
**eS19**	A E	-	S19	S19
**bS20**	B	S20	-	-
**bS21**	B	S21	-	-
**bTHX**	B	THX	-	-
**eS21**	E	-	S21	S21
**eS24**	A E	-	S24	S24
**eS25**	A E	-	S25	S25
**eS26**	E	-	S26	S26
**eS27**	A E	-	S27	S27
**eS28**	A E	-	S28	S28
**eS30**	A E	-	S30	S30
**eS31**	A E	-	S31	S27A
**RACK1**	E	-	Asc1	RACK1

Table S14

New nomenclature for proteins of the large subunit (adapted from reference 35)

New name	Taxonomic range	Bacteria	Yeast	Human
**uL1**	B A E	L1	L1	L10A
**uL2**	B A E	L2	L2	L8
**uL3**	B A E	L3	L3	L3
**uL4**	B A E	L4	L4	L4
**uL5**	B A E	L5	L11	L11
**uL6**	B A E	L6	L9	L9
**eL6**	E	-	L6	L6
**eL8**	A E	-	L8	L7A
**bL9**	B	L9	-	-
**ul10**	B A E	L10	P0	P0
**uL11**	B A E	L11	L12	L12
**bL12**	B	L7/L12	-	-
**uL13**	B A E	L13	L16	L13A
**eL13**	A E	-	L13	L13
**uL14**	B A E	L14	L23	L23
**eL14**	A E	-	L14	L14
**uL15**	B A E	L15	L28	L27A
**eL15**	A E	-	L15	L15
**uL16**	B A E	L16	L10	L10
**bL17**	B	L17	-	-
**uL18**	B A E	L18	L5	L5
**eL18**	A E	-	L18	L18
**bL19**	B	L19	-	-
**eL19**	A E	-	L19	L19
**bL20**	B	L20	-	-
**eL20**	A E	-	L20	L18A
**bL21**	B	L21	-	-
**eL21**	A E	-	L21	L21
**uL22**	B A E	L22	L17	L17
**eL22**	E	-	L22	L22
**uL23**	B A E	L23	L25	L23A
**uL24**	B A E	L24	L26	L26
**eL24**	A E	-	L24	L24
**bL25**	B	L25	-	-
**bL27**	B	L27	-	-
**eL27**	E	-	L27	L27
**bL28**	B	L28	-	-
**eL28**	E	-	-	L28
**uL29**	B A E	L29	L35	L35
**eL29**	E	-	L29	L29
**uL30**	B A E	L30	L7	L7
**eL30**	A E	-	L30	L30
**bL31**	B	L31	-	-
**eL31**	A E	-	L31	L31
**bL32**	B	L32	-	-
**eL32**	A E	-	L32	L32
**bL33**	B	L33	-	-
**eL33**	A E	-	L33	L35A
**bL34**	B	L34	-	-
**eL34**	A E	-	L34	L34
**bL35**	B	L35	-	-
**bL36**	B	L36	-	-
**eL36**	E	-	L36	L36
**eL37**	A E	-	L37	L37
**eL38**	A E	-	L38	L38
**eL39**	A E	-	L39	L39
**eL40**	A E	-	L40	L40
**eL41**	A E	-	L41	L41
**eL42**	A E	-	L42	L36A
**eL43**	A E	-	L43	L37A
**P1/P2**	A E	-	P1/P2 (A/B)	P1/P2 (α/β)

Table S15

Status of the pdb models when different conformations are observed at protein-protein interfaces of 50S subunits and 70S eubacterial ribosomes.

For each protein-pairs where different conformations have been observed, the status of the models is noted as follow: white: correct model; “?” and green: the map is not incompatible with the different model built in the map; “-” and yellow: non-interpretable electron density map in the corresponding region; “x” and red: incorrect model, the model is not correctly fitted into its density map. For uL16-bL27, the “S” indicates that bL27 is fully structured at the interface (see also Supplementary fig. S10 that display the correct and incorrect (x) model found in the twelve protein-protein contact regions that display different conformation

				tRNA			1	2	3	4	5	6	7	8	9	10	11	12
PDB_ID	Year	Res.	Sp. Gr	A	P	E	uL3 uL13	uL13 bL20	uL13 bL21	uL15 bL21	uL15 bL35	bL35 bL33	bL17 uL3	uL3 uL14	bL17 bL32	bL20 uL4	uL16 bL27	uL23 uL29
**T.Thermophilus**																		
**4V6F_1**	2010	3.1	$P2_12_12_1$	phe	phe	phe				?		-			x		x	x
**4V6F_2**											x	-			x		x	x
**4V6G_1**	2010	3.5	$P2_12_12_1$		met	met				?		-			x		x	x
**4V6G_2**											x	-			x		x	x
**4V87_1**	2012	3.1	$P2_12_12_1$	leu	met	met					x	-			x			x
**4V87_2**										?		-			x			x
**4V8B_1**	2012	3. 0	$P2_12_12_1$	leu NC	met	met					x	-			x		x	x
**4V8B_2**										?		-			x		x	x
**4V8C_1**	2012	3.3	$P2_12_12_1$	leu NC	met	met			-		x	-			x			x
**4V8C_2**									-	?		-			x			x
**4V8D_1**	2012	3.3	$P2_12_12_1$	tyr	met	tyr					x	-			x			x
**4V8D_2**										?		-			x			x
**4V8E_1**	2012	3.3	$P2_12_12_1$	tyr NC	met	tyr					x	-			x			x
**4V8E_2**										?	x	-			x			x
**4V8F_1**	2012	3.3	$P2_12_12_1$	tyr NC	met	tyr					x	-			x			x
**4V8F_2**												-			x			x
**4V9A_1**	2013	3.3	$P2_12_12_1$		met				-		x	-			x			x
**4V9A_2**									-	?		-			x			x
**4V9B_1**	2013	3.1	$P2_12_12_1$		met						x	-			x			x
**4V9B_2**										?		-			x			x
**4WQ1_1**	2015	3.1	$P2_12_12_1$	tyr NC	met	tyr					x	-			x			x
**4WQ1_2**										?	x	-			x		x	x
**4WQR_1**	2015	3.1	$P2_12_12_1$	phe NC	met	phe					x	-			x		x	x
**4WQR_2**										?	x	-			x		x	x
**4WR6_1**	2015	3.05	$P2_12_12_1$	tyr NC	met	tyr					x	-			x			x
**4WR6_2**										?	x	-			x		x	x
**4WRA_1**	2015	3.05	$P2_12_12_1$	tyr NC	met	tyr					x	-			x		x	x
**4WRA_2**										?		-			x		x	x
**4WRO**	2015	3.05	$P2_12_12_1$	phe NC	met	phe						-			x		x	x
**4WSD_1**	2015	2.95	$P2_12_12_1$	phe NC	met	phe					x	-			x		x	x
**4WSD_2**										?		-			x		x	x
**4WSM_1**	2015	3.3	$P2_12_12_1$	leu NC	leu	leu					x	-			x			x
**4WSM_2**									-	?		-			x			x
**4WT1_1**	2015	3.05	$P2_12_12_1$	phe NC	met	phe					x	-			x		l	x
**4WT1_2**										?	x	-			x		l	x
**4WU1_1**	2015	3.2	$P2_12_12_1$		tyr NC	tyr						-			x			x
**4WU1_2**									-	?		-			x			x
**4WZD_1**	2015	3.1	$P2_12_12_1$		tyr	tyr						-			x		S	x
**4WZD_2**									-	?		-			x			x
**4WZO_1**	2015	3.3	$P2_12_12_1$	met	met	phe						-			x		l	x
**4WZO_2**										?		-			x		l	x
**4V51**	2006	2.8	$P2_12_12_1$	asl phe	met	phe			x	-	x	-	x		x		-	-
**4V5A**	2007	3.5	$P2_12_12_1$		asl phe	met			x	-	x	-	x		x		-	-
**4V5E**	2008	3.45	$P2_12_12_1$		phe	phe					x	-	x		x			x
**4V5C**	2009	3. 3	$P2_12_12_1$	phe	met	phe			x		x	-	x		x		S	x
**4V5D**	2009	3.5	$P2_12_12_1$	phe	phe	phe			x		x	-	x		x		S	x
**4V7J**	2009	3.3	$P2_12_12_1$		met	met			x		x	-	x		x		S	
**4V5J**	2010	3.1	$P2_12_12_1$		phe	phe			x		x	-	x		x		S	x
**4V5L**	2010	3.1	$P12_11$	trp	phe	phe			x		x	-	x		x		S	x
**4V5P**	2011	3.1	$P12_11$	trp NC	phe	phe			x		x	-	x		x		S	x
**4V5Q**	2011	3.1	$P12_11$	trp NC	phe	phe			x		x	-	x		x		S	x
**4V5S**	2011	3.1	$P12_11$	Trp NC	phe	phe			x		x	-	x		x		S	x
**4V5R**	2011	3.1	$P12_11$	trp	phe	phe			x		x	-	x		x		S	x
**4V8Q**	2012	3.1	$P12_11$	tm	met	met			x		x	-	x		x		S	x
**4V8N**	2013	3.1	$P2_12_12_1$	ile	ile	ile			x		x	-	x		x		S	x
**4V9H**	2013	2.86	$P12_11$		P/E hyb				x		x	-	x		x		S	x
**4V9I**	2013	3.3	$P2_12_12_1$	ser	ser	ser			x		x	-	x		x		S	x
**4V63**	2008	3.2	$P2_12_12_1$		met	met					x		x		x		x	x
**4V67**	2008	3.	$P2_12_12_1$		met	met					x		x		x		x	x
**4V83**	2011	3.5	$P2_12_12_1$		IRES						x		x		x		x	x

**4V84**	2011	3.4	$P2_12_12_1$		IRES						x		x		x		x	x
**4V9N**	2013	3.4	$P2_12_12_1$		met						x		x		x		x	x
**4V9Q**	2013	3.4	$P2_12_12_1$		met	met					x		x		x		x	x
**4V90**	2013	2.95	$P12_11$		P/E hyb				x				x		x		S	
**4V8X**	2013	3.35	$P2_12_12_1$		met	met			x		x		x		x		S	x
**4V9K**	2013	3.5	C121		P/E hyb						x		x				S	x
**4V9L**	2013	3.5	$P2_12_12_1$		P/E hyb						x		x		x		S	x
**1VVJ**	2014	3.44	$P2_12_12_1$	ASL SufA6	met						x				x		S	x
**4L47**	2014	3.22	$P2_12_12_1$	ASL SufA6	met						x				x		x	x
**4LNT**	2014	2.94	$P2_12_12_1$	ASL SufA6	met										x		x	x
**4LSK**	2014	3.48	$P2_12_12_1$	ASL SufA6	met										x		x	x
**4TUE**	2014	3.5		ASL SufJ	met						x				x		x	x
**4ZER**	2015	3.1	$P2_12_12_1$		met													
**4V6A_1**	2009	3.1	$P2_12_12_1$		met								x		x			
**4V6A_2**									x		x		x		x			x
**4V7L**	2010	3.	$P2_12_12_1$	gln	met	gln			x				x		x		S	x
**4V7W**	2010	3.	$P2_12_12_1$						x	x	x	x	x		x		x	x
**4V7X**	2010	3.	$P2_12_12_1$						x	x	x	x	x		x		x	x
**4V7Y**	2010	3.	$P2_12_12_1$						x	x	x	x	x		x		x	x
**4V7Z**	2010	3.1	$P2_12_12_1$						x	x	x	x	x		x		x	x
**4V8A**	2012	3.2	$P2_12_12_1$															
**4V8G**	2012	3.	$P2_12_12_1$															
**4V8H**	2012	3.1	$P2_12_12_1$															
**4V8I**	2012	2.7	$P2_12_12_1$															
**4V95**	2012	3.2	$P2_12_12_1$		met													
**1VY4**	2014	2.6	$P2_12_12_1$	phe	met	phe											S	
**1VY5**	2014	2.55	$P2_12_12_1$	phe	met	phe											S	
**1VY6**	2014	2.9	$P2_12_12_1$	Mimic	met												S	
**1VY7**	2014	2.8	$P2_12_12_1$	Mimic	met												S	
**4V9R**	2014	3.	$P2_12_12_1$		met												S	
**4V9S**	2014	3.1	$P2_12_12_1$		met												S	
**4W2E**	2014	2.9	$P2_12_12_1$		phe	phe												
**4W2F**	2014	2.4	$P2_12_12_1$	phe	met	phe											S	
**4W2G**	2014	2.55	$P2_12_12_1$	phe	met	phe											S	
**4W2H**	2014	2.7	$P2_12_12_1$		met	Phe aS											S	
**4W2I**	2014	2.7	$P2_12_12_1$	phe	met	phe											S	
**4WPO**	2015	2.8	$P2_12_12_1$	phe	met	phe											S	
**4WQF**	2015	2.8	$P2_12_12_1$		met	phe											S	
**4WQU**	2015	2.8	$P2_12_12_1$		phe	phe												
**4Y4O**	2015	2.3	$P2_12_12_1$															
**4Y4P**	2015	2.5	$P2_12_12_1$	phe	met	phe											S	
**4Z3Q**	2015	2.6	$P2_12_12_1$	phe	met	phe											S	
**4Z3R**	2015	3.1	$P2_12_12_1$															
**4Z3S**	2015	2.6	$P2_12_12_1$	phe	met	phe											S	
**4Z8C**	2015	2.5	$P2_12_12_1$		met													
**D. Radiodurans**																		
**2ZJR**	2008	2.9	I222				x	x			-	-	x	x	x	x	x	
**2ZJQ**	2008	3.3	I222				x	x			-	-	x	x	x	x	x	
**3CF5**	2008	3.3	I222				x	x			-	-	x	x	x	x	x	
**3PIO**	2011	3.25	I222				x	x			-	-	x	x	x	x	x	
**4IO9**	2013	3.2	I222				x	x			-	-	x	x	x	x	x	
**4IOA**	2013	3.2	I222				x	x			-	-	x	x	x	x	x	
**5DM6**	2015	2.9	I222								-	-	x	x	x	x	S	
**E. Coli**																		
**4V52**	2007	3.2	$P2_12_12_1$					x						x				
**4V54**	2007	3.3	$P2_12_12_1$					x						x				
**4V57**	2007	3.5	$P2_12_12_1$					x						x				
**4V64**	2008	3.5	$P2_12_12_1$					x						x				
**4V6C_1**	2009	3.19	$P2_12_12_1$					x						x				
**4V6C_2**								x						x				x
**4V7U_1**	2010	3.1	$P2_12_12_1$					x						x				
**4V7U_2**								x						x				x
**4V7S_1**	2010	3.25	$P2_12_12_1$					x						x				
**4V7S_2**								x						x				x
**4V7V_1**	2010	3.29	$P2_12_12_1$					x						x				
**4V7V_2**								x						x				x
**4V7T_1**	2010	3.19	$P2_12_12_1$					x						x				
**4V7T_2**								x						x				x
**4WWW_1**	2010	3.1	$P2_12_12_1$					x						x				
**4WWW_2**								x						x				x
**4V9D**	2011	3.	$P2_12_12_1$		phe													
**4V85**	2012	3.2	$P2_12_12_1$					x						x				
**4V9C**	2012	3.3	$P2_12_12_1$		phe													
**4V9O_1,2**	2013	2.9	$P12_11$					x						x				
**4V9O_3,4**								x										
**4U1U**	2014	2.95	$P2_12_12_1$															
**4U1V**	2014	3.0	$P2_12_12_1$															
**4U20**	2014	2.9	$P2_12_12_1$															
**4U24**	2014	2.9	$P2_12_12_1$															
**4U25**	2014	2.9	$P2_12_12_1$															
**4U26**	2014	2.8	$P2_12_12_1$															
**4U27**	2014	2.8	$P2_12_12_1$															
**4WF1**	2014	3.1	$P2_12_12_1$															
**4WOI**	2015	3.0	$P2_12_12_1$		phe													
**4YBB**	2015	2.1	$P2_12_12_1$															

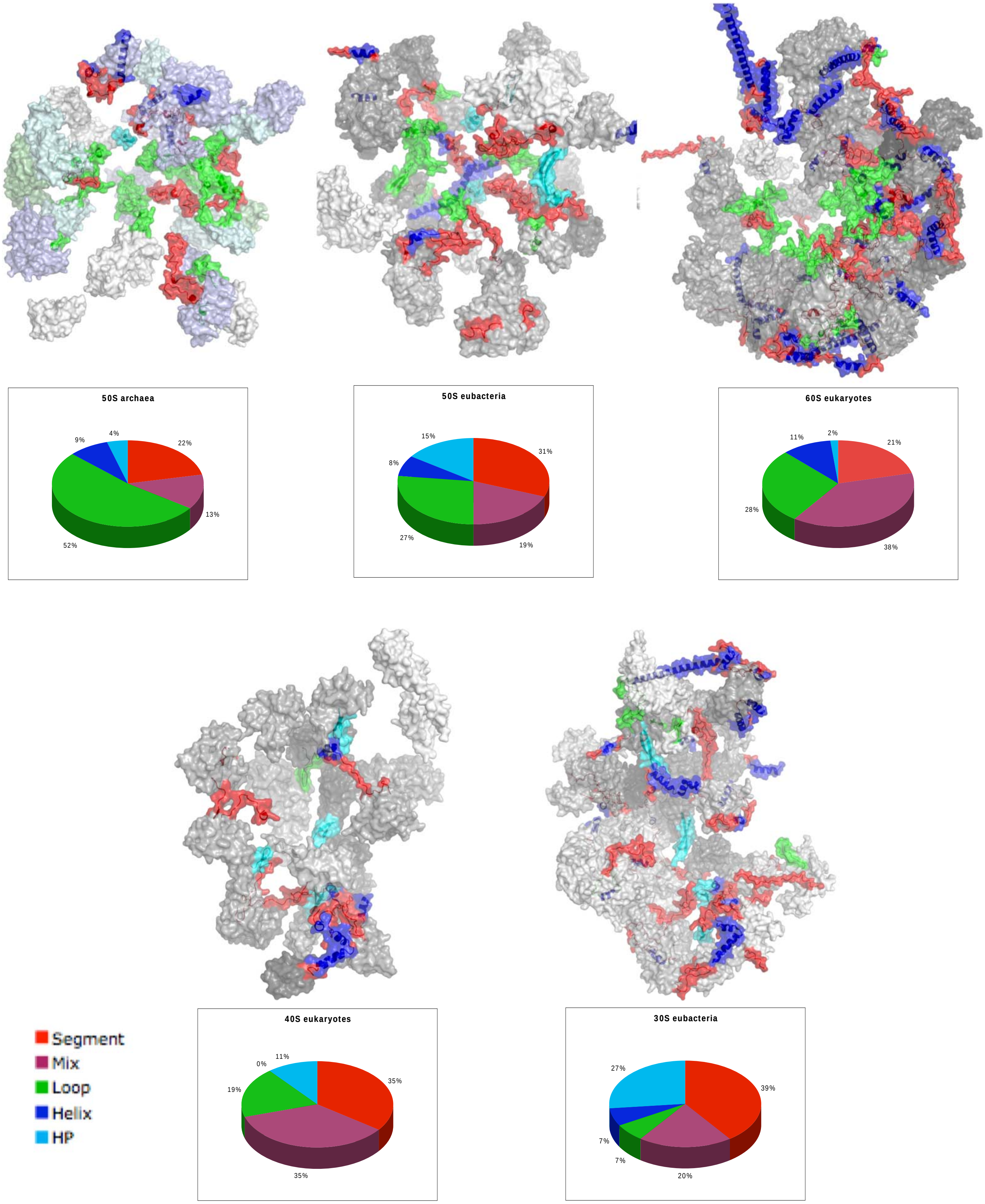

Figure S1
Statistic of the types of extensions of the archaeal (pdb_id: 1s72), eubacterial (pdb_id: 4v8i) and eukaryotic (pdb_id: 4v88) ribosomes

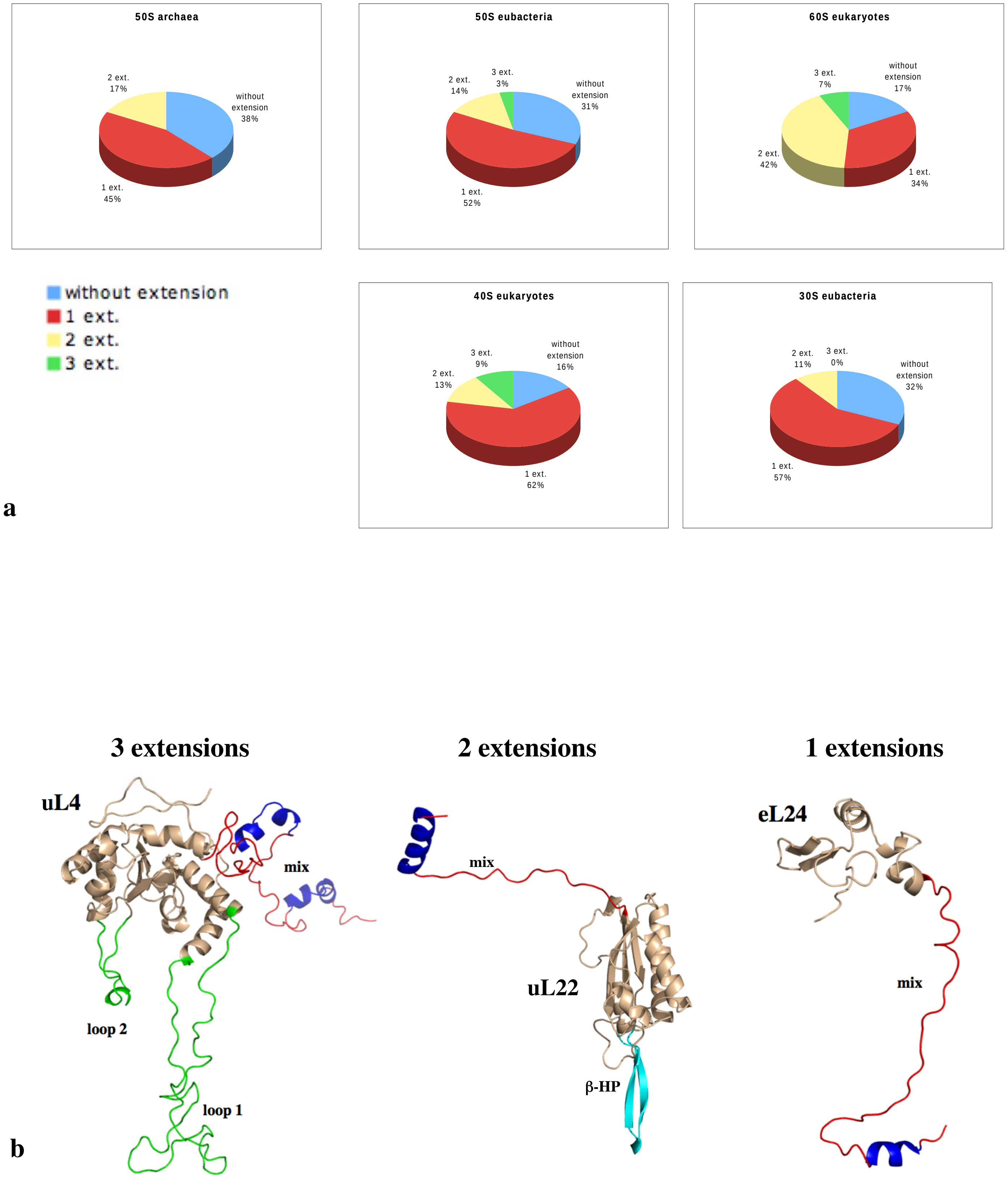

Figure S2

a. Statistic of number of extensions per protein within the archaeal (pdb_id: 1s72), eubacterial (pdb_id: 4v8i) and eukaryotic (pdb_id: 4v88) ribosomes

b. Examples of proteins with multiple extensions

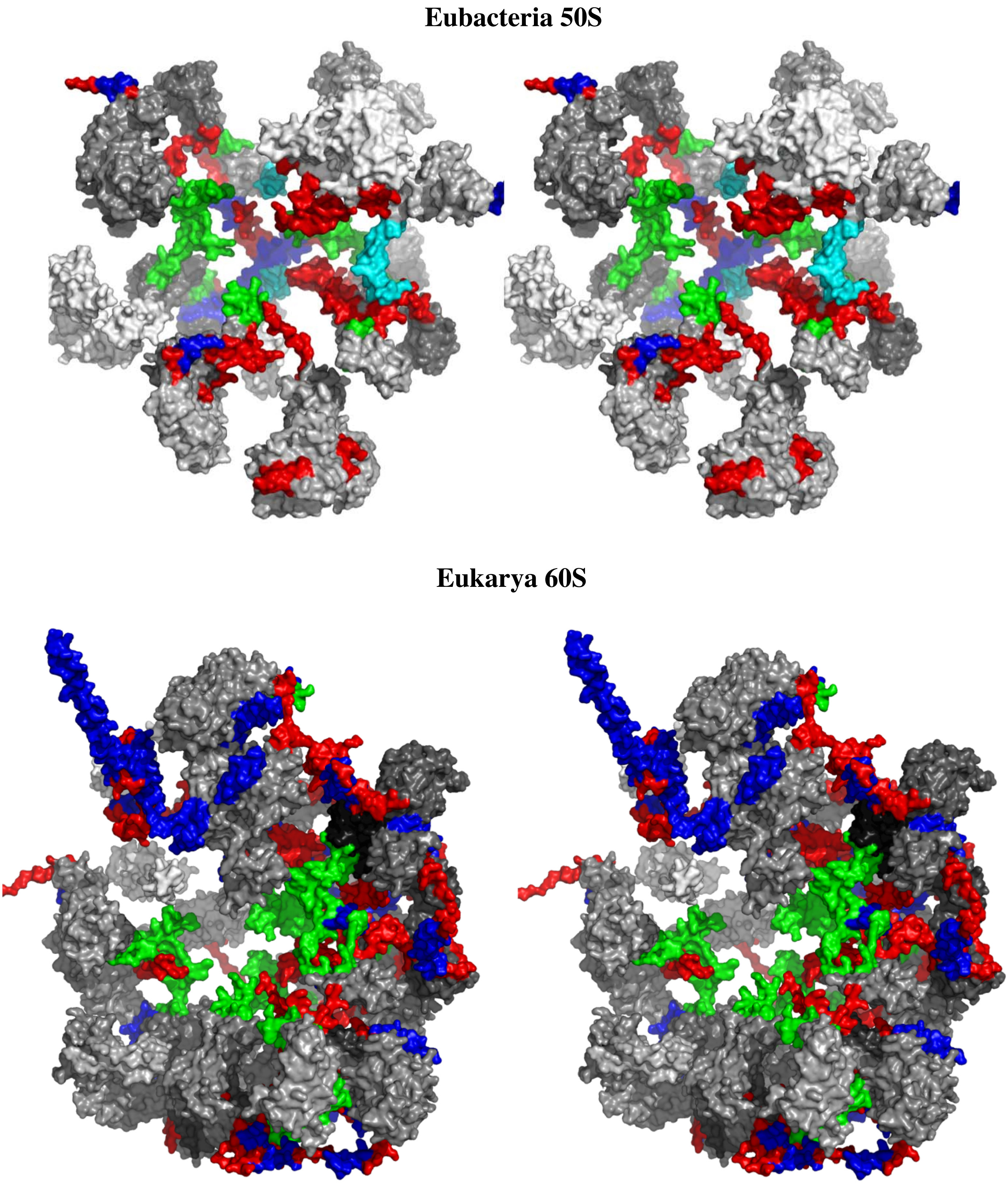

Figure S3 a: stereo views of the large subunits
(up) eubacteria (pdb_id: 4v8i)
(down) eukarya (pdb_id: 4v88)
Extensions are coloured according to their secondary structures
Green: loops, Red: segments, Blue: α-helices, Cyan: β-hairpins

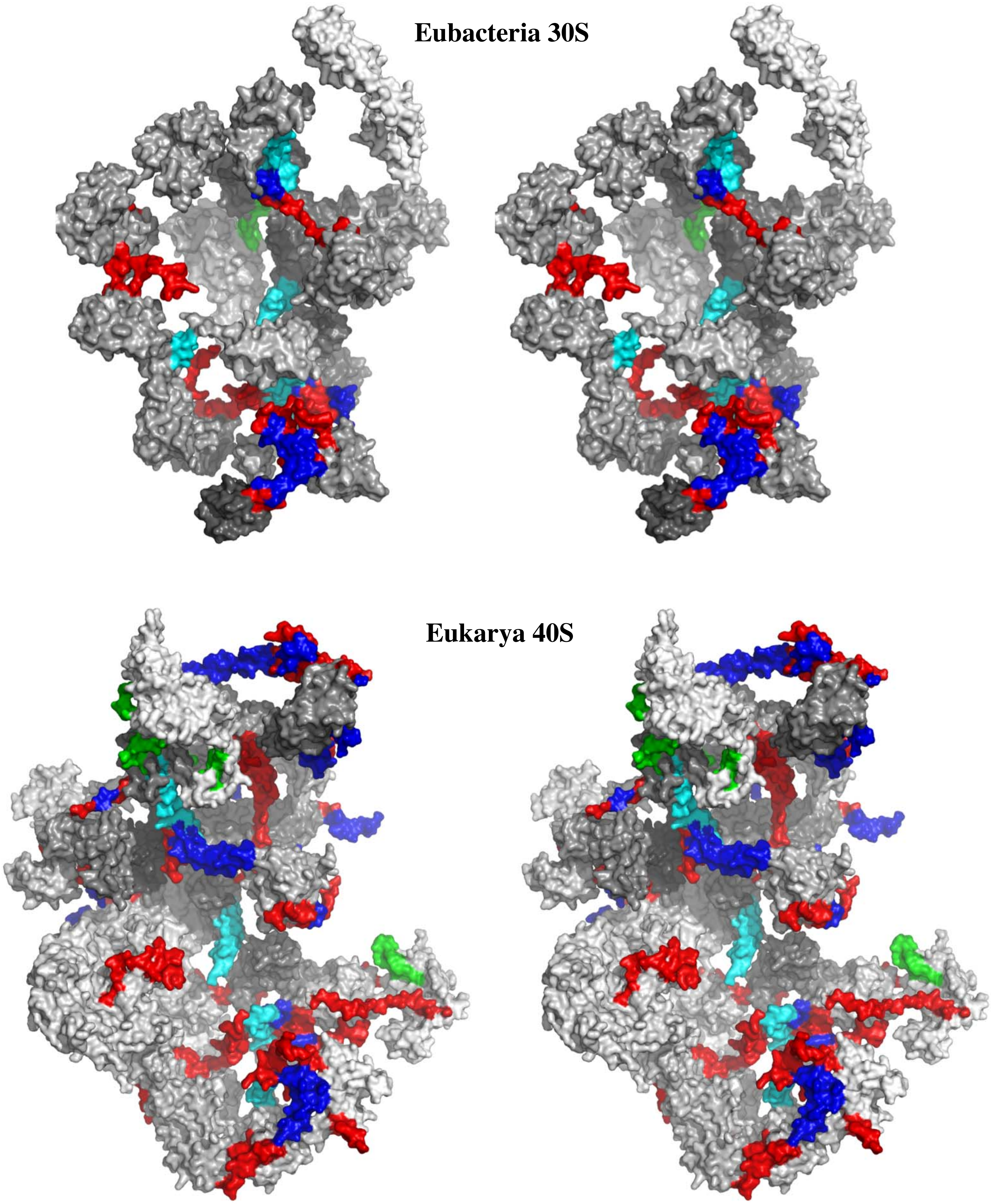

Figure S3 b: stereo views of the small subunits
(up) eubacteria (pdb_id: 4v8i); (down) eukaryotic ribosomes (pdb_id: 4v88)
Extensions are coloured according to their secondary structures
Green: loops, Red: segments, Blue: α-helices, Cyan: β-hairpins

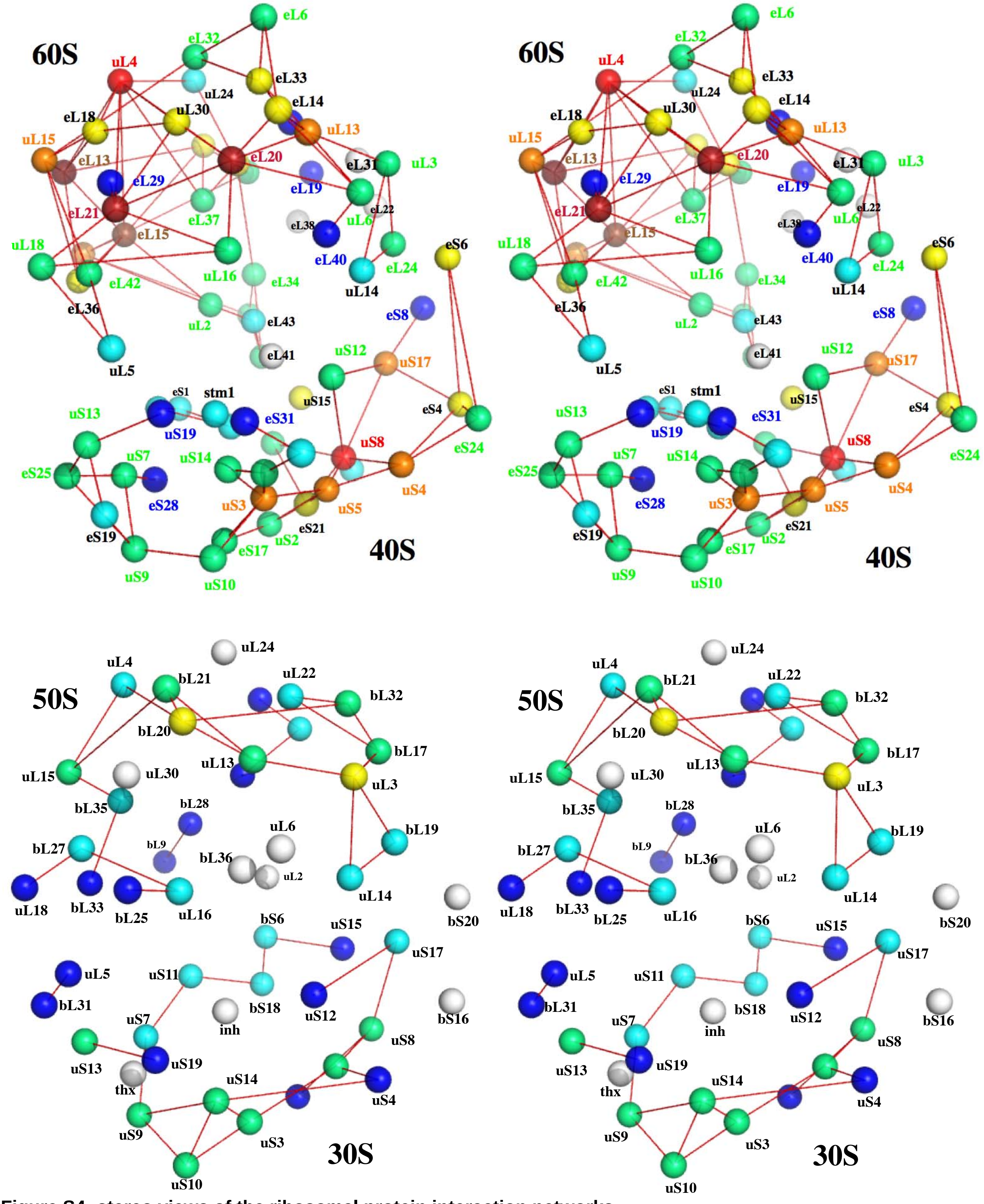

Figure S4: stereo views of the ribosomal protein interaction networks
(up) eukarya (pdb_id: 4v88) (down) eubacteria (pdb_id: 4v8i).The coloured spheres correspond to the centre of mass of the ribosomal proteins. Red lines indicate their connection within the ribosome structures. The proteins are coloured in function of their number of interacting partners following the same colour codes as in Figure 2 (main text).

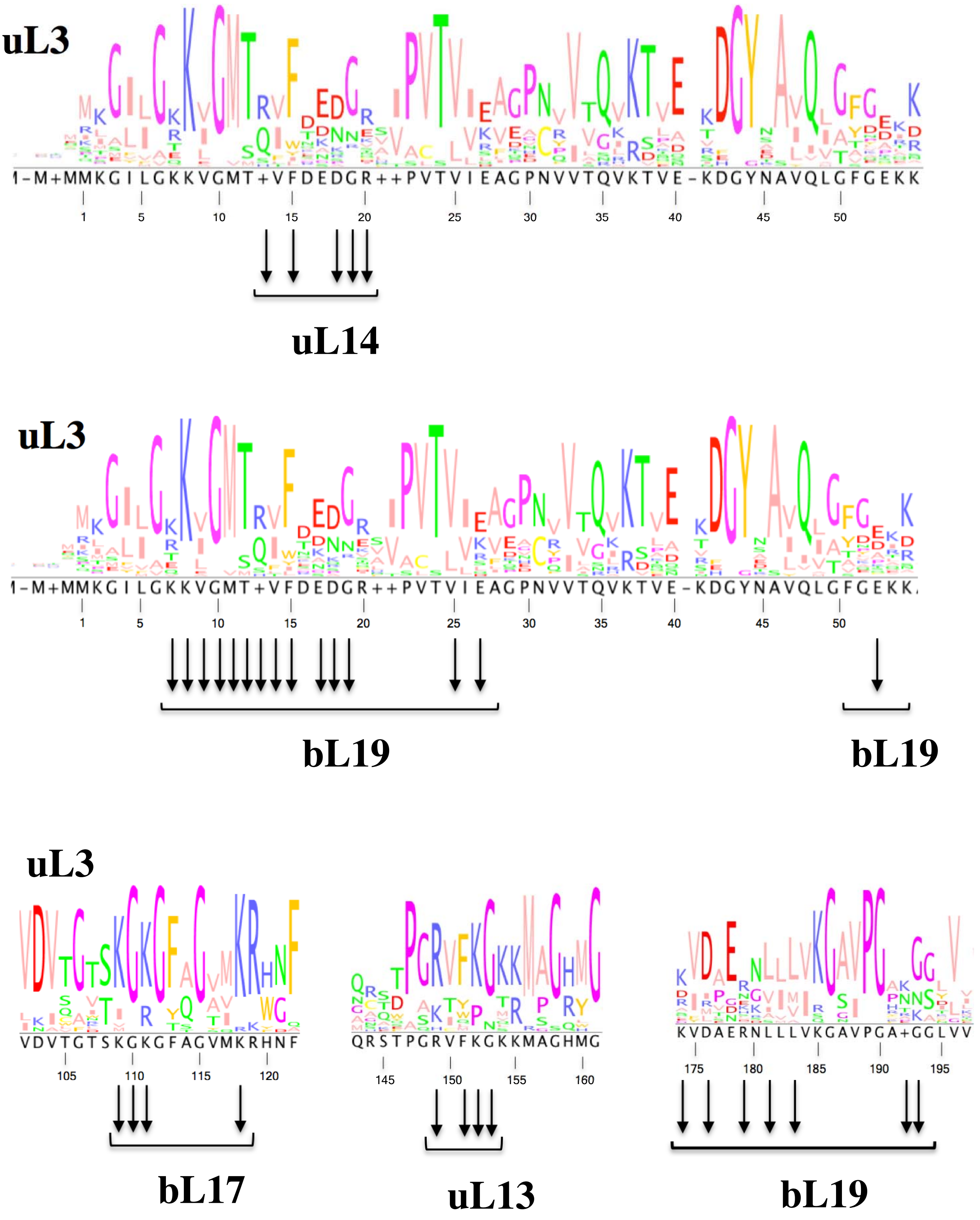

Figure S5: amino acids conservation at the protein interfaces

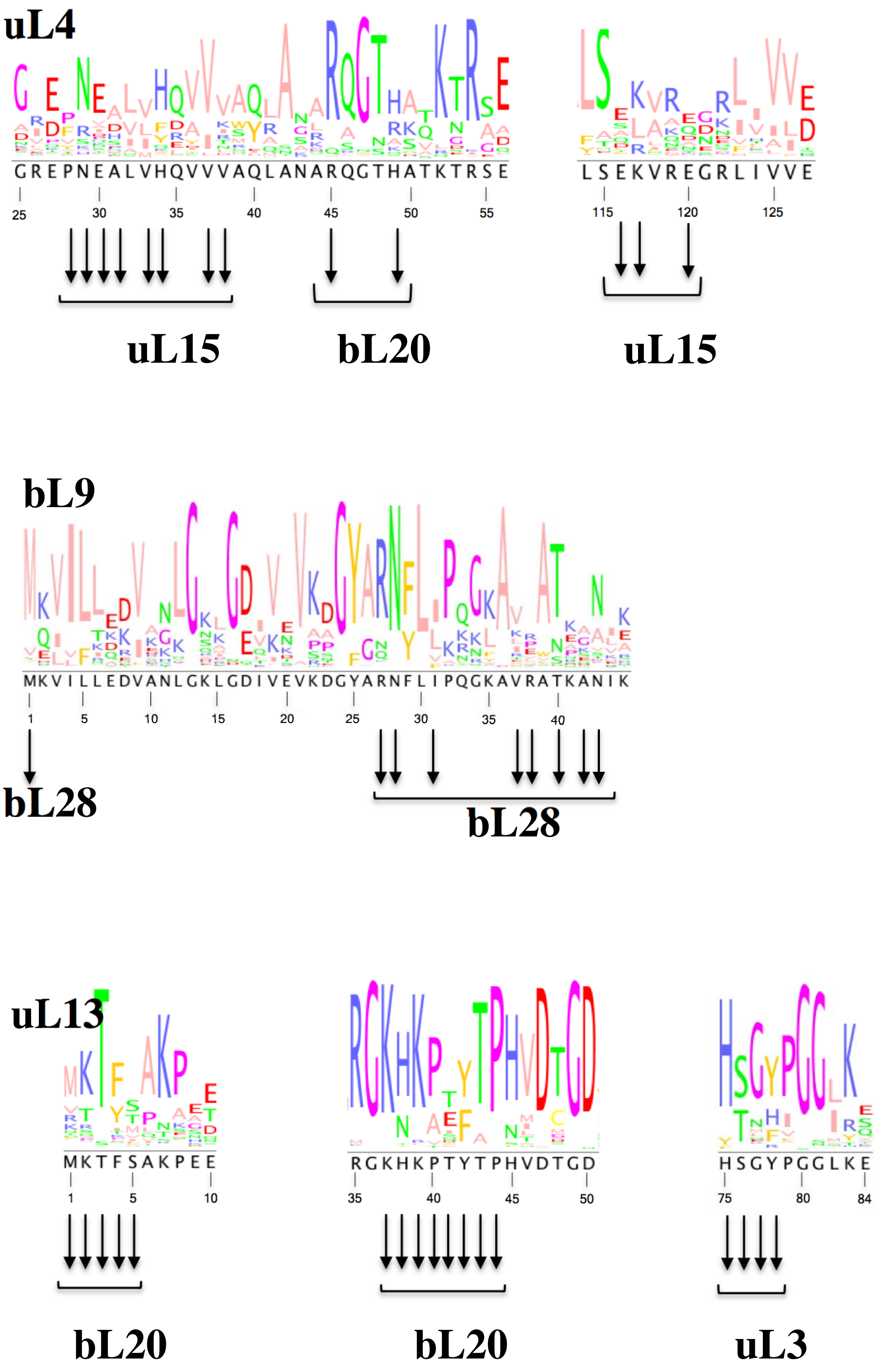

Figure S5 (continued)

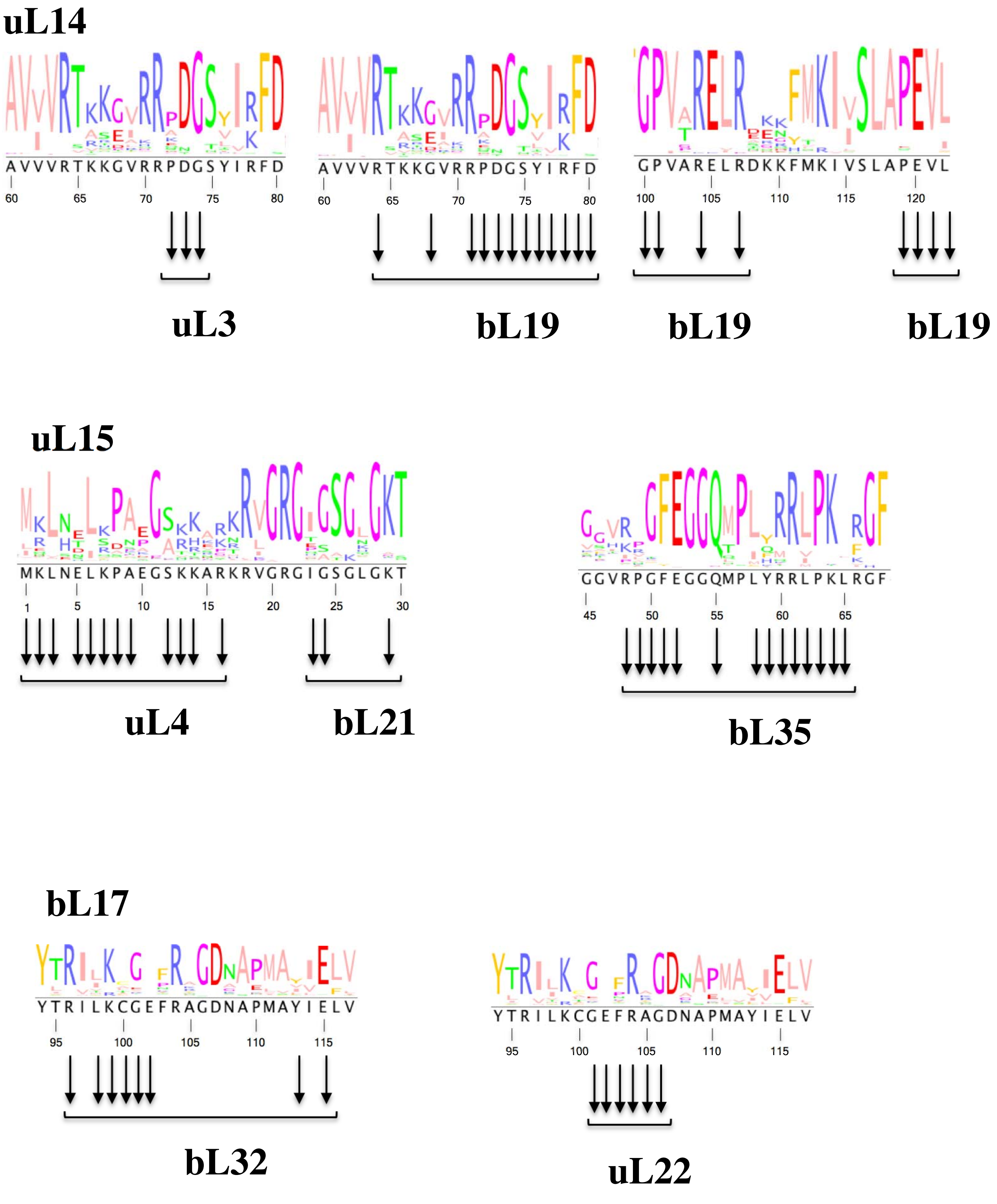

Figure S5 (continued)

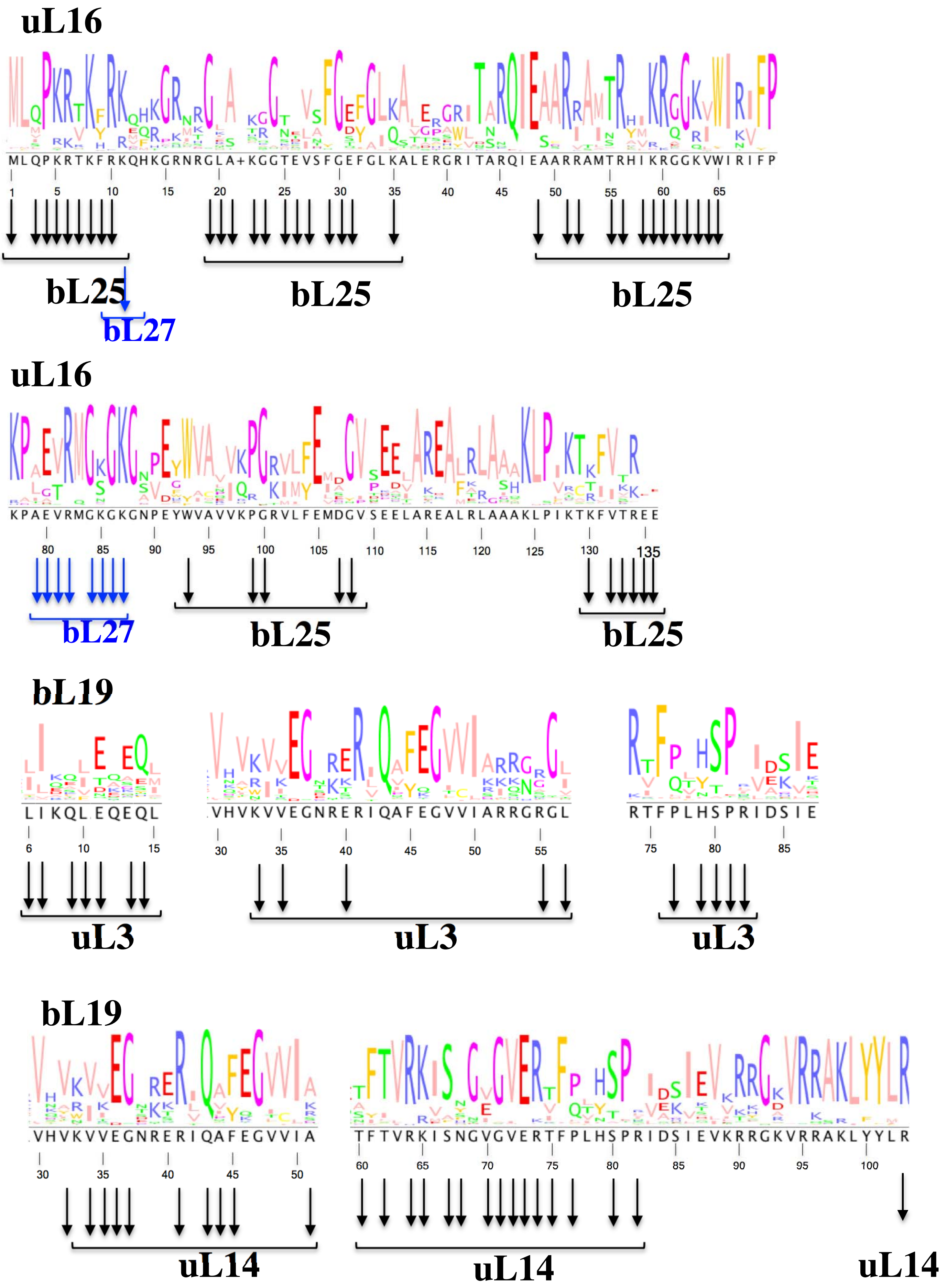

Figure S5 (continued)

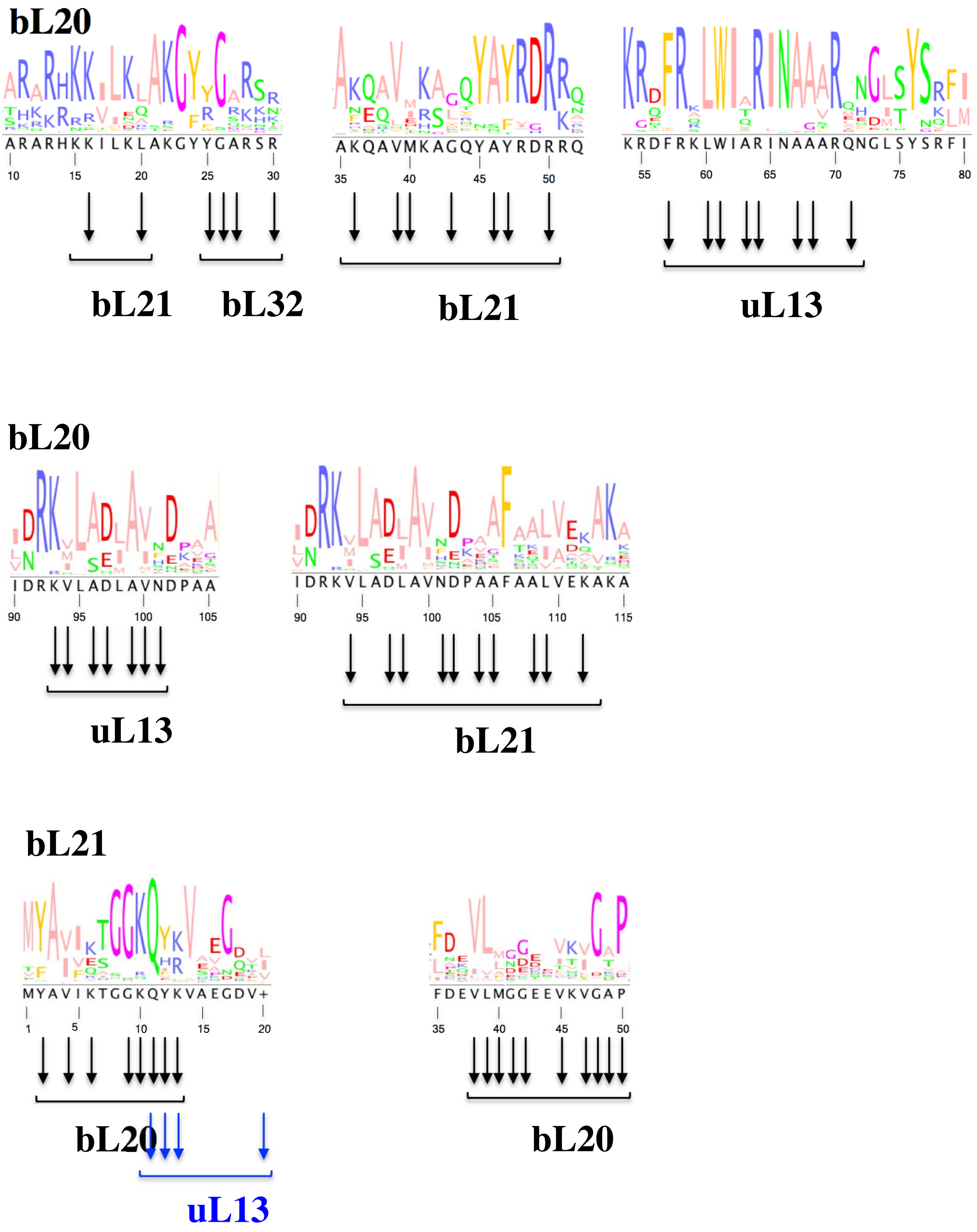

Figure S5 (continued)

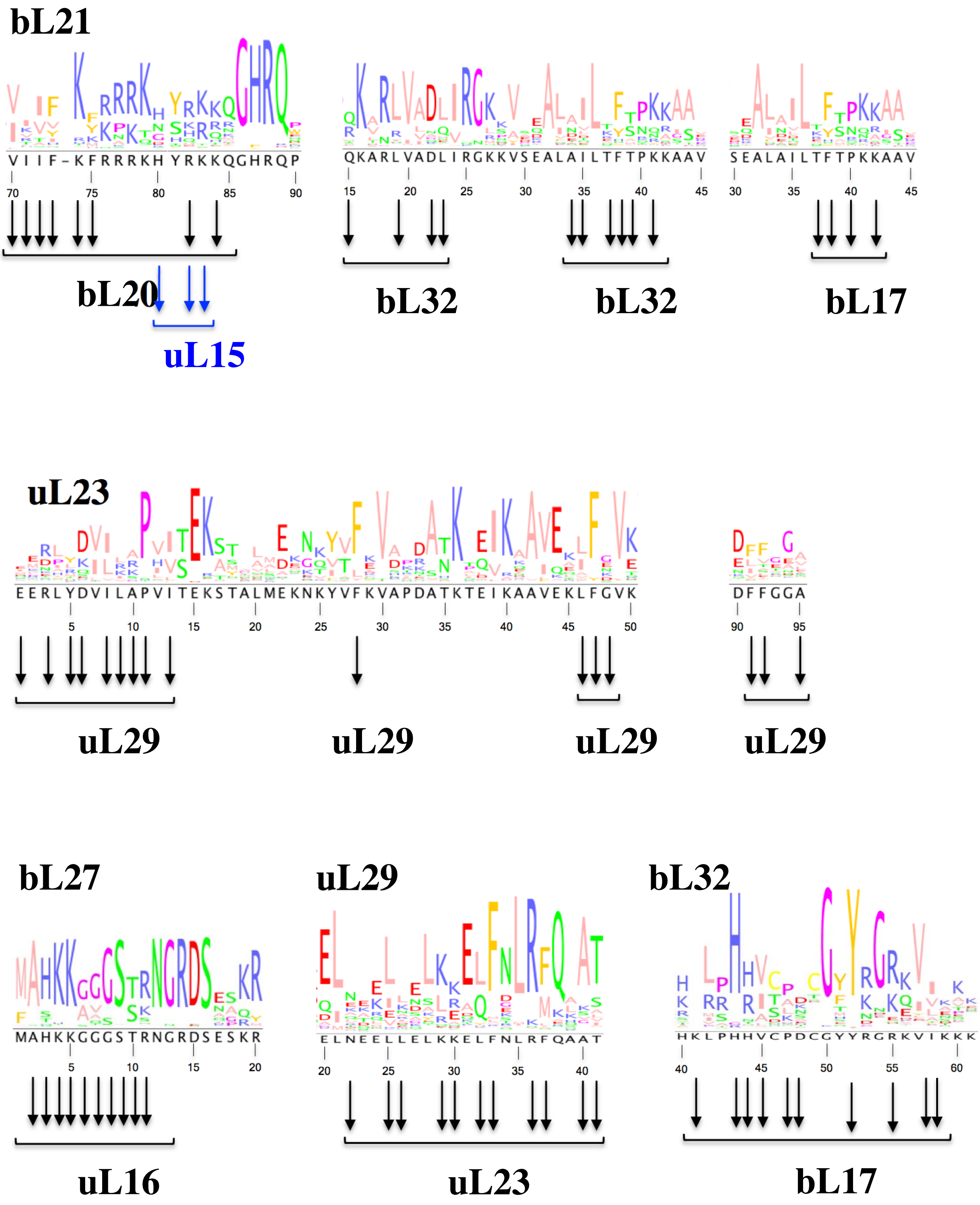

Figure S5 (continued)

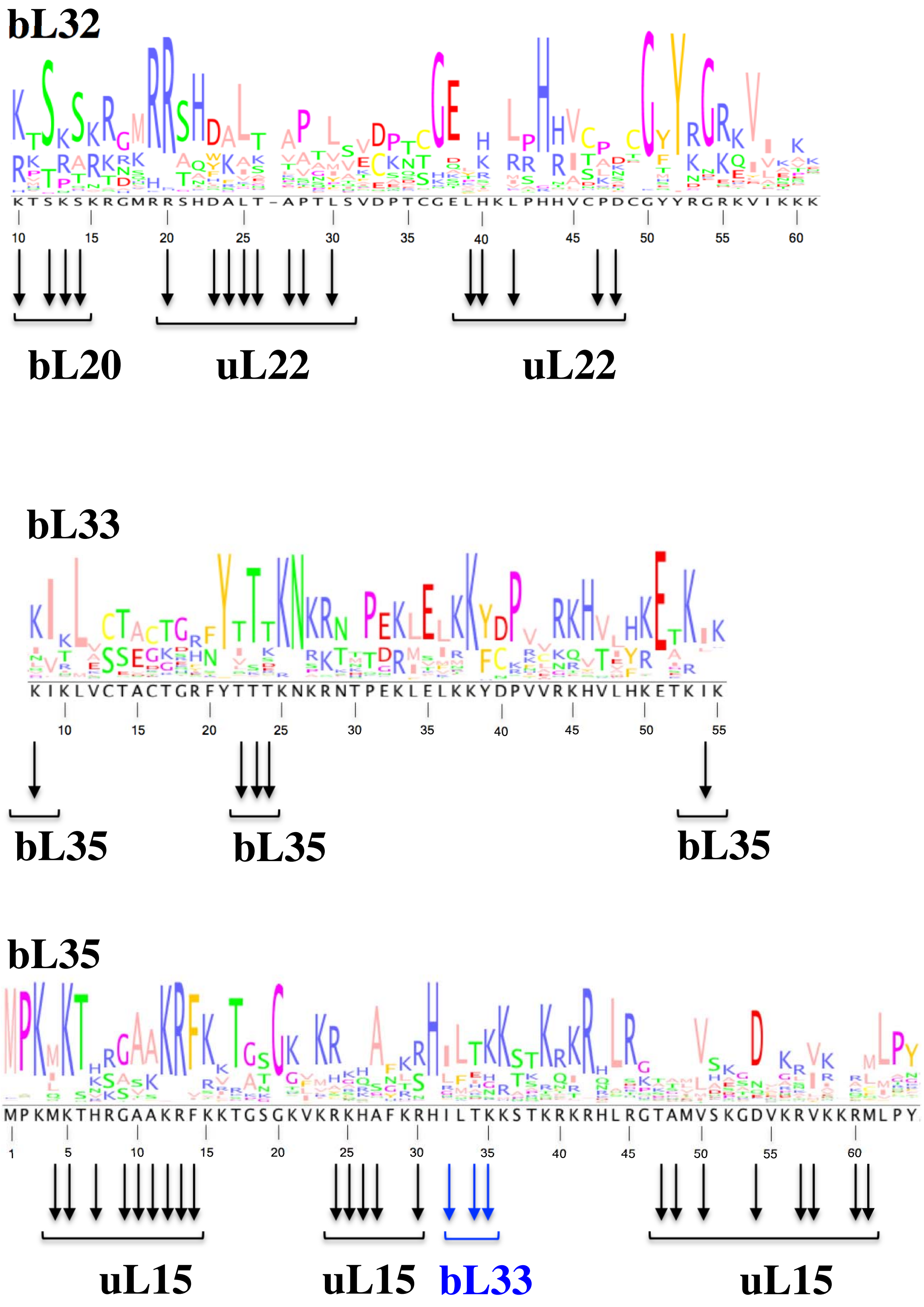

Figure S5: sequence conservation in the protein contact regions of ribosomal proteins of the 50S eubacterial subunit. The logo of the consensus sequences have been generated with *jalview* using the aligned eubacterial ribosomal protein sequences provided by U. Wolf and N. Yutin (see reference 15 and Supplementary Table S12). The arrows indicate the residues in contact with another protein.

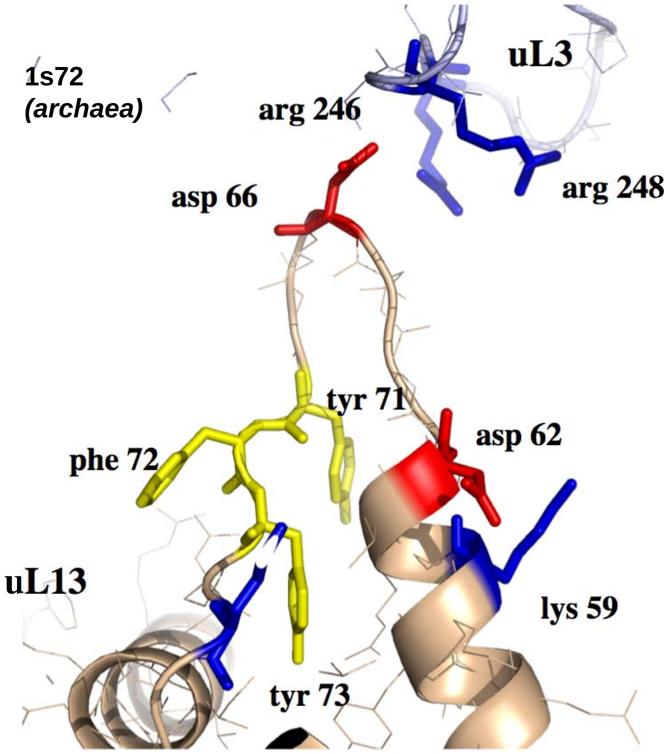

a

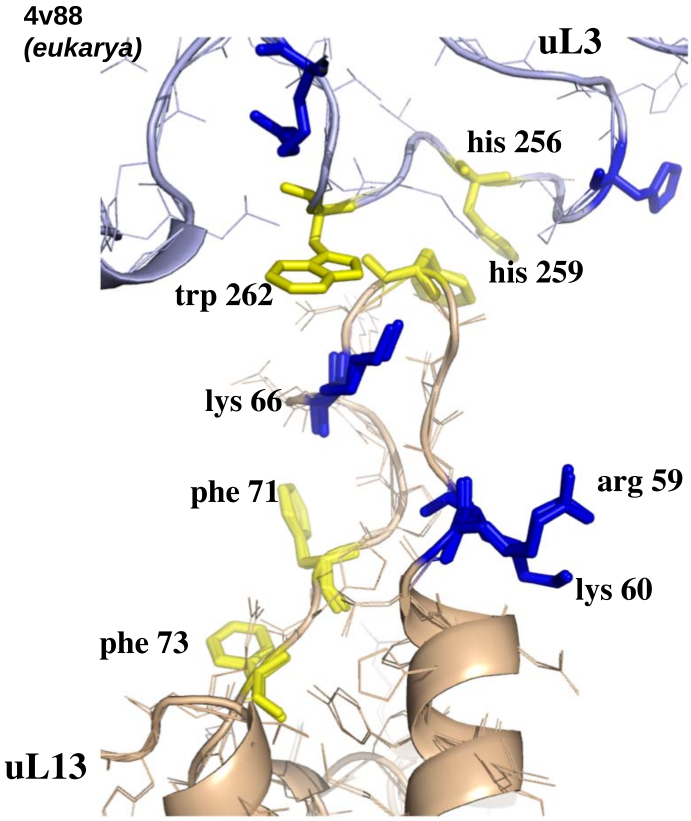

b

Figure S6: universal uL13-uL3 interaction
a: archaea (in which the cation-π interaction is replaced by a ionic one) and b: eukarya. Equivalent key residues are represented by coloured sticks.

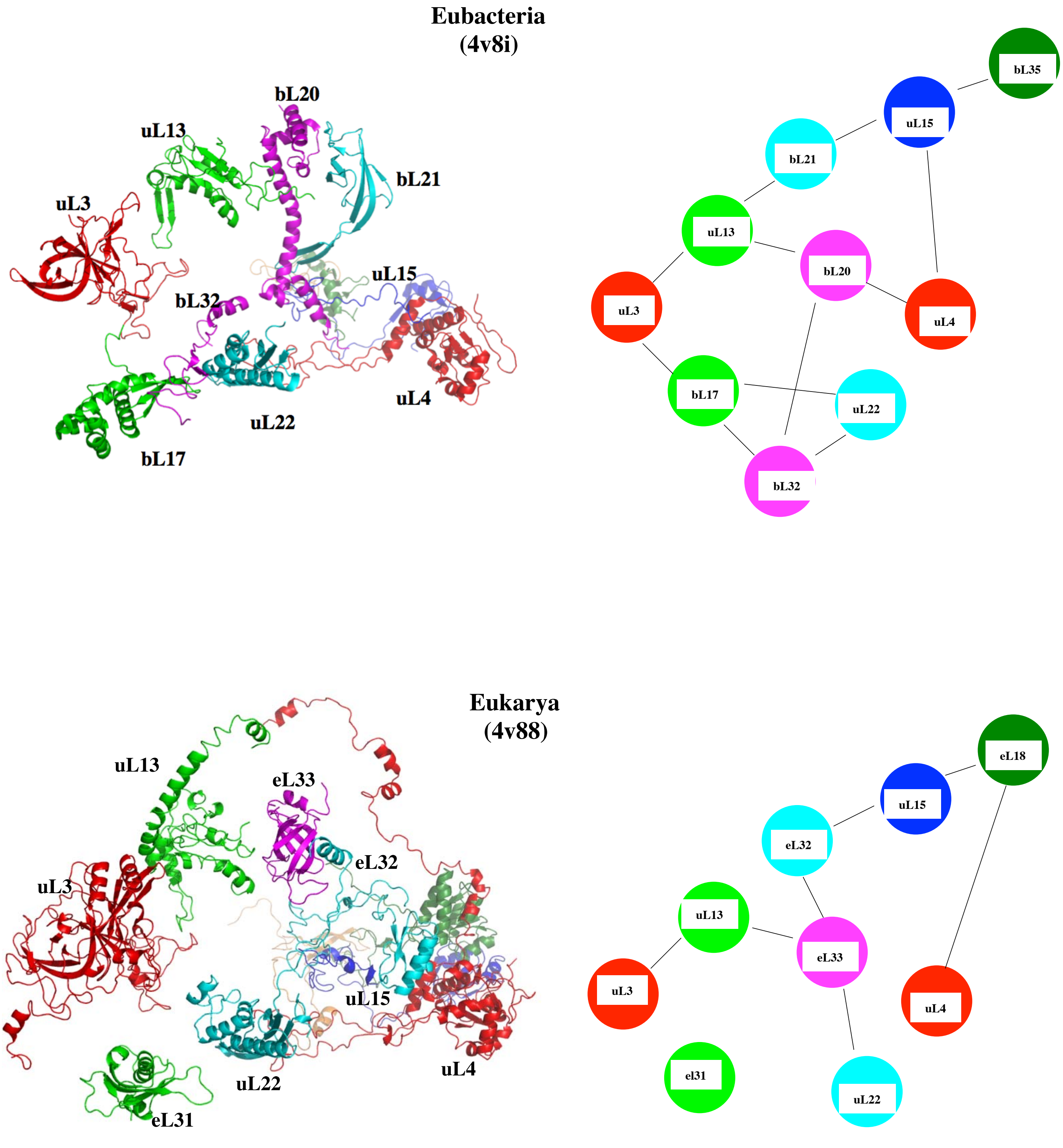

Figure S7: homologies and analogies in protein circuits
(up) 50S (pdb_id: 4v8i); (down) 60S (pdb_id: 4v88).
Equivalent protein are represented with the same colour.

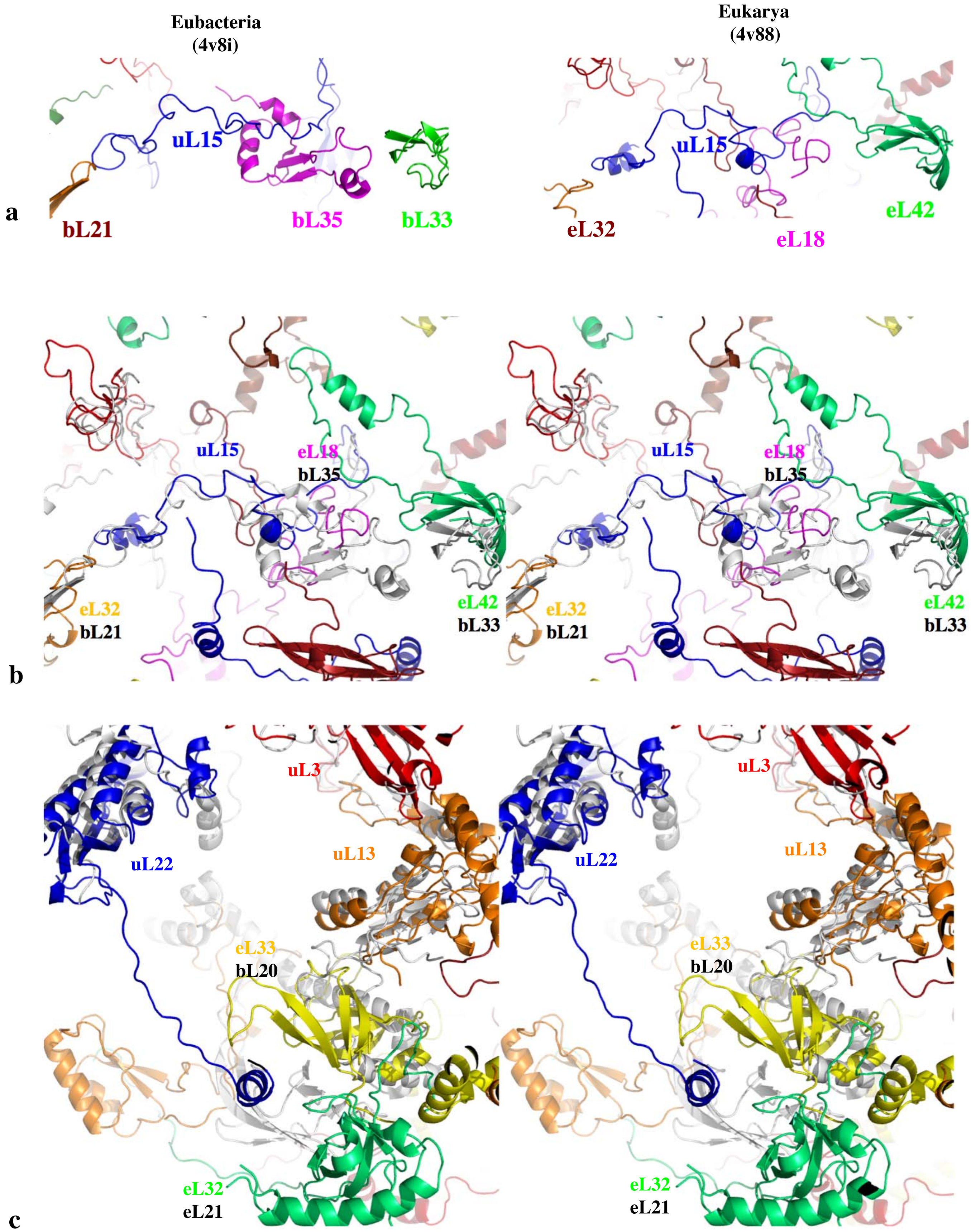

Figure S8: equivalent and alternative pathways in networks of the 50S and 60S subunits
a: Eubacterial (pdb_id: 4v8i) and eukaryotic (pdb_id: 4v88) circuits connecting the E-site
b: Stereo views of the superimposition of the equivalent proteins around the E-sites
c: Stereo views of the superimposition of the equivalent proteins around the exit tunnel
The bacterial proteins are represented in grey and the eukaryotic protein are coloured.

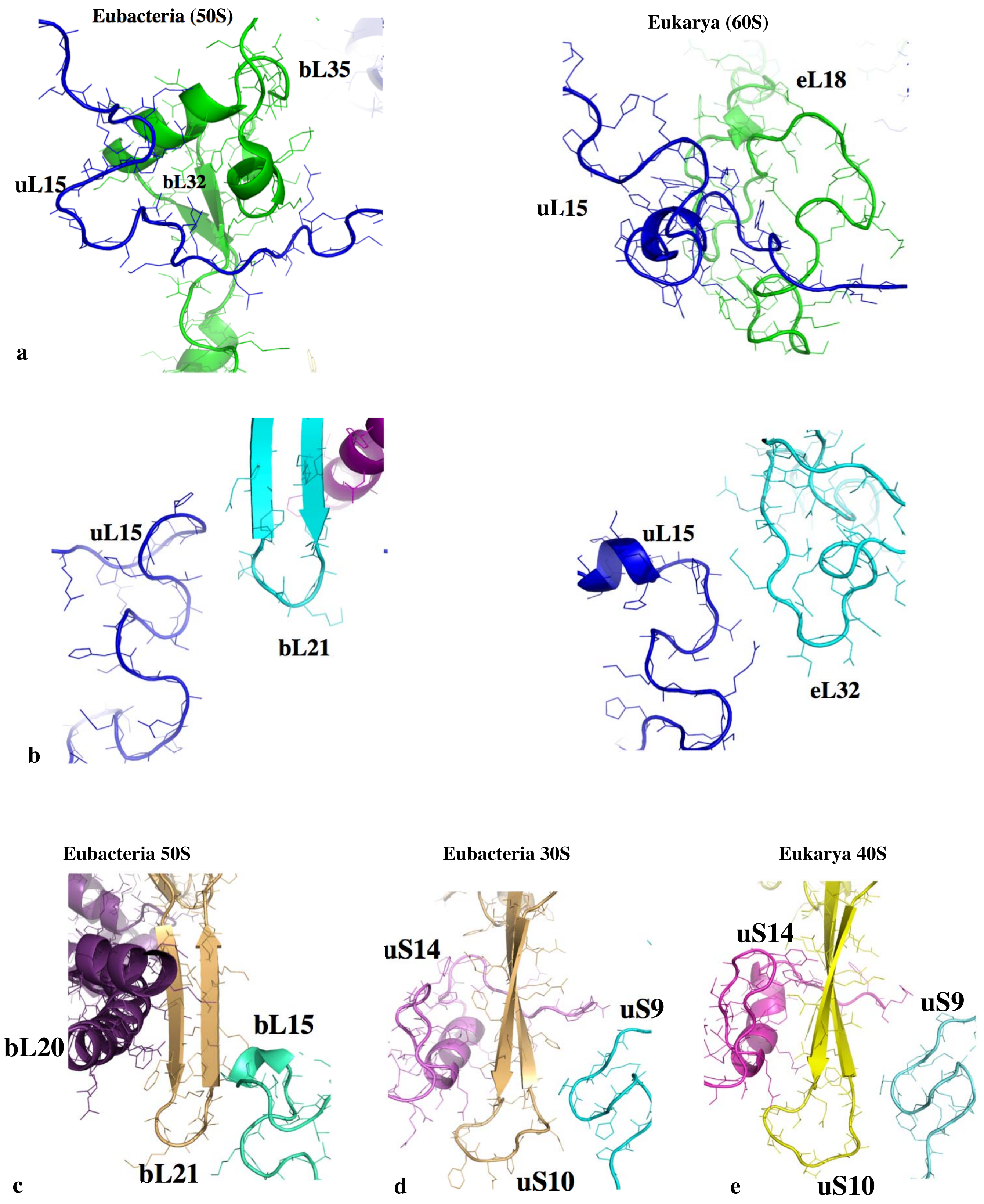

Figure S9 :recurrent interacting motifs in ribosomal subunits across the evolution

a: Comparison of uL15 - bL35 interaction in bacteria (pdb_id: 4v8i) and eukarya (pdb_id: 4v88).

b: Comparison of uL15 - bL21 interaction in bacteria (pdb_id: 4v8i) and eukarya (pdb_id: 4v88).

Recurrent triplet motif formed by a α-helix, a β-hairpin and a locally unstructured chain.

c: bL20-bL21-uL15 in eubacterial 50S subunit (pdb_id: 4v8i)

d: uS14-uS10-uS9 in eubacterial 30S subunit (pdb_id: 4v8i)

e: uS14-uS10-uS9 in eukaryotic 40S subunit (pdb_id: 4v88)

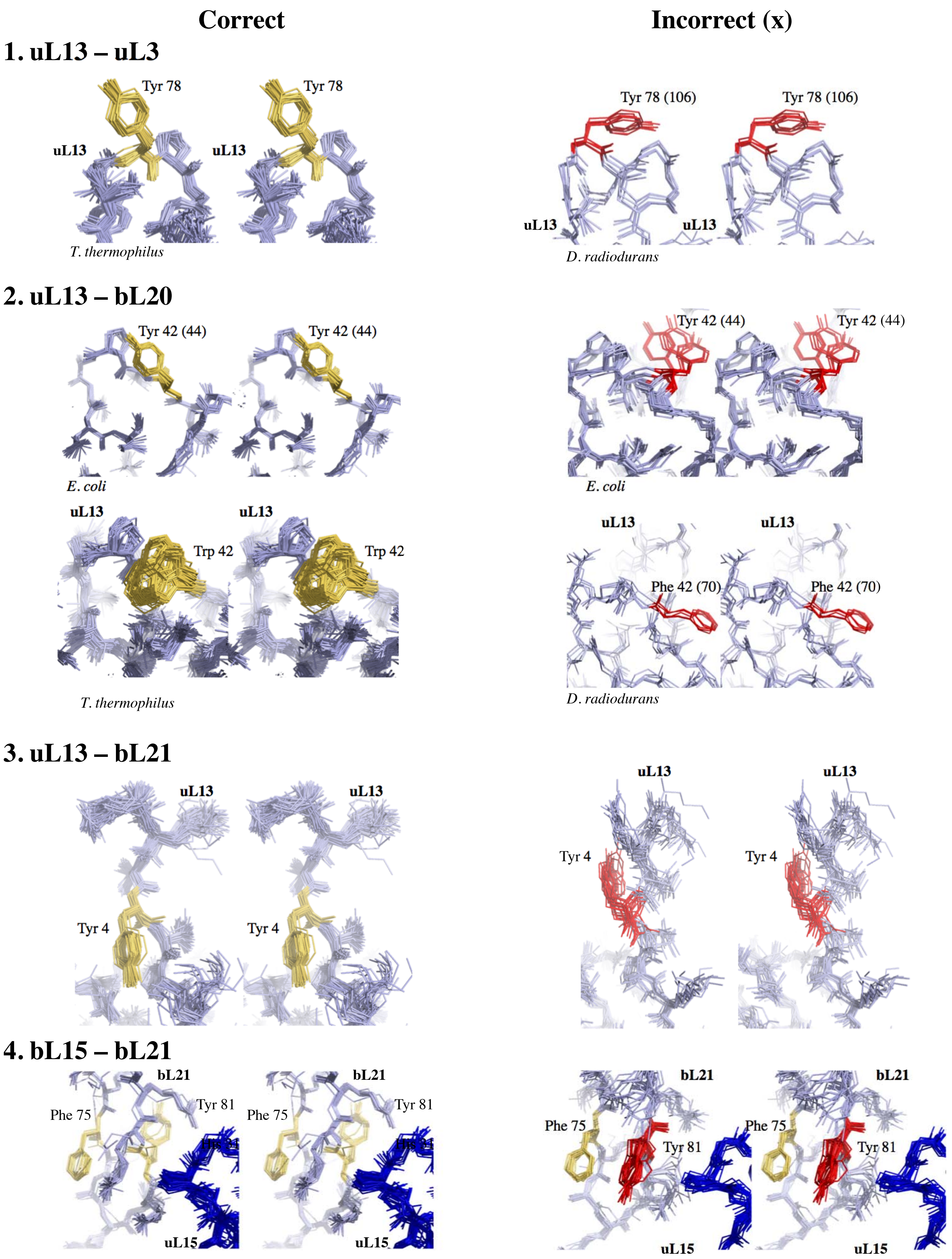

Figure S10: Correct and incorrect (x) models at the protein-protein interfaces of 50S and 70S pdb structures listed in table S15

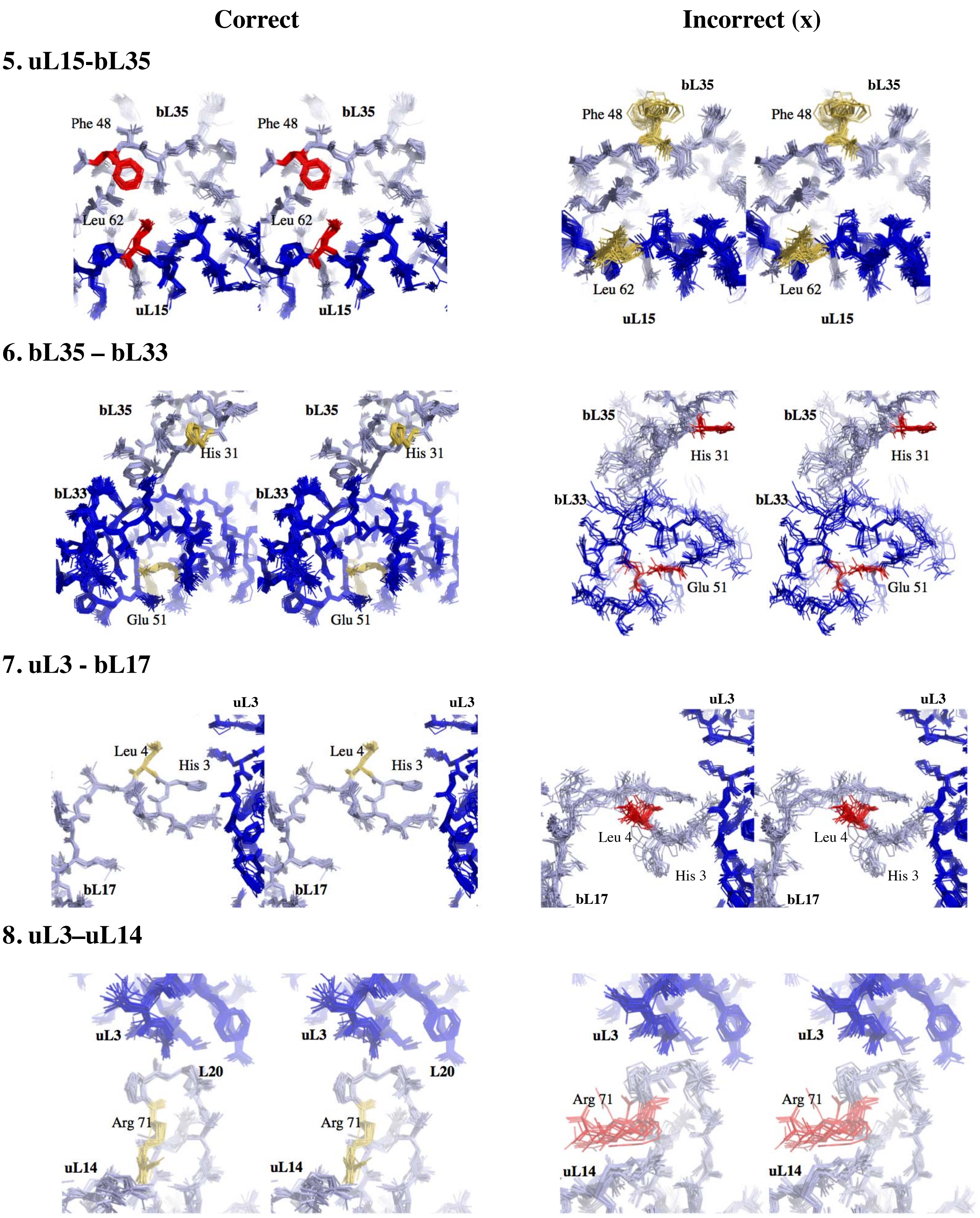

Figure S10: Correct and incorrect (x) models at the protein-protein interfaces of 50S and 70S pdb structures listed in table S15 (continued)

Correct | **Incorrect (x)**

9. bL17 - bL32

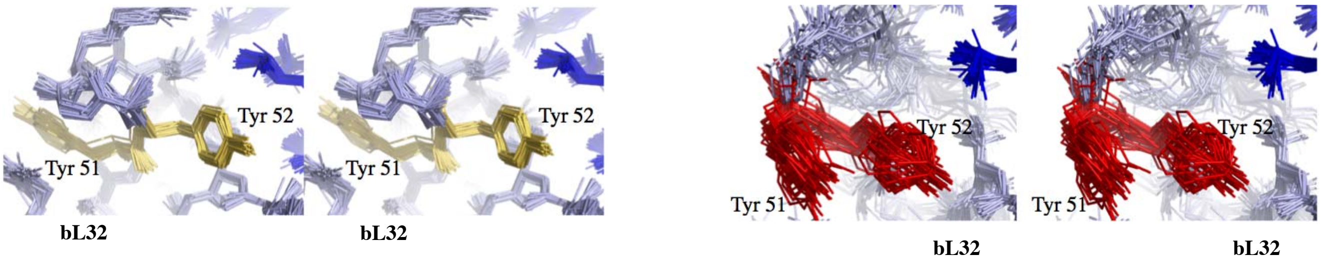

10. bL20 - uL4

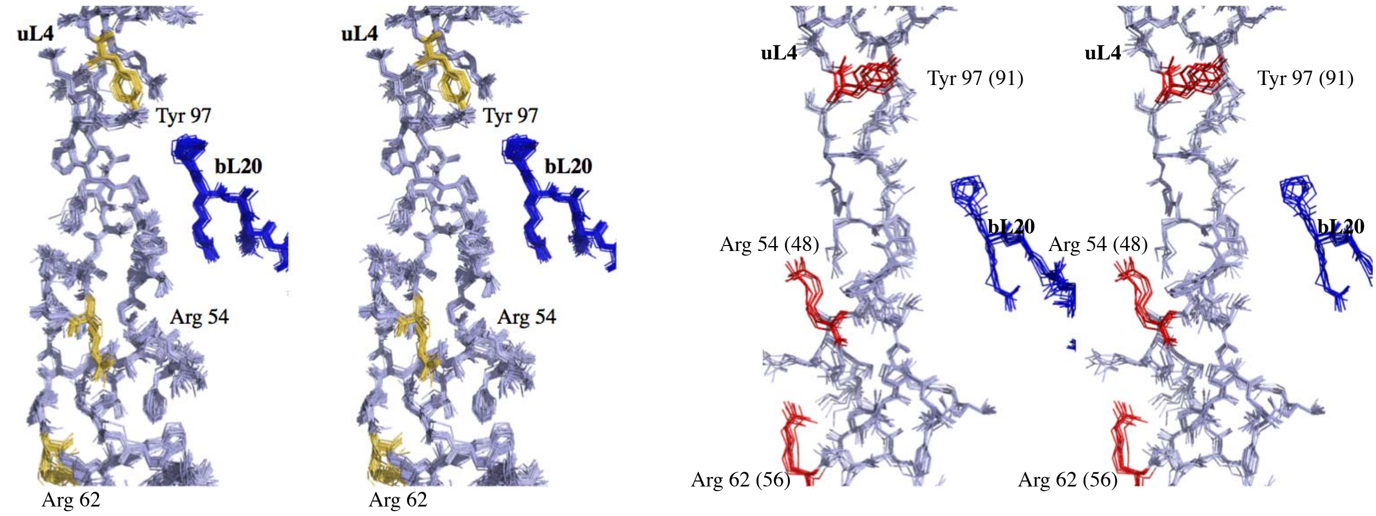

11. uL16 - bL27

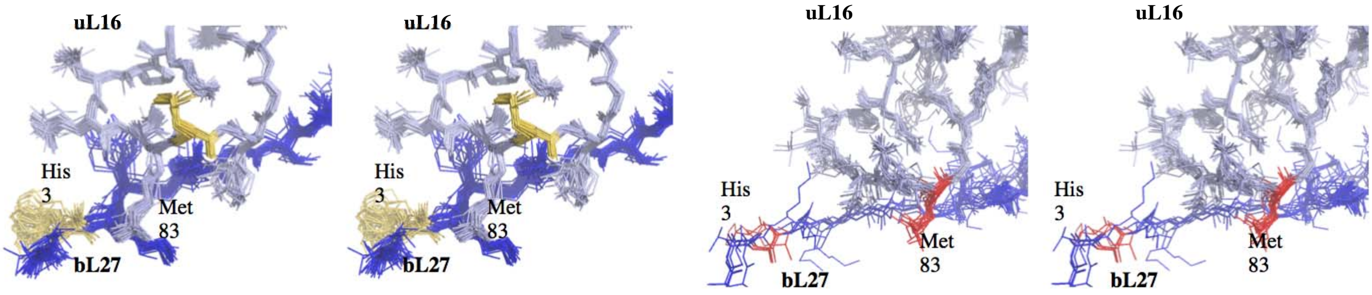

12. uL23 - uL29

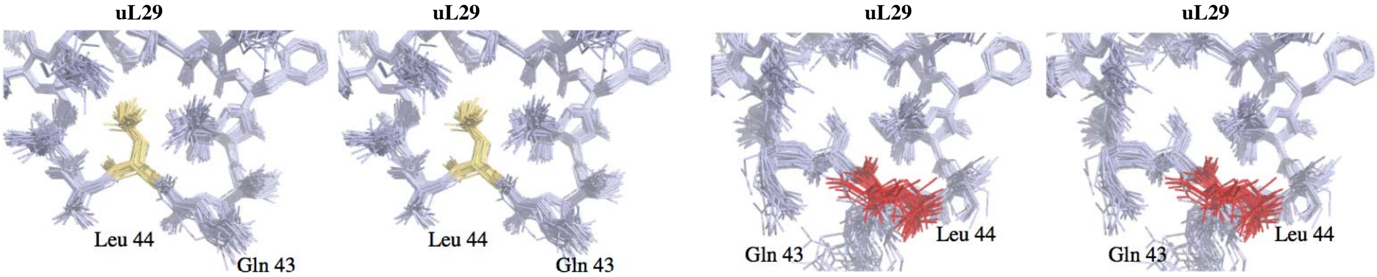

Figure S10: Correct and incorrect (x) models at the protein-protein interfaces of 50S and 70S pdb structures listed in table S15. Our careful inspection of electron density maps of each structure has revealed that except ul15-bL21 (that has been marked "?" in table S15), most of the models "x" are probably the results of a misinterpretation of a not well-resolved electron density map in the region.

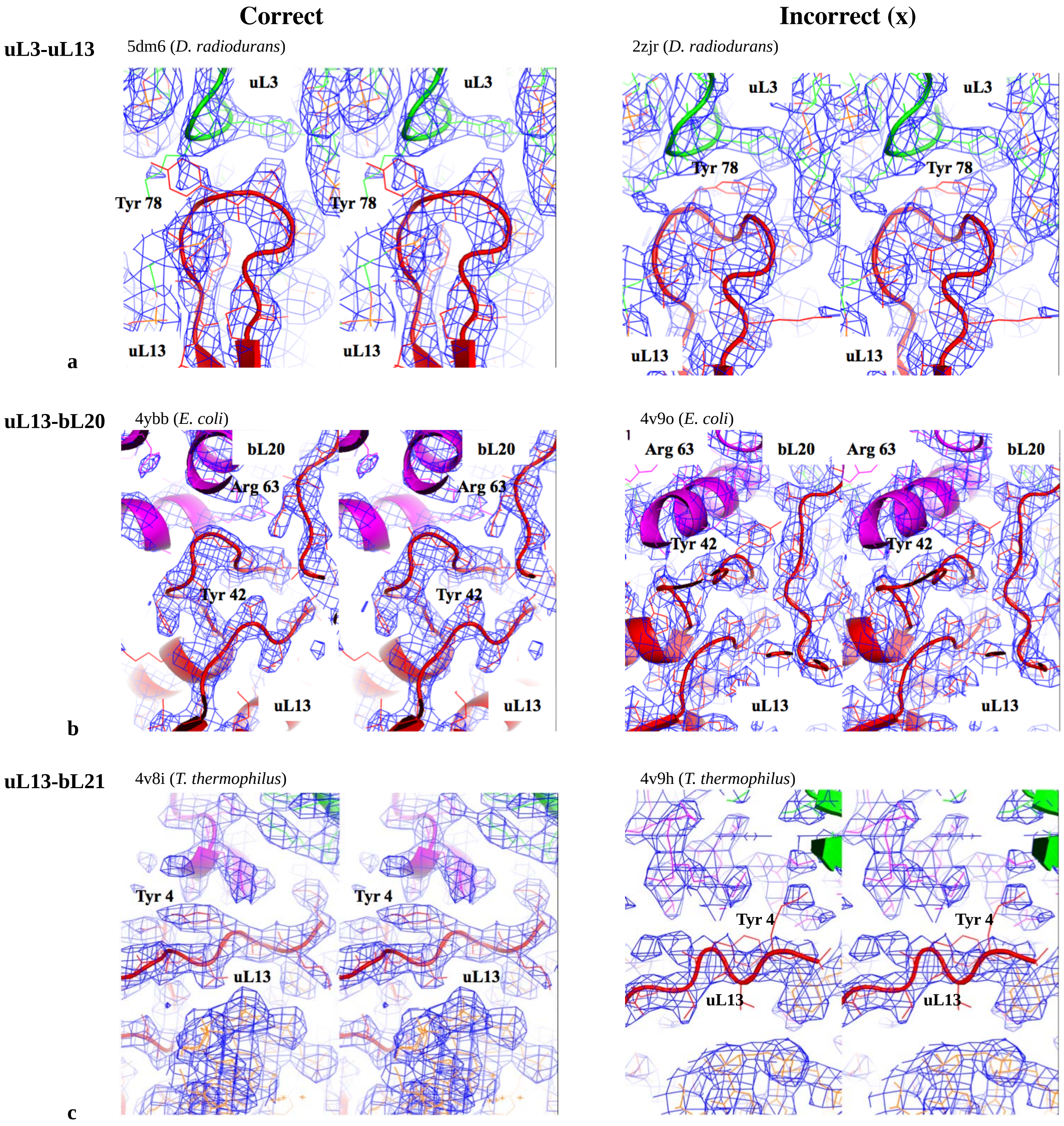

Figure S11: Comparison of the electron density maps of the alternative models at the protein-protein interfaces reported in table S15. Left: correct models; right: incorrect (x) models.

a: uL13-uL3 interface in the *D. radiodurans* 50S subunit
left: correct model (5dm6)
right: incorrect model (2zjr): tyr 106 (uL13) (78 in *T. thermophilus*) has been fitted into the density peak of gln 107 (uL13).
b: uL13-bL20 interface in the 70S *E. coli* 70S ribosome
left: correct model observed in the high resolution *E. coli* 70S ribosome 4ybb
right: incorrect model : in which tyr 42 (uL13) has been fitted into the density peak of arg 63 (bL20).
c: uL13-bL21 interface in the *T. thermophilus* 70S ribosome
left: correct model (4v8i)
right: incorrect model “1” in which tyr 4 (uL13) has been been erroneously fitted into the density of thr 3 (uL13).

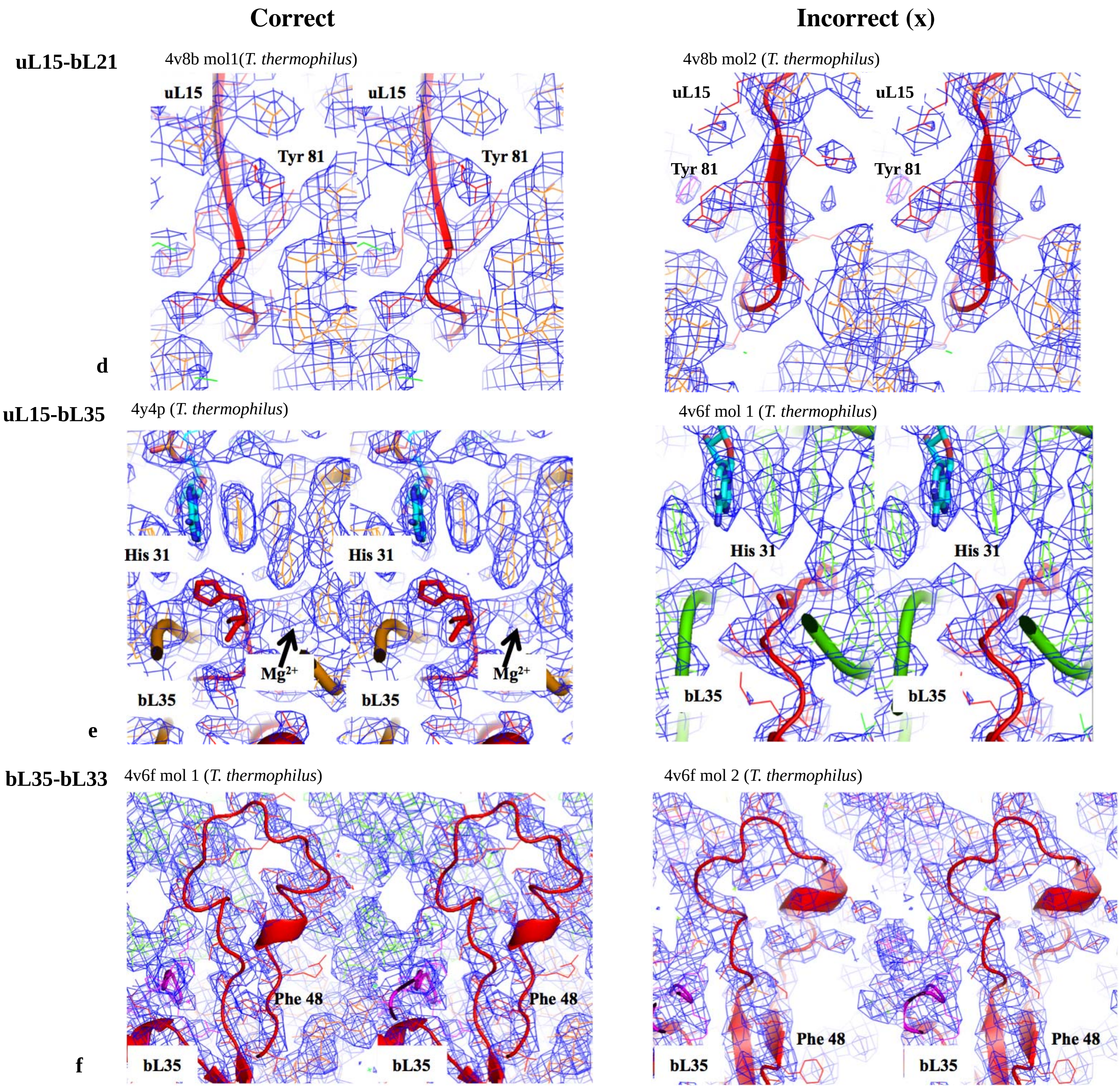

Figure S11 (continued)

d: uL15-bL21 interface in the *T. thermophilus* 70S ribosome

left: correct model observed in the first molecule of the asymmetric unit of 4v8b. The two aromatic residues of bL21 phe 75 and tyr 81 are on the opposite sides of the b-sheet.

right: alternative model “?” observed in the second molecule of the asymmetric unit of 4v8b. The two aromatic residues of bL21 phe 75 and tyr 81 are on the same side of the b-sheet. This alternative structure is compatible with the corresponding electron density map. However this structure only observed in a set of pdb entry marked “?” in table S15.

e: uL33-bL35 interface in the *T. thermophilus* 70S ribosome

left: correct model found in the 2.5 Å resolution 4y4p structure

right: incorrect model found in the molecule 2 of 4v6f structure. His 31 has been fitted into a peak that correspond to a Mg^{2+} ion in the high resolution structure.

f: uL15-bL35 interface in the *T. thermophilus* 70S ribosome (continued)

left: correct model found in the molecule 1 of 4v6f and other high-resolution structures of the *T. thermophilus* 70S ribosome.

right: incorrect model found in the molecule 2 of the 4v6f structure. Lys 47 has been fitted into the density peak of phe 48 that becomes extruded out of density.

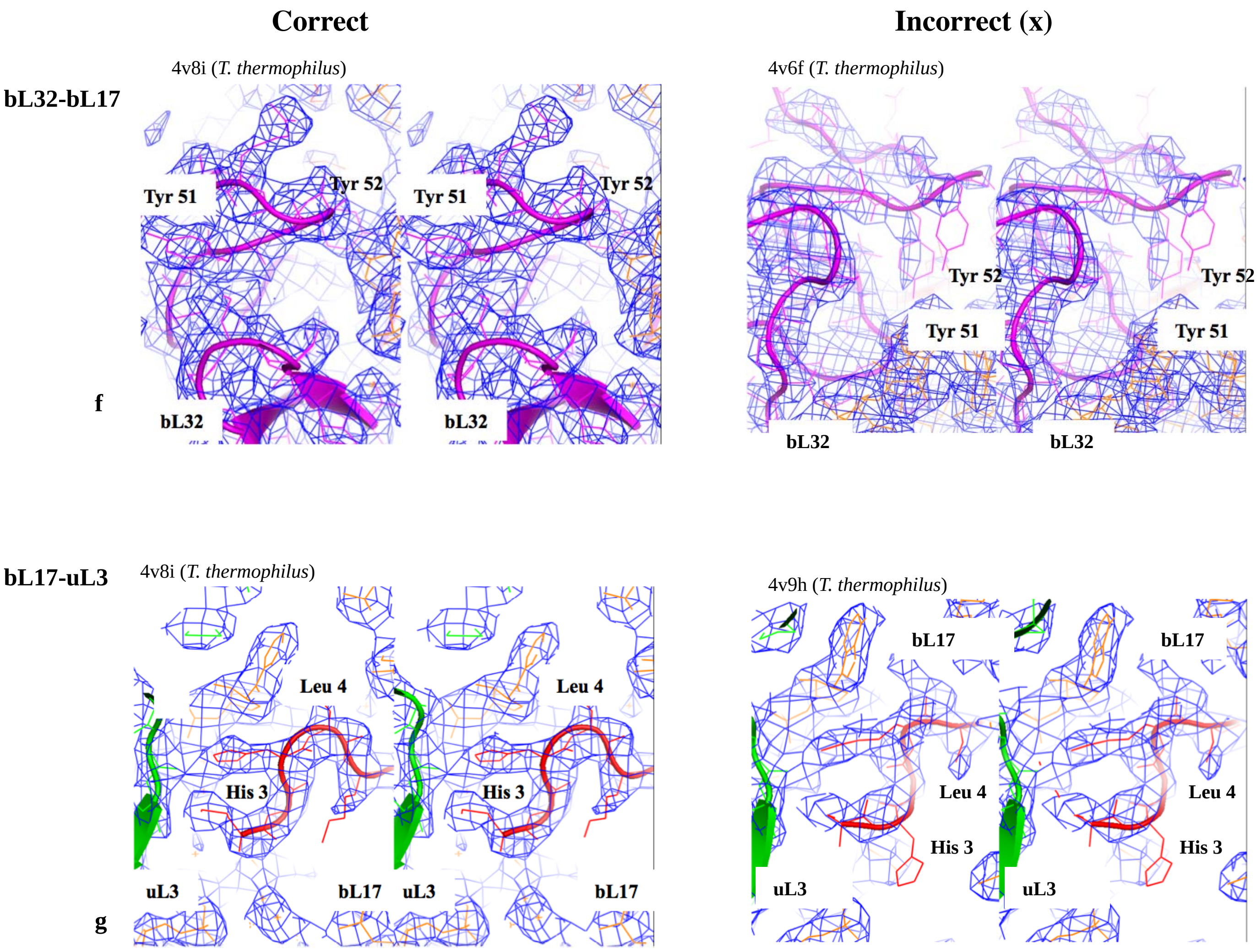

Figure S11 (continued)
g: bL17-bL32 interface in the *T. thermophilus* 70S ribosome
left: correct model found in 4v8i
right: incorrect model found in the structure of 4v6f. The tyrosines 51 and 52 of bL32 have been incorrectly fitted due to an interpretable map in this region.

h: bL17-uL3 interface in the *T. thermophilus* 70S ribosome
left: correct model found in 4v8i and other high-resolution structures of the *T. thermophilus* 70S ribosome.
right: incorrect model found in the structure of 4v9h. His 3 and leu 4 of bL17 have been incorrectly fitted due to an interpretable map in this region.
